# Cysteine‐Selective Modification of Peptides and Proteins via Desulfurative C−C Bond Formation

**DOI:** 10.1002/chem.202202503

**Published:** 2023-02-14

**Authors:** Rhys C. Griffiths, Frances R. Smith, Diyuan Li, Jasmine Wyatt, David M. Rogers, Jed E. Long, Lola M. L. Cusin, Patrick J. Tighe, Robert Layfield, Jonathan D. Hirst, Manuel M. Müller, Nicholas J. Mitchell

**Affiliations:** ^1^ School of Chemistry University of Nottingham University Park Nottingham NG7 2RD UK; ^2^ Department of Chemistry King's College London London SE1 1DB; ^3^ School of Life Sciences University of Nottingham Medical School Nottingham NG7 2UH UK; ^4^ School of Life Sciences University of Nottingham University Park Nottingham NG7 2RD UK

**Keywords:** bioconjugation, cysteine, desulfurization, post-translational modifications, site-selective

## Abstract

The site‐selective modification of peptides and proteins facilitates the preparation of targeted therapeutic agents and tools to interrogate biochemical pathways. Among the numerous bioconjugation techniques developed to install groups of interest, those that generate C(sp^3^)−C(sp^3^) bonds are significantly underrepresented despite affording proteolytically stable, biogenic linkages. Herein, a visible‐light‐mediated reaction is described that enables the site‐selective modification of peptides and proteins via desulfurative C(sp^3^)−C(sp^3^) bond formation. The reaction is rapid and high yielding in peptide systems, with comparable translation to proteins. Using this chemistry, a range of moieties is installed into model systems and an effective PTM‐mimic is successfully integrated into a recombinantly expressed histone.

## Graphical abstract

**A cysteine‐selective, visible‐light‐mediated bioconjugation** reaction that enables direct installation of a range of desired moieties into peptide and protein scaffolds via C(sp^3^)−C(sp^3^) bond formation is described. The method is rapid, operationally simple, tolerant to ambient atmosphere and can be utilized to incorporate general tools for chemical biology, including effective PTM mimics, via a stable, biogenic hydrocarbon linkage.

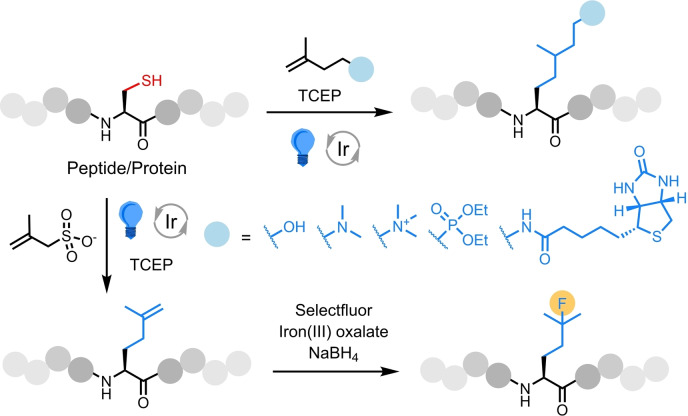

## Introduction

The preparation of proteins carrying non‐proteinogenic moieties or biologically relevant chemical modifications enables the engineering of polypeptide tools to interrogate biological activity or facilitate the production of targeted therapeutic/diagnostic agents.^[1]^ Several versatile methods have been developed to allow the introduction of non‐canonical residues into proteins to obtain homogeneously modified material. The technique of amber codon suppression is a powerful example of such efforts, permitting the incorporation of non‐standard amino acids via re‐engineering of recombinant protein expression.[^2^, ^3^] While this is an undeniably powerful strategy, extensive directed evolution of an appropriate aminoacyl‐^t^RNA synthetase is required. Alternative, fully synthetic approaches to this challenge facilitate the production of proteins carrying any desired modification at selected positions. Peptide ligation techniques such as native chemical ligation (NCL),[^4^, ^5^, ^6^, ^7^] diselenide‐selenoester ligation (DSL),[^8^, ^9^, ^10^, ^11^] and α‐ketoacid‐hydroxylamine (KAHA) ligation[^12^, ^13^, ^14^] afford access to modified proteins of up to approximately 300 amino acids in length.^[15]^ However, due to the synthetic effort required to perform multiple ligations, larger proteins are extremely challenging to produce via such techniques. Expressed protein ligation (EPL)[^16^, ^17^] offers a viable solution, however, the desired modification must be located close to the terminus of the sequence to enable the PTM to be incorporated using this technology.

**Figure 1:**
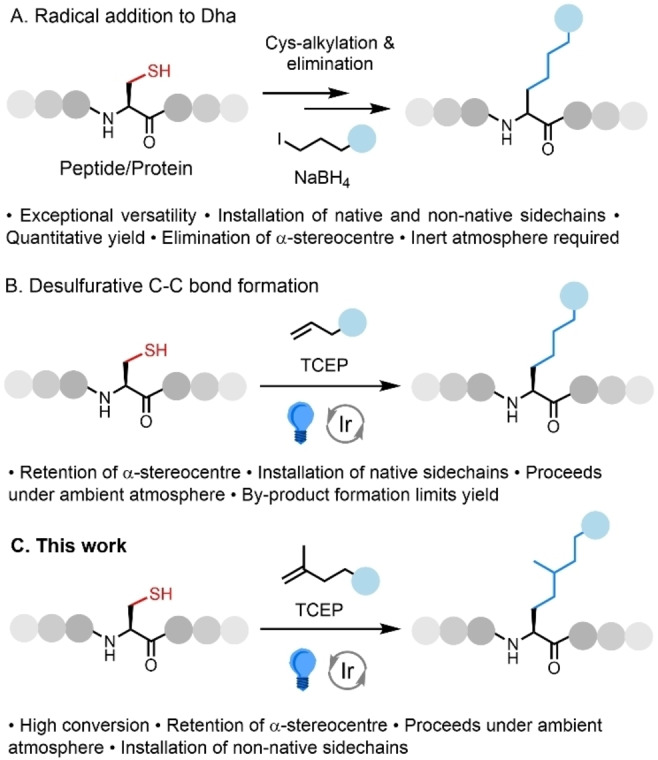
Cys‐selective peptide/protein modification via C(sp^3^)−C(sp^3^) bond formation.

A more generally accessible approach to the production of modified proteins is the targeted modification of the canonical amino acids.[^18^, ^19^, ^20^, ^21^] Due to the nucleophilicity of the thiol group of cysteine (Cys), and considering its relatively low abundance across eukaryotic proteomes (ca. 2 %), this residue is a convenient chemical handle with which to perform site‐selective chemistry.[^22^, ^23^] A broad variety of reactions have been developed to functionalize Cys including nucleophilic substitution[^22^, ^24^] and addition,[^25^, ^26^, ^27^, ^28^, ^29^, ^30^, ^31^] thiol‐ene chemistry,^[32]^ and metal‐free^[33]^/transition‐metal‐catalyzed^[34]^ arylation. These strategies exploit the nucleophilicity of the thiol group resulting in a C−S bond attaching the ‘cargo’ to the protein. While this is often an appropriate linkage for ‘standard’ protein bioconjugation (i.e., involving the conjugation of non‐proteinogenic groups), C(sp^3^)−C(sp^3^) bond formation directly onto the protein scaffold enables the installation of proteolytically/redox stable modifications and native PTMs, or effective PTM mimics. The current state‐of‐the‐art in this area involves alkyl radical addition onto Cys‐derived dehydroalanine (Dha) to allow the installation of a broad variety of desired groups via a hydrocarbon linkage (Figure 1A).[^35^, ^36^, ^37^]

Recently, we described an effective C(sp^3^)−C(sp^3^) bond forming method that involves the interception of visible‐light‐mediated desulfurization using *N*‐modified allylamines to install native lysine (Lys) sidechains carrying a broad range of PTMs (Figure 1B).^[38]^ The reaction proceeds via the generation and interception of an alanyl radical.[^39^, ^40^] This technique is operationally simple, it can be run on the bench under ambient atmosphere using inexpensive LEDs, and the product of the reaction retains the native stereochemistry at the α‐centre of the target amino acid. To push this pathway to completion we employ a large excess of the alkene, however, the reaction fails to fully out‐compete H‐atom abstraction by the alanyl radical. This results in the generation of a by‐product that bears the amino acid, alanine (Ala) at the site of modification. Seeking to improve the % conversion of our approach we opted to investigate the use of alternative traps to enhance the efficiency of the process and minimize or eliminate the formation of the Ala by‐product. We reasoned that an isoprenyl moiety would lead to the generation of a stabilized tertiary radical intermediate which should accelerate the rate of reaction sufficiently to out‐compete H‐atom abstraction (Figure 1C). While the installed linker would not result in a native Lys sidechain, it would likely act as an effective mimic. Furthermore, the technique would offer a versatile, stereoretentive reaction to enable the site‐selective modification of proteins with desired groups via C(sp^3^)−C(sp^3^) bond formation, a challenging objective even when applying modern synthetic techniques.^[20]^

## Results and Discussion

To investigate this approach, we synthesized peptide **1** (Ac‐CWHISKEY‐NH_2_, Table 1), a model which displays the majority of the chemical functionality found across the proteome, and subjected it to desulfurization conditions in the presence of isoprenol (**2**). Briefly, in 6 M Gdn ⋅ HCl, 0.1 M phosphate buffer pH 7.5–8 with 20 vol.% DMSO, peptide **1** (0.5 mM) was irradiated under blue LEDs with 5 mol% of the Ir(III) photocatalyst (Ir[dF(CF_3_)ppy]_2_(dtbpy))PF_6_ in the presence of a water soluble phosphine (TCEP, 2.5 mM) and 100 equiv. of isoprenol (**2**, 50 mM). The reaction was allowed to proceed for 60 mins after which time the crude material was analyzed by HPLC. We observed full consumption of the starting peptide over this period, with 61 % conversion to a product (quantified by analytical HPLC), identified as desired peptide **3** by MS (Entry 1; Table 1). The remaining material was found to be the Ala by‐product (Ac‐AWHISKEY‐NH_2_, confirmed using a peptide standard).

**Table 1:** Optimization of visible‐light‐mediated desulfurative C(sp^3^)−C(sp^3^) bond formation.

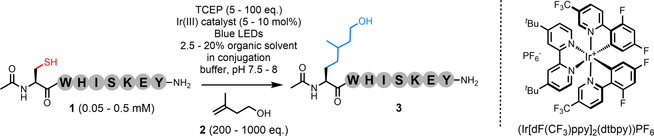						
Entry	Peptide **1** [mM]^[a]^	Isoprenol **2** [mM]	Catalyst [mol %]^[b]^	TCEP [mM]	Conversion (**1** to **3**) [%]	Time‐point of analysis [mins]
1	0.5	50	5	2.5	61	60
2	0.5	100	5	2.5	83 [79]^[d]^	60 [10]^[e]^
3	0.5	100	10^[c]^	2.5	73	60
4	0.5	50	5	25	46	60
5	0.05	100	5	2.5	0	60
6	0.05	500	5	50	87	60

[a] Solvent: 20 % DMSO in 6 M Gdn•HCl, 0.1 M phosphate, pH 8 (conjugation buffer, CB); no change in conversion down to 2.5 % DMSO, or when using MeOH or MeCN. [b] (Ir[dF(CF_3_)ppy]_2_(dtbpy))PF_6_ used for all entries except 3. [c] Eosin Y used as the initiator. [d] Figure in brackets shows isolated yield. [e] Reaction complete within 10 minutes (reaction monitored by analytical HPLC).

Increasing the concentration of isoprenol (**2**) to 100 mM (i. e., 200 equiv. over peptide **1**) enhanced the conversion of the reaction to 83 % (Table 1, Entry 2; hereafter referred to as protocol A). Protocol A was repeated at scale using **1**; the reaction was monitored and found to reach completion within just 10 mins. The desired product (**3**) was isolated by preparative HPLC in 79 % yield. The variables of this reaction were then further explored; the process was confirmed to only proceed under irradiation with blue light. Reactions that were removed from the photochemical set‐up stalled and did not progress until irradiation commenced (Figures S9–S12). The reaction was equally effective in 2.5–20 % organic solvent in denaturing buffer. However, conducting the reaction in phosphate buffered saline (PBS), with 10 % acetonitrile, reduced the conversion down to 63 % (Figure S22). Alternative initiators were investigated; the photodye, eosin Y (Entry 3) and Mn(OAc)_3_ (Table S1) did not improve the % conversion of the reaction. An increase in the concentration of TCEP from 2.5 mM to 25 mM had a detrimental effect on the conversion (Entry 4). No product formation was observed when the concentration of peptide **1** was decreased to 50 μM (Entry 5 and Table S4). However, an increase in the concentration of isoprenol (**2**) to 500 mM and TCEP to 50 mM reinstated the efficiency of the reaction at this concentration of peptide, leading to an 87 % conversion to desired product **3** (Entry 6).

With these results in hand, we repeated the reaction on model peptides Ac‐CAY‐NH_2_ (**4 a**) and Ac‐d‐CAY‐NH_2_ (**4 b**) and fully characterized the products to explore the integrity of the α‐stereocentre during the reaction. NMR analysis provided clear evidence that distinct diastereomers are formed for each reaction (**5 a** and **5 b**; Figure S19). Furthermore, to confirm that methionine (Met) was not oxidized over the course of the reaction under the described conditions, model Ac‐MACY‐NH_2_ (**6**) was subjected to the reaction with **2** to yield the desired product **7** with no sulfoxide by‐product detected (Figures S23 and S24).

To interrogate the reaction further, we conducted DFT calculations to model the formation of the intermediate tertiary radical species formed during this reaction, and the conversion of this species to the product (Supporting Information). We compared the relative energetics of this process to our previously reported reaction using trimethylated allylamine as the radical trap (proceeding via formation of a secondary radical species, Figure 1B).^[38]^ The gas phase calculations predicted that, as expected, the formation of the tertiary radical is more favorable than the secondary, and H‐atom abstraction by the more reactive secondary radical is favorable compared to that of the tertiary. However, the relative difference in energetics for these two processes is negated when the process is modelled in MeCN and water.

**Figure 2:**
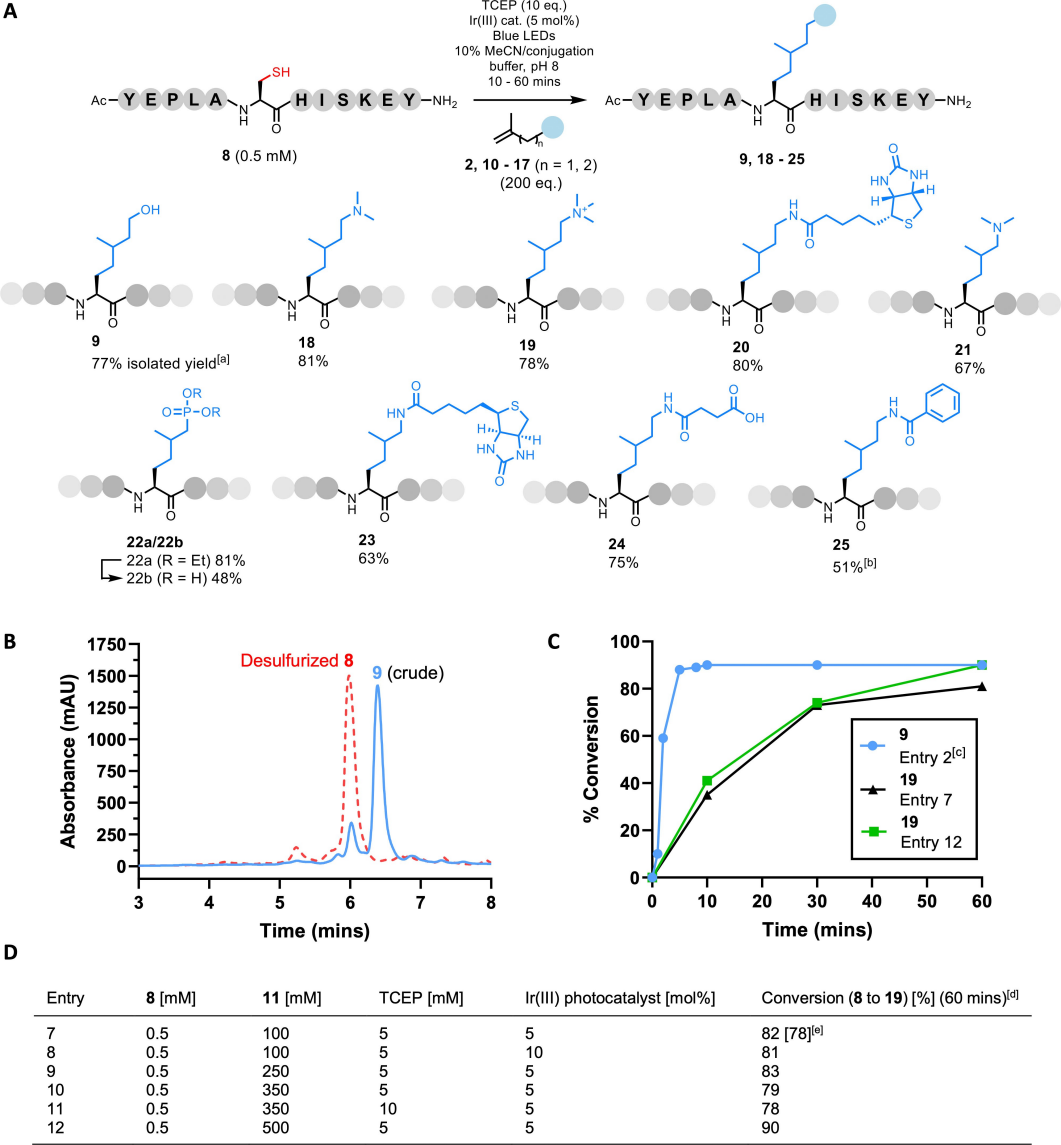
**A**. Cys‐selective installation of PTM mimics/desired groups via C(sp^3^)−C(sp^3^) bond formation. [a] Isolated yields shown using standard blue LEDs. [b] Trap **17** only partially soluble in reaction solvent. **B**. Crude HPLC trace for the reaction of **8** with **2** after 5 mins under protocol B conditions run in the PhotoRedOx Box. **C**. Time course of reaction progress using the PhotoRedOx Box. [c] Entry 2 conditions used for model peptide **8**. **D**. [d] Reactions run in the PhotoRedOx Box. [e] Isolated yield in brackets.

To explore the scope of the reaction with a variety of traps, and to test our chemistry on a more complex model system carrying the target Cys residue at an internal position within the sequence, we synthesized model peptide **8** (Ac‐YEPLACHISKY‐NH_2_; Figure 2A). Isoprenyl traps **10**–**17** were synthesized to enable the exploration of our method to the installation of methylated Lys sidechain mimics, hydrolytically stable phosphate/sulfate mimics (i.e., phosphonate and sulfonate groups), and moieties for broader application such as biotin (Supporting Information). Traps derived from isoprenol (**10**–**12**, **16**, and **17**) were compared to those carrying shorter hydrocarbon chains derived from isobutenol (**13** & **15**), and a commercially available phosphonate (**14**), to explore a linkage closer to that of a native Lys sidechain. For the more complex model **8**, a slightly higher concentration of TCEP (5 mM) was found to give superior conversion to the desired products (hereafter referred to as protocol B). The reaction of this model with isoprenol (**2**) under standard blue LEDs was observed to reach completion within 10 mins; product **9** was isolated in excellent yield (77 %). Satisfied that this chemistry is effective in complex systems, compounds **10**–**17** were conjugated to model **8** using protocol B. Under these conditions, the reactions utilizing the isoprenyl‐derived traps (**10**–**12**, and **16**) afforded the desired products **18**–**20**, and **24** in excellent isolated yield (75–81 %). Trap **17** afforded the desired product **25** in lower yield (51 %) due to low solubility of the trap. Reactions employing traps **10**, **12**, **16**, and **17** reached completion within 10 mins; trap **11** reached completion within 60 mins. The shorter traps based on isobutenol gave slightly lower yields under the described conditions (67 % and 63 % for **13** and **15**), while the phosphonate **14** afforded the desired product **22 a**, in an excellent 81 % yield. Each of these shorter traps reached completion within 30 mins (Figure 2A). To explore the installation of multiple modifications, the method was then applied to a peptide model carrying two Cys residues (**26**, Figure S7). This reaction yielded several by‐products demonstrating that, while effective for the installation of a single modification, the method is not suitable for multiple modifications (Figure S54).

We next evaluated the use of a PhotoRedOx Box (HepatoChem) with a 34 mW ⋅ cm^−2^ LED bulb (450 nm) to explore any potential enhancement in the reaction rate. The reaction between model **8** and isoprenol (**2**) was repeated using this apparatus under protocol B conditions. The % conversion to **9** was improved to 90 %, with remarkably clean generation of the product (Figure 2B). The rate of the reaction was also accelerated; complete consumption of **8** was achieved in under 5 mins (Figure 2C). Since we observed differences in yield and rate depending on the trap employed, we further explored the conjugation of the more recalcitrant trimethylammonium trap **11** using the PhotoRedOx Box. Initially, the same conditions were applied (protocol B); the conversion, isolated yield, and rate of reaction were found to be comparable to the reaction run using standard blue LEDs (Figure 2C; Figure 2D, Entry 7). Therefore, to push our chemistry to superior levels of conversion, we employed higher equiv. of **11** (Table S6). Only an increase in equiv. to 1000 had any appreciable effect, increasing the % conversion to 90 % over 60 mins (Figure 2C; Figure 2D, Entry 12). While this is clearly a significant excess, the large disparity in mass between the alkene and a complex peptide or protein translates into only milligrams of trap material. Crucially, high conversion to the desired product simply requires small molecule reagents to be separated from the protein material using HPLC, SEC, or dialysis.

**Figure 3:**
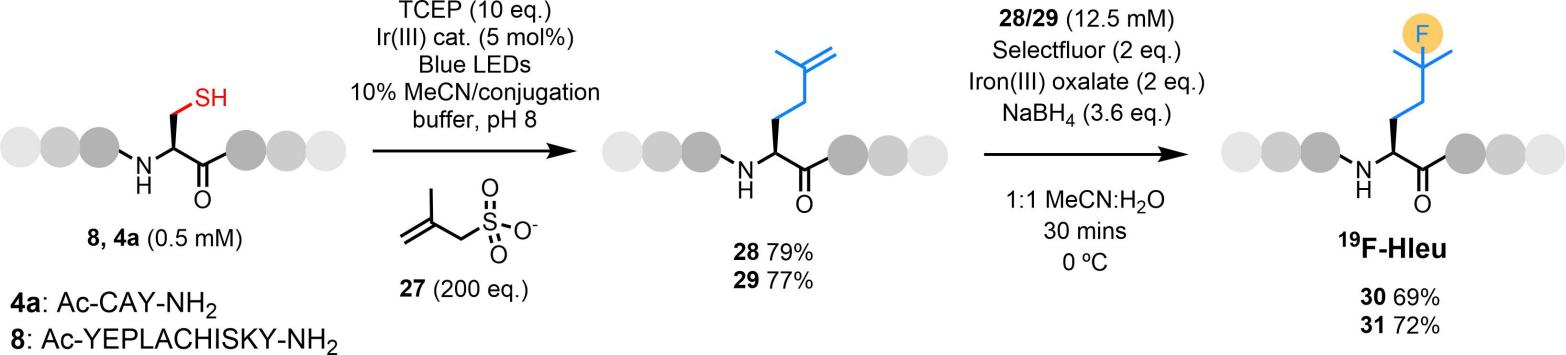
Installation of a sulfonate group and elimination to afford an isoprenyl handle followed by site‐selective fluorination.

In addition to the exploration of the groups shown in Figure 2A, we also sought to install a hydrolytically stable sulfonate moiety via the commercially available compound **27** (Figure 3). Upon consumption of the starting peptide **8**, we were surprised to isolate a peptide that appeared to carry the isoprenyl modification at the target position as the product in good yield (**28**, 79 %, Figure S46). To confirm this result, we repeated the reaction on the small model peptide **4 a** and isolated product **29** in 77 % yield (Figure S50). This product was submitted to NMR analysis which confirmed the presence of the non‐proteinogenic isoprenyl sidechain. While this was not the intended result, the site‐selective installation of this group affords us a versatile bio‐orthogonal handle to directly functionalise the polypeptide scaffold. As an example application, we chose hydrofluorination; the ability to install fluorine into peptides and proteins in a rapid, site‐selective, late‐stage manner enables the development of NMR tools (via incorporation of fluorine‐19) and PET imaging agents (using fluorine‐18). Adapting literature methods using Selecfluor, iron(III) oxalate, and NaBH_4_,^[41]^ we were able to selectively fluorinate this alkene within 30 mins for both products **28** and **29** to afford peptides **30** and **31** in excellent isolated yield (Figures 3, S48 & S52). Direct fluorination of the protein scaffold is desirable as it results in minimal perturbation of the folded structure, however, this is challenging to achieve. Fluorination of the installed isoprenyl group results in a labelled homoleucine (Hleu) residue; thus our method is an excellent alternative to the use of bulky prosthetic groups for selective labelling.

**Figure 4:**
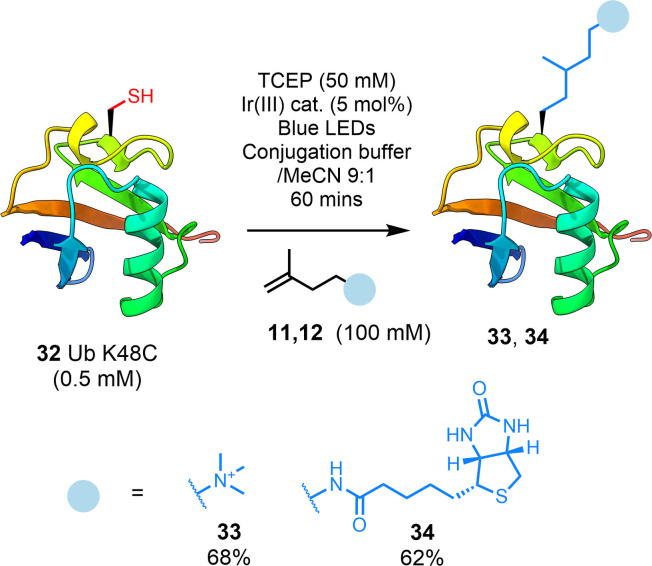
Modification of ubiquitin (Ub) K48C (**32**) with trimethylammonium (**11**) and biotin (**12**) traps.

Finally, we explored the application of our chemistry to the modification of proteins. A K48C mutant of ubiquitin (Ub; **32**) was employed to demonstrate the site‐selective installation of a PTM (trimethylammonium; compound **11**) and an isolation tag (biotin; compound **12**) into a small protein (Figure 4). We applied the initial protocol B conditions utilizing the inexpensive blue LED set up to demonstrate the accessibility of our method to protein functionalization. These conditions did not afford an acceptable conversion; therefore, we increased the concentration of TCEP in the reaction to 50 mM. Under these new conditions, the reactions proceeded to completion within 60 mins. Thus, the protocol B conditions were adapted using 50 mM TCEP (protocol C) and the reaction repeated on an isolatable scale. As Ub is a small protein, the desired products **33** and **34** could be isolated from any protein by‐products using preparative HPLC in excellent yield considering the complexity of the scaffold (68 % and 62 % respectively). Modified Ub (**33**) was refolded after purification affording an identical CD spectrum to **32** (Figures S61 & S62).

**Figure 5:**
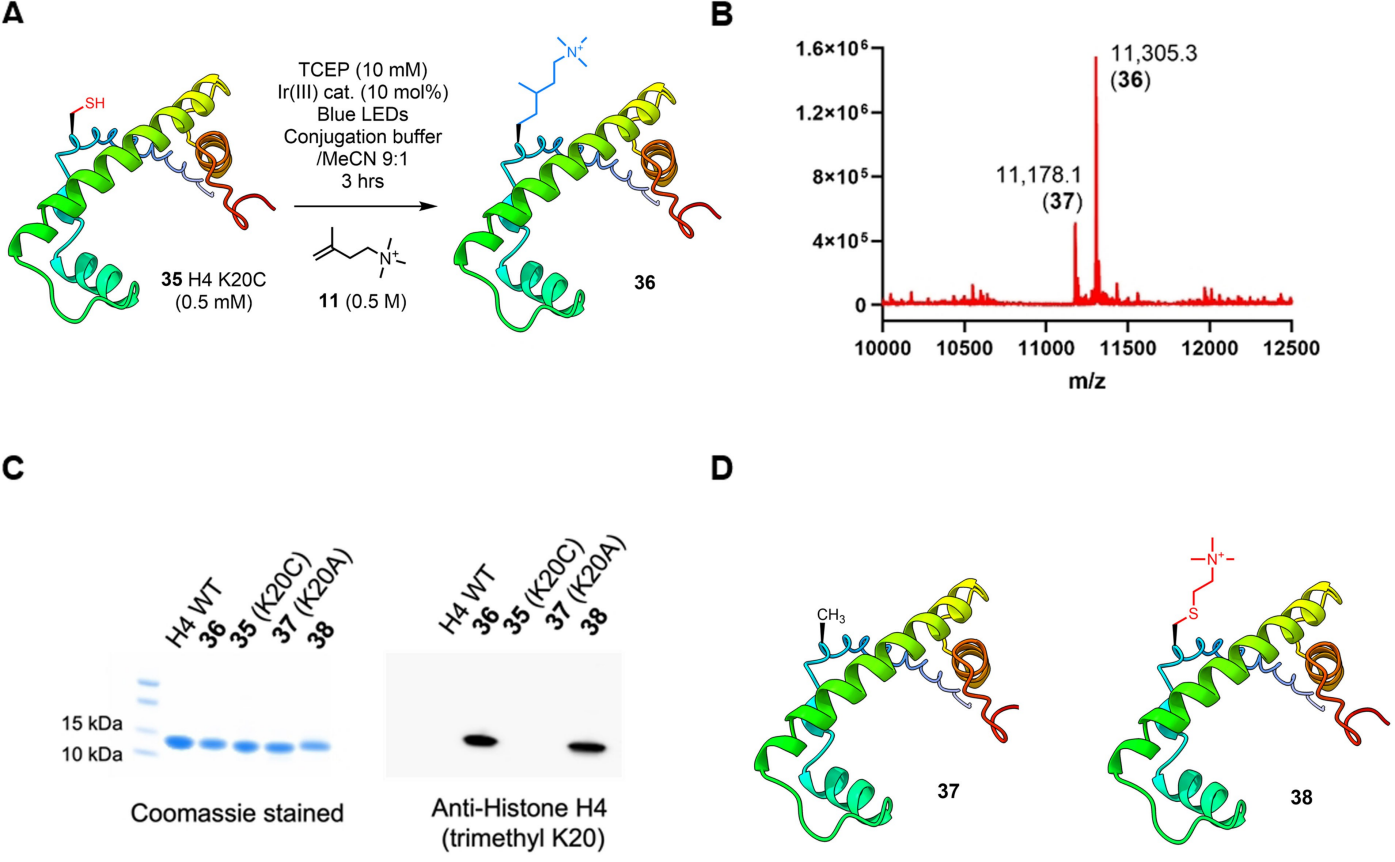
**A**. Installation of a trimethyl Lys sidechain into histone H4K20C (**35**). **B**. Deconvoluted ESI MS of crude material shows the majority desired product (**36**; calculated mass [M+H]^+^ 11,305.4) and the minority presence of the undesired by‐product carrying Ala at the target site (**37**; calculated mass [M+H]^+^ 11,178.3). **C**. Western blot using anti‐histone H4 (trimethyl K20) antibody to demonstrate effective recognition of mimics **36** and **38**. **D**. Desulfurized protein **37** and mimic **38**.

To demonstrate that the non‐native sidechains installed via our method act as effective PTM mimics, we then applied this reaction to introduce a biologically relevant modification into a histone H4 protein. Methylation of H4 is a well‐studied PTM known to modulate chromatin structure. Due to the size of the histone (102 mer) the desired product cannot be isolated from minority protein by‐products, thus it is vital that the reaction proceeds as close to complete conversion as possible. Application of protocol C to a K20C mutation of H4 (**35**, prepared via expression in *E. coli*; Figures S71 & S72) led to the generation of unidentified by‐products. Thus, based on the results summarized in Figure 2D, we irradiated a 0.5 mM solution of **35** in the presence of TCEP (10 mM), compound **11** (0.5 M), with 10 mol% of the Ir(III) catalyst for 3 h (hereafter referred to as protocol D). The material was then passed down an HPLC column to separate out the excess regents (all protein material co‐eluted as expected; note that dialysis or SEC would have been equally effective for this purpose). The desired product was identified as the majority mass by ESI MS (**36**), with a minority of Ala by‐product present (Figure 5B and Supporting Information). We performed a western blot to confirm that modified protein **36** is recognized by a trimethyllysine‐specific antibody. The WT H4 protein, starting material (H4K20C, **35**), desulfurised by‐product (**37**), and a known trimethyl‐thialysine analog (**38**) were included as controls. The latter was obtained via Cys alkylation and is routinely employed as an effective mimic of trimethyllysine.^[24]^ Our results show that both trimethyllysine mimics **36** and **38** were recognized by the antibody (Figure 5C) demonstrating that the branch and additional methylene group in our product do not interfere with molecular recognition between the antibody and the modified histone, which augurs well for application of our new analogue as a Lys PTM mimic. Importantly, the aliphatic chain is resistant to oxidation and alkylation ‐ a frequent problem encountered with thioether containing analogues.^[42]^

## Conclusion

In conclusion, we have developed an operationally simple and site‐selective method for the installation of desired moieties into peptides and proteins via desulfurative C(sp^3^)−C(sp^3^) bond formation. This visible‐light‐induced reaction proceeds efficiently under ambient atmosphere to enable the modification of peptides and proteins via a redox‐ and hydrolytically/proteolytically stable hydrocarbon linkage without epimerization of the target residue.

We demonstrate the application of this chemistry as a general bioconjugation method as well as a technique to enable the site‐selective introduction of effective Lys PTMs. This advance improves upon the yields we previously reported using *N*‐modified allylamine as the trap for our desulfurative bioconjugation chemistry.^[38]^ The operational simplicity of the described protocols should ensure accessibility for researchers working across a variety of disciplines.

## Experimental Section

**General**: NMR samples were analyzed on either a Bruker AVIII 400 NMR system (^1^H NMR frequency 400 MHz; ^13^C NMR frequency 100 MHz) or a Bruker 500 MHz system (^1^H NMR frequency 500 MHz; ^13^C NMR frequency 125 MHz). Chemical shifts are reported in parts per million (ppm) and are referenced to solvent residual signals: CDCl_3_ (δ 7.26 [^1^H]), DMSO (δ 2.50 [^1^H]), MeOD (δ 3.31 [^1^H]). ^1^H NMR data is reported as chemical shift (δ), multiplicity (s=singlet, d=doublet, t=triplet, q=quartet, or combinations of these splitting patterns; m=unassigned multiplet), relative integral and coupling constant (J Hz). ^13^C NMR data is reported as chemical shift (δ) and classification of the carbon (e. g., CH_3_/CH_2_/CH/C). High‐resolution mass spectra were recorded on a Bruker MicroTOF Focus II MS (ESI) operating in positive or negative ionisation mode. Analytical HPLC was performed on a Thermo Ultimate 3000 μHPLC system equipped with PDA eλ detector (λ=210–400 nm). Peptides were analyzed using a Waters Sunfire 5 μm, 2.1×150 mm column (C‐18) at a flow rate of 0.6 mL⋅min^−1^. The mobile phase composed of 0.1 % trifluoroacetic acid in H_2_O (Solvent A) and 0.1 % trifluoroacetic acid in acetonitrile (Solvent B) using the gradients specified in the Supporting Information. The analysis of the chromatograms was conducted using Chromeleon 7 software. Preparative reverse‐phase HPLC was performed using a Waters 1525 binary pump HPLC equipped with a dual wavelength UV detector set to 210 nm and 280 nm. Peptides were purified on a Waters Sunfire 5 μm, 19×150 mm (C‐18) preparative column, operating at a flow rate of 6 mL⋅min^−1^ using a mobile phase of 0.1 % trifluoroacetic acid in water (Solvent A) and 0.1 % trifluoroacetic acid in acetonitrile (Solvent B) using the gradients specified in the Supporting Information. Semi‐preparative reverse‐phase HPLC was performed using the same HPLC and solvent system. The column used was a Waters Sunfire 5 μm, 10×250 mm (C‐18) semi‐preparative column, operating at a flow rate of 5 mL⋅min^−1^ using the gradients specified in the Supporting Information. Circular dichroism was carried out using an Applied Photophysics Chirascan Plus.

**Materials**: Commercial materials were used as received unless otherwise noted. Amino acids, coupling reagents and resins were obtained from Novabiochem, Fluorochem or GL Biochem. Fmoc‐protected, l‐amino acids were purchased with appropriate acid‐labile sidechain protecting groups. Reagents that were not commercially available were synthesized as outlined in the Supporting Information. Solvents were obtained as reagent grade from Merck or Fisher.

## Solid Phase Peptide Synthesis (SPPS)

### Manual Fmoc‐SPPS

*Preloading Rink Amide resin*: Rink amide resin was initially washed with DCM (5×3 mL) followed by removal of the Fmoc group by treatment with 20 % piperidine/DMF (2×5 min). The resin was washed with DMF (5×3 mL), DCM (5×3 mL) and DMF (5×3 mL). Oxyma Pure (4 equiv.) and DIC (4 equiv.) were added to a solution of Fmoc‐AA‐OH (4 equiv.) in DMF. After 5 min of pre‐activation, the mixture was added to the resin. After 2 h the resin was washed with DMF (5×3 mL), DCM (5×3 mL) and DMF (5×3 mL), capped with acetic anhydride/pyridine (1 : 9 v/v) (2×3 min) and washed with DMF (5×3 mL), DCM (5×3 mL) and DMF (5×3 mL).

*Preloading 2‐chlorotrityl chloride resin*: 2‐Chlorotrityl chloride resin was swollen in DCM for 30 min then washed with DCM (2×3 mL). A solution of Fmoc‐AA‐OH (0.5 equiv. relative to resin functionalization) and *i*Pr_2_NEt (2.0 equiv. relative to resin functionalization) in DCM (final concentration 0.1 M of amino acid) was added and the resin shaken at rt for 16 h. The resin was washed with DMF (5×3 mL) and DCM (5×3 mL). The resin was treated with a solution of DCM/CH_3_OH/*i*Pr_2_NEt (17 : 2 : 1 v/v/v, 3 mL) for 1 h and washed with DMF (5×3 mL), DCM (5×3 mL), and DMF (5×3 mL).

*Estimation of amino acid loading*: The resin was treated with 20 % piperidine/DMF (2×3 mL, 3 min) and 20 μL of the combined deprotection solution was diluted to 10 mL using 20 % piperidine/DMF in a volumetric flask. The UV absorbance of the resulting piperidine‐fulvene adduct was measured (λ=301 nm, ϵ=7800 M^−1^ ⋅ cm^−1^) to determine loading of the resin.

*General amino acid coupling*: A solution of Fmoc‐AA‐OH (4 equiv.), DIC (4 equiv.) and Oxyma Pure (4 equiv.) in DMF (final concentration 0.1 M) was added to the resin. After 1 h, the resin was washed with DMF (5×3 mL), DCM (5×3 mL) and DMF (5×3 mL).

*Capping*: Acetic anhydride/pyridine (1 : 9 v/v) was added to the resin (3 mL). After 3 min the resin was washed with DMF (5×3 mL), DCM (5×3 mL) and DMF (5×3 mL).

*Deprotection*: The resin was treated with 20 % piperidine/DMF (2×3 mL, 3 min) and washed with DMF (5×3 mL), DCM (5×3 mL) and DMF (5×3 mL).

*Cleavage*: A mixture of TFA, thioanisole, tri*iso*propylsilane (TIS) and water (90 : 4 : 4 : 2 v/v/v/v) was added to the resin. After 3 h, the resin was washed with TFA (3×2 mL).

*Work‐up*: The combined cleavage solutions were concentrated under a stream of nitrogen to <5 mL. 40 mL of diethyl ether was added to precipitate the peptide and the suspension centrifuged. The pellet was then dissolved in water containing 0.1 % TFA, filtered and purified by preparative HPLC and analyzed by LC‐MS and ESI mass spectrometry.

**Automated solid‐phase peptide synthesis**: Automated Fmoc‐SPPS was carried out on either a Biotage Initiator^+^ Alstra or CEM Liberty Blue microwave peptide synthesizer. General synthetic procedures for Fmoc‐deprotection and capping were carried out in accordance with the manufacturer's specifications. Biotage Initiator^+^ Alstra: standardized amino acid couplings were performed for 15 min at 50 **°**C under microwave irradiation in the presence of amino acid (0.5 M in DMF, 4 equiv.), Oxyma Pure (0.5 M in DMF, 4 equiv.) and di*iso*propylcarbodiimide (0.5 M in DMF, 4 equiv.). Peptide cleavage and work‐up were carried out as described for manual SPPS. CEM Liberty Blue: standardized amino acid couplings were performed for 2.5 min at 90 °C under microwave irradiation in the presence of amino acid (0.2 M in DMF, 4 equiv.), Oxyma Pure (1 M in DMF, 4 equiv.) and di*iso*propylcarbodiimide (1 M in DMF, 4 equiv.). Peptide cleavage and work‐up were carried out as described for manual SPPS.

### Photochemistry apparatus

**Set up 1**: A blue LED light strip wrapped around a pyrex dish was placed on top of a stirrer plate. To ensure consistency, places for up to 4 vials were marked on the plate. The temperature was monitored and observed to reach no higher than 30 °C.

**Set up 2**: PhotoRedOx Box (HCK1006‐01‐016, HepatoChem) operated with a 450 nm, 34 mW/cm^2^ bulb (450PF, HCK1012‐01‐002, Hapatochem) at room temp.

### Visible‐light‐mediated desulfurative C‐C bond formation

**General Protocol**: To peptide dissolved in 10 % acetonitrile (MeCN) in conjugation buffer (CB: 6 M Gdn ⋅ HCl, 0.1 M phosphate, pH 7.5) to a concentration of 1 mM was added a solution of TCEP (0.5 M stock solution in CB pH adjusted to 7–8), alkene trap and (Ir[dF(CF_3_)ppy]_2_(dtbpy))PF_6_ (1 mM stock solution in MeCN). The pH of the reaction mixture was checked to be 7.5‐8 then the reaction mixture diluted to the final peptide concentration of 0.5 mM. The reaction vessel was then placed into blue LEDs/PhotoRedOx Box; once the starting material was shown to be fully consumed by HPLC the reaction mixture was purified by semi‐preparative HPLC.

## Conflict of interest

The authors declare no conflict of interest.

1

## Supporting information

As a service to our authors and readers, this journal provides supporting information supplied by the authors. Such materials are peer reviewed and may be re‐organized for online delivery, but are not copy‐edited or typeset. Technical support issues arising from supporting information (other than missing files) should be addressed to the authors.

Supporting Information

## Data Availability

The data that support the findings of this study are available in the supplementary material of this article.
