# Supplementary material for: Cysteine‐Selective Modification of Peptides and Proteins via Desulfurative C−C Bond Formation
Source: Chemistry. 2023 Feb 14;29(16):e202202503. doi: 10.1002/chem.202202503 (PMC10946470; doi:10.1002/chem.202202503)
Supplement: Supplementary file 1 — Supporting Information [file CHEM-29-0-s001.pdf]

# Chemistry–A European Journal

Supporting Information


## Cysteine-Selective Modification of Peptides and Proteins via Desulfurative C–C Bond Formation


Rhys C. Griffiths, Frances R. Smith, Diyuan Li, Jasmine Wyatt, David M. Rogers, Jed E. Long, Lola M. L. Cusin, Patrick J. Tighe, Robert Layfield, Jonathan D. Hirst, Manuel M. Müller, and Nicholas J. Mitchell*

## Contents


General Methods ........ 2

- Materials ........ 3
- Solid Phase Peptide Synthesis (SPPS) ........ 3
- Manual Fmoc-SPPS ........ 3
- Automated solid-phase peptide synthesis ........ 4
- Photochemistry apparatus ........ 5

Visible-light-mediated desulfurative C-C bond formation ........ 7

Compound synthesis ........ 9

Peptide Synthesis ........ 15

Optimization of visible-light-mediated conjugation ........ 21

Progress of the reaction in the absence of blue light ........ 24

NMR analysis of conjugation ........ 27

Exploration of reaction scope ........ 33

Modification of ubiquitin ........ 70

Reaction optimisation to minimise by-product formation ........ 76

Modification of histone H4 ........ 81

DFT on peptide radicals to compute reaction free energies ........ 89

## General Methods

NMR samples were analysed on either a Bruker AVIII 400 NMR system ($^1$H-NMR frequency 400 MHz; $^{13}$C-NMR frequency 100 MHz) or a Bruker 500 MHz system ($^1$H-NMR frequency 500 MHz; $^{13}$C-NMR frequency 125 MHz). Chemical shifts are reported in parts per million (ppm) and are referenced to solvent residual signals: $CDCl_3$ (δ 7.26 [$^1$H]), DMSO (δ 2.50 [$^1$H]), MeOD (δ 3.31 [$^1$H]). $^1$H NMR data is reported as chemical shift (δ), multiplicity (s = singlet, d = doublet, t = triplet, q = quartet or combinations of these splitting patterns; m = unassigned multiplet), relative integral, and coupling constant (J Hz). $^{13}$C NMR data is reported as chemical shift (δ) and classification of the carbon (e.g., $CH_3$/$CH_2$/CH/C).

High-resolution mass spectra were recorded on a Bruker MicroTOF Focus II MS (ESI) operating in positive or negative ionisation mode. Analytical HPLC was performed on a Thermo Ultimate 3000 μHPLC system equipped with PDA eλ detector (λ = 210 – 400 nm). Peptides were analyzed using a Waters Sunfire 5 µm, 2.1 x 150 mm column (C-18) at a flow rate of 0.6 mL min$^{-1}$. The mobile phase composed of 0.1% trifluoroacetic acid in $H_2O$ (Solvent A) and 0.1% trifluoroacetic acid in acetonitrile (Solvent B). The analysis of the chromatograms was conducted using Chromeleon 7 software. Reaction conversions (%) were calculated from the integrals of all peptide peaks in the analytical HPLC spectra of the crude reaction mixtures.

Preparative reverse-phase HPLC was performed using a Waters 1525 binary pump HPLC equipped with a dual wavelength UV detector set to 210 nm and 280 nm. Peptides were purified on a Waters Sunfire 5 µm, 19 x 150 mm (C-18) preparative column operating at a flow rate of 6 mL min$^{-1}$ using a mobile phase of 0.1% trifluoroacetic acid in water (Solvent A) and 0.1% trifluoroacetic acid in acetonitrile (Solvent B) using the gradient specified. Semi-preparative reverse-phase HPLC was performed using the same HPLC and solvent system. The column used was a Waters Sunfire 5 µm, 10 x 250 mm (C-18) preparative column, operating at a flow rate of 5 mL min$^{-1}$ using the gradient specified.

Circular dichroism was carried out using an Applied Photophysics Chirascan Plus.

## Materials

Commercial materials were used as received unless otherwise noted. Amino acids, coupling reagents and resins were obtained from Novabiochem, Fluorochem or GL Biochem. Reagents that were not commercially available were synthesized as outlined. Solvents were obtained as reagent grade from Merck or Fisher.

## Solid Phase Peptide Synthesis (SPPS)

### Manual Fmoc-SPPS

*Preloading Rink Amide resin:* Rink amide resin was initially washed with DCM (5 × 3 mL) followed by removal of the Fmoc group by treatment with 20% piperidine/DMF (2 × 5 min). The resin was washed with DMF (5 × 3 mL), DCM (5 × 3 mL) and DMF (5 × 3 mL). Oxyma Pure (4 eq.) and DIC (4 eq.) were added to a solution of Fmoc-AA-OH (4 eq.) in DMF. After 5 min of pre-activation, the mixture was added to the resin. After 2 h the resin was washed with DMF (5 × 3 mL), DCM (5 × 3 mL) and DMF (5 × 3 mL), capped with acetic anhydride/pyridine (1:9 v/v) (2 × 3 min) and washed with DMF (5 × 3 mL), DCM (5 × 3 mL) and DMF (5 × 3 mL).

*Preloading 2-chlorotrityl chloride resin:* 2-Chlorotrityl chloride resin was swollen in DCM for 30 min then washed with DCM (2 × 3 mL). A solution of Fmoc-AA-OH (0.5 equiv. relative to resin functionalization) and *i*$Pr_2$NEt (2.0 eq. relative to resin functionalization) in DCM (final concentration 0.1 M of amino acid) was added and the resin shaken at rt for 16 h. The resin was washed with DMF (5 × 3 mL) and DCM (5 × 3 mL). The resin was treated with a solution of DCM/$CH_3OH$/*i*$Pr_2$NEt (17:2:1 v/v/v, 3 mL) for 1 h and washed with DMF (5 × 3 mL), DCM (5 × 3 mL), and DMF (5 × 3 mL).

*Estimation of amino acid loading:* The resin was treated with 20% piperidine/DMF (2 x 3 mL, 3 min) and 20 µL of the combined deprotection solution was diluted to 10 mL using 20% piperidine/DMF in a volumetric flask.

The UV absorbance of the resulting piperidine-fulvene adduct was measured ($\lambda$ = 301 nm, $\varepsilon$ = 7800 $M^{-1}$ $cm^{-1}$) to determine the loading of the resin.

*General amino acid coupling:* A solution of protected amino acid (4 eq.), DIC (4 eq.) and Oxyma Pure (4 eq.) in DMF (final concentration 0.1 M) was added to the resin. After 1 h, the resin was washed with DMF (5 × 3 mL), DCM (5 × 3 mL) and DMF (5 × 3 mL).

*Deprotection:* The resin was treated with 20% piperidine/DMF (2 x 3 mL, 3 min) and washed with DMF (5 × 3 mL), DCM (5 × 3 mL) and DMF (5 × 3 mL).

*Capping*: Acetic anhydride/pyridine (1:9 v/v) was added to the resin (3 mL). After 3 min the resin was washed with DMF (5 × 3 mL), DCM (5 × 3 mL) and DMF (5 × 3 mL).

*Cleavage*: A mixture of TFA, thioanisole, tri*iso*propylsilane (TIS) and water (90:4:4:2 v/v/v/v) was added to the resin. After 3 h, the resin was washed with TFA (3 × 2 mL).

*Work-up*: The combined cleavage solutions were concentrated under a stream of nitrogen to < 5 mL. 40 mL of diethyl ether was added to precipitate the peptide and the suspension centrifuged. The pellet was then dissolved in water containing 0.1% TFA, filtered and purified by preparative HPLC and analyzed by LC-MS and ESI mass spectrometry.

**Automated solid-phase peptide synthesis**

Automated Fmoc-SPPS was carried out on either a Biotage Initiator$^+$ Alstra or CEM Liberty Blue microwave peptide synthesizer. General synthetic procedures for Fmoc-deprotection and capping were carried out in accordance with the manufacturer's specifications. Biotage Initiator$^+$ Alstra: standardized amino acid couplings were performed for 15 min at 50 °C under microwave irradiation in the presence of amino acid (0.5 M in DMF, 4 eq.), Oxyma Pure (0.5 M in DMF, 4 eq.) and di*iso*propylcarbodiimide (0.5 M in DMF, 4 eq.). Peptide cleavage and work-up were carried out as described above for manual SPPS. CEM Liberty Blue: standardized amino acid couplings were performed for 2.5 min at 90 ºC under microwave irradiation in the presence

of amino acid (0.2 M in DMF, 4 eq.), Oxyma Pure (1 M in DMF, 4 eq.) and di*iso*propylcarbodiimide (1 M in DMF, 4 eq.). Peptide cleavage and work-up were carried out as described above for manual SPPS.

**Photochemistry apparatus**

**Set up 1** A blue LED light strip, wrapped around a pyrex dish was placed on top of a stirrer plate. To ensure consistency, places for up to 4 vials were marked on the plate. The temperature was monitored and observed to reach no higher than 30 °C.

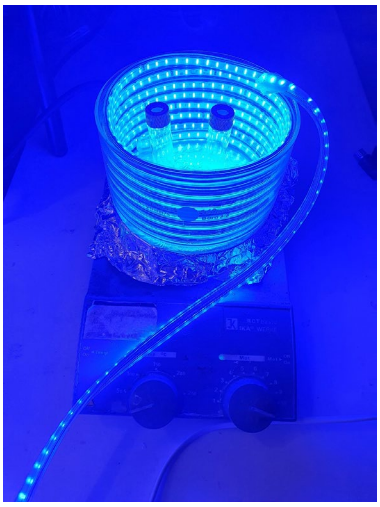

**Set up 2** To compare set up 1 with specialized equipment, all reactions were also carried out in a temperature controlled PhotoRedOx Box (HCK1006-01-016, HepatoChem) operated with a 450 nm, 34 mW/cm$^2$ bulb (450PF, HCK1012-01-002, Hapatochem) at room temp.

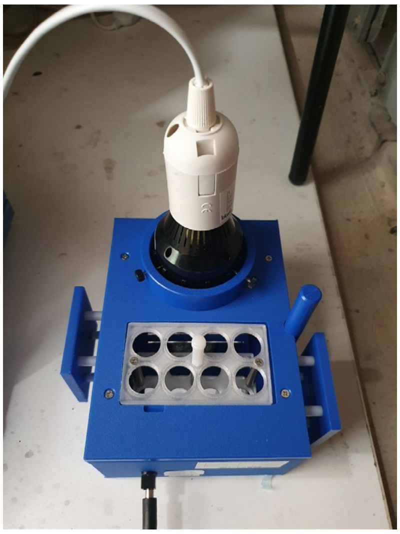

**Computational Modelling**

Initial 3D structures for the two radicals and the two products were generated from Smiles strings. These structures were input to Open Babel[1,2] to generate low energy conformers using the genetic algorithm with the energy score search criterion. For both products, the 10 lowest energy conformers, evaluated by the MMFF94 force field[3] from the Open Babel search, were optimised at the PM7 level of theory, as modified by Throssel and Frisch,[4] with minimum energy stationary points confirmed by harmonic vibrational frequency calculation.

Two low energy structures for each product, from the PM7 calculations, were optimised using Density Functional Theory (DFT) with the PBE0 functional[5] and the Def2-SVP basis set.[6] These DFT optimised structures were used, by removing one hydrogen atom from each structure, to generate the intermediate radical structures that were optimised at the PBE0/Def2-SVP level of theory. The alanyl radical and both amines were optimised at the PBE0/Def2-SVP level of theory along with the energy of the hydrogen atom. The Polarisable Continuum Model (PCM)[7] was employed to mimic bulk solvents of acetonitrile (dielectric constant 35.688) and water (dielectric constant 78.3553). For all DFT calculations, minimum energy stationary points were confirmed by harmonic vibrational frequency calculation.

Gaussian 16[8] was employed for the PM7 calculations and the DFT calculations in gas phase and in solvent described by the PCM.

## Visible-light-mediated desulfurative C-C bond formation

### General Protocol

To peptide dissolved in 10% acetonitrile (ACN) in conjugation buffer (CB: 6M Gdn•HCl, 0.1M phosphate, pH 7.5) to a concentration of 1 mM was added a solution of TCEP (0.5 M stock solution in CB pH adjusted to 7 - 8), alkene trap and (Ir[dF($CF_3$)ppy]$_2$(dtbpy))$PF_6$ (1 mM stock solution in ACN). The pH of the reaction mixture was checked to be 7.5- 8 then the reaction mixture diluted to the final peptide concentration of 0.5 mM. The reaction vessel was then placed into blue LEDs/PhotoRedOx Box; once the starting material was shown to be fully consumed by HPLC the reaction mixture was purified by semi-preparative HPLC.

| Protocol | Alkene (equiv.) | TCEP (equiv.) | Ir(III) catalyst |
|---|---|---|---|
| A | 200 | 5 | 5 mol% |
| B | 200 | 10 | 5 mol% |
| C | 200 | 100 | 5 mol% |
| D | 1000 | 20 | 10 mol% |

## Cys-selective visible-light-mediated desulfurative C-C bond forming chemistry

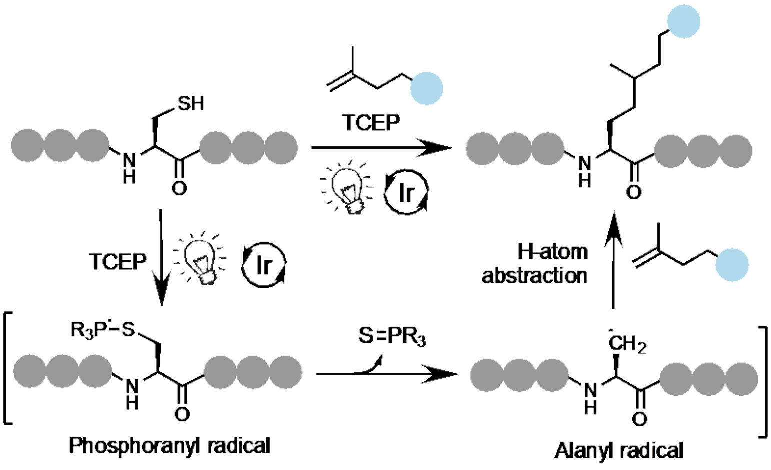


**Figure S1.** Site-selective polypeptide modification via C($sp^3$)-C($sp^3$) bond formation. Generation of an alanyl radical using a photocatalyst and light in the presence of a phosphine; interception of the radical using bespoke isoprenyl traps.

## Compound synthesis

Compounds **2**, **14**, and **27** are commercially available and were used as received.

**Isoprenol (2)**

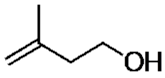

**Diethyl(2-methylallyl)phosphonate (14)**

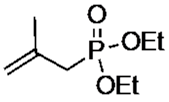

**2-Methyl-2-propene-1-sulfonic acid (27)**

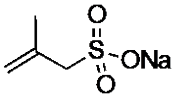

**3-methyl-3-buten-1-amine (39)**

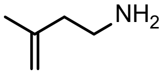

To a solution of 3-methyl-3-buten-1-ol (isoprenol, **2**; 5.86 mL, 58.1 mmol) and mesyl chloride (13.5 mL, 174 mmol) in DCM (50 mL) at 0 °C was added triethylamine (24.3 mL, 174 mmol) dropwise. After addition the reaction mixture was allowed to warm to room temperature and stirred for 4 hours. The reaction mixture was then diluted with DCM (50 mL) and washed with 1 M HCl (3 x 20 mL), sat. $NaHCO_3$ (3 x 20 mL) and brine (20 mL), dried over $MgSO_4$, filtered and concentrated to give the crude mesylate (9.4 g, 56.9 mmol, 98% yield). To a portion of the crude mesylate (2.0 g, 12.2 mmol) was added ammonia solution (30 mL, 304 mmol, 35% solution in water) followed by ammonium acetate (18.8 g, 244 mmol). The solution was stirred at room temperature for 48 hours before being saturated with potassium carbonate and extracted with diethyl ether (5 x

20 mL). The combined organic extracts were dried over $MgSO_4$, filtered and purified by distillation (85 °C elution) to give the title compound as a colourless liquid (0.8 g, 9.4 mmol, 77% yield). $^1$H NMR (400 MHz, $D_2O$) δ 4.77 (t, J = 1.9 Hz, 1H), 2.65 (t, J = 6.8 Hz, 2H), 2.10 (t, J = 6.8 Hz, 2H), 1.65 (d, J = 1.4 Hz, 3H). Second alkene proton singlet overlaps with $D_2O$ peak. $^{13}$C NMR (101 MHz, $D_2O$) δ 144.7 (C), 111.3 ($CH_2$), 39.8 ($CH_2$), 38.1 ($CH_2$), 21.2 ($CH_3$). IR (ATR, $cm^{-1}$) - 3364brw, 3291brw, 3074w, 2986m, 2934w, 1648m, 1598w, 1442m, 1375m, 1056w, 884s.

***N,N*-3-trimethylbut-3-en-1-amine (10)**

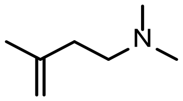


To a solution of 3-methyl-3-buten-1-ol (isoprenol, **2**; 5.86 mL, 58.1 mmol) and mesyl chloride (13.5 mL, 174 mmol) in DCM (50 ml) at 0 ºC was added triethylamine (24.3 mL, 174 mmol) dropwise. After addition the reaction mixture was allowed to warm to room temperature and stirred for 4 hours. The reaction mixture was then diluted with DCM (50 mL) and washed with 1 M HCl (3 x 20 mL), sat. $NaHCO_3$ (3 x 20 mL) and brine (20 mL), dried over $MgSO_4$, filtered and concentrated to give the crude mesylate (9.4 g, 56.9 mmol, 98% yield). To the crude mesylate (2.5 g, 15.2 mmol) at 0 ºC was added dimethylamine (8.6 mL, 76.1 mmol, 40% solution in $H_2O$) dropwise. The solution was allowed to warm to room temperature and stirred for 3 hours before being saturated with potassium carbonate and extracted with diethyl ether (5 x 20 mL). The combined organic extracts were dried over $MgSO_4$, filtered and purified by distillation (115 °C elution) to yield the title compound as a colourless liquid (1.5 g, 13.3 mmol, 88% yield). HRMS Calc. = 114.1283 $[M+H]^+$. Obs. = 114.1288 $[M+H]^+$. $^1$H NMR (400 MHz, $CDCl_3$) δ 4.77 (dd, J = 2.6, 1.2 Hz, 1H), 4.72 (dt, J = 2.1, 1.1 Hz, 1H), 2.43 (dd, J = 8.8, 6.7 Hz, 2H), 2.27 (s, 6H), 2.22 (t, J = 7.7 Hz, 2H), 1.76 (t, J = 1.1 Hz, 3H). $^{13}$C NMR (101 MHz, $CDCl_3$) δ 143.9 (C), 110.9 ($CH_2$), 58.0 ($CH_2$), 45.4 ($CH_3$), 35.8 ($CH_2$), 22.5 ($CH_3$). IR (ATR, $cm^{-1}$) - 3380brw, 3074w, 2970m, 2941m, 2815m, 2765m, 1649w, 1461s, 1306w, 1040m, 884s.

### ***N,N,N*-3-tetramethylbut-3-en-1-aminium iodide (11)**

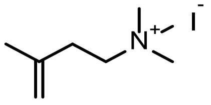

To *N,N*-3-trimethylbut-3-en-1-amine (**10**, 0.15 g, 1.33 mmol) in diethyl ether (25 mL) at 0 $^{o}$C was added methyl iodide (0.25 mL, 3.98 mmol) dropwise. The resulting solution was allowed to warm to room temperature and stir for 2 hours, where a white precipitate formed over time. The solution was filtered and the solid washed with diethyl ether. The white powder was then recrystallized from chloroform to produce the title compound as off-white needles (0.24 g, 0.941 mmol, 70% yield). HRMS Calc. = 128.1439 $[M]^+$. Obs. = 128.1452 $[M]^+$. $^1$H NMR (400 MHz, $CD_3OD$) δ 4.94 (t, J = 1.6 Hz, 1H), 4.90 (q, J = 1.1 Hz, 1H), 3.54 – 3.46 (m, 2H), 3.18 (s, 9H), 2.60 – 2.51 (m, 2H), 1.83 (t, J = 1.2 Hz, 3H). $^{13}$C NMR (101 MHz, $CD_3OD$) δ 120.1 (C), 114.2 ($CH_2$), 66.2 ($CH_2$), 53.7 ($CH_3$), 53.7 ($CH_3$) , 53.6 ($CH_3$), 31.8 ($CH_2$), 22.6 ($CH_3$). Melting point – 185-187 $^{o}$C. IR (ATR, cm$^{-1}$) - 3081w, 3005w, 2979w, 2947w, 1650w, 1477m, 1444w, 897s.

### ***N*-(3-methylbut-3-en-1-yl)-5-((3a*S*,4*S*,6a*R*)-2-oxohexahydro-1*H*-thieno[3,4-*d*]imidazol-4-yl)pentanamide (12)**

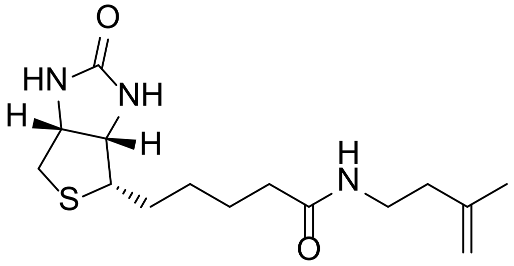

Biotin succinyl ester (982 mg, 2.94 mmol) was suspended in DMF (10 mL) and heated at 60 $^{o}$C until it dissolved. Triethylamine (818 µL, 5.88 mmol) and 3-methylbut-3-en-1-amine (250 µL, 2.94 mmol) were then added, and the resulting solution was stirred at room temperature for 16 h. The reaction mixture was then concentrated under reduced pressure. This was purified by column chromatography (10% methanol in dichloromethane) to give the title compound as an off-white solid (122 mg, 0.392 mmol, 14%). HRMS Calc.: 312.1740 $[M+H]^+$. Obs.: 312.1738 $[M+H]^+$. $^1$H NMR (400 MHz, DMSO-d6) δ 7.72 (t, *J* =

5.7 Hz, 1H), 6.42 (s, 1H), 6.35 (s, 1H), 4.70 (d, *J* = 23.0 Hz, 1H), 4.30 (dd, *J* = 7.7, 5.0 Hz, 1H), 4.12 (ddd, *J* = 7.2, 4.4, 1.8 Hz, 1H), 3.18 – 3.04 (m, 3H), 2.82 (dd, *J* = 12.4, 5.1 Hz, 1H), 2.57 (d, *J* = 12.6 Hz, 1H), 2.06 (dt, *J* = 22.8, 7.3 Hz, 4H), 1.68 (s, 3H), 1.63 – 1.58 (m, 1H), 1.53 – 1.40 (m, 3H), 1.36 – 1.22 (m, 2H). $^{13}$C NMR (101 MHz, DMSO-d6) δ 171.8 (C=O), 162.7 (C=O), 143.0 (C), 111.4 ($CH_2$), 61.0 (CH), 59.2 (CH), 55.4 (CH), 39.9 ($CH_2$), 37.3 ($CH_2$), 36.8 ($CH_2$), 35.2 ($CH_2$), 28.2 ($CH_2$), 28.1 ($CH_2$), 25.4 ($CH_2$), 22.2 ($CH_3$).

**_N,N_-2-trimethylprop-2-en-1-amine (13)**

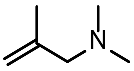


To dimethylamine (46.0 mL, 408 mmol, 40% solution in $H_2O$) at 0 °C was added 3-chloro-2-methylprop-1-ene (10.0 mL, 102 mmol) dropwise. After complete addition the reaction mixture was allowed to warm to room temperature and stir for 16 hours. The reaction mixture was saturated with potassium carbonate, then extracted with diethyl ether (5 x 20 mL). The combined organics were dried over $MgSO_4$, filtered and purified by distillation. The title compound eluted as a colourless liquid at 82 °C (6.21 g, 62.6 mmol, 61% yield). HRMS Calc. = 100.1126 $[M+H]^+$. Obs. = 100.1128 $[M+H]^+$. $^1$H NMR (400 MHz, $CDCl_3$) δ 4.91 – 4.84 (m, 2H), 2.84 (s, 2H), 2.22 (s, 6H), 1.78 (s, 2H). $^{13}$C NMR (101 MHz, $CDCl_3$) δ 143.1 (C), 113.3 ($CH_2$), 66.9 ($CH_2$), 45.3 ($CH_3$), 20.8 ($CH_3$). IR (ATR, $cm^{-1}$) – 3371brw, 3075w, 2969m, 2942m, 2858m, 2815m, 2765m, 1649m, 1537s, 1374m, 1097m, 884s.

**_N_-(2-methylallyl)-5-((3a*S*,4*S*,6a*R*)-2-oxohexahydro-1*H*-thieno[3,4-*d*]imidazol-4-yl)pentanamide (15)**

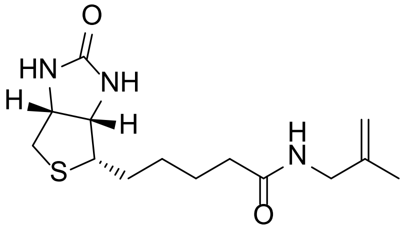


Biotin succinyl ester (1.11 g, 3.26 mmol) was suspended in DMF (10 mL) and heated at 60 °C until it dissolved. Triethylamine (906 µL, 6.51 mmol) and

methylallyl amine hydrochloride (418 mg, 3.91 mmol) were then added, and the resulting solution was stirred at room temperature for 16 h. The reaction mixture was then concentrated under reduced pressure. This was purified by column chromatography (10% methanol in dichloromethane) to give the title compound as a white solid (151 mg, 0.508 mmol, 16%). HRMS Calc.: 298.1584 $[M+H]^+$. Obs.: 298.1578 $[M+H]^+$. $^1$H NMR (400 MHz, DMSO-d6) δ 7.95 (t, *J* = 6.0 Hz, 1H), 6.42 (s, 1H), 6.35 (s, 1H), 4.86 – 4.65 (m, 2H), 4.30 (dd, *J* = 7.7, 5.0 Hz, 1H), 4.12 (ddd, *J* = 7.3, 4.5, 1.8 Hz, 1H), 3.59 (d, *J* = 5.9 Hz, 2H), 3.12-3.03 (m, 2H), 2.82 (dd, *J* = 12.4, 5.1 Hz, 1H), 2.57 (d, *J* = 12.6 Hz, 1H), 2.11 (t, *J* = 7.4 Hz, 2H), 1.65 (s, 3H), 1.55-1.45 (m, 2H), 1.31 (tt, *J* = 8.8, 4.3 Hz, 2H), 1.19 (t, *J* = 7.3 Hz, 2H). $^{13}$C NMR (101 MHz, DMSO-d6) δ 171.9 (C=O), 162.7 (C=O), 142.7 (C), 109.8 ($CH_2$), 61.1 (CH), 59.2 (CH), 55.4 (CH), 45.5 ($CH_2$), 43.8 ($CH_2$), 35.1 ($CH_2$), 28.3 ($CH_2$), 28.1 ($CH_2$), 25.4 ($CH_2$), 20.2 ($CH_3$).

**4-((3-methylbut-3-en-1-yl)amino)-4-oxobutanoic acid (16)**

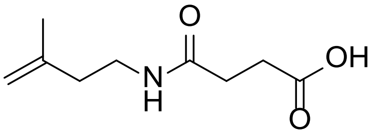


To succinic anhydride (94 mg, 0.941 mmol) in acetone (2 mL) was added 3-methyl-3-buten-1-amine (100 mg, 1.18 mmol) dropwise. The reaction mixture was stirred at room temperature for 4 hours then concentrated in vacuo to give a pale-yellow solid. This was purified by column chromatography (DCM:MeOH:AcOH 90:9:1) to yield the title compound as a pale yellow solid (159 mg, 0.859 mmol, 73%). HRMS Calc: 184.0979 $[M+H]^-$ ; Obs: 184.0391 $[M+H]^-$ . IR (ATR, $cm^{-1}$ ) 3303brm, 3072w, 2932w, 1633s, 1603s, 1544s, 1491w, 1446w. $^1$H NMR (400 MHz, DMSO) δ 12.02 (s, 1H), 7.78 (d, *J* = 5.8 Hz, 1H), 4.74 – 4.65 (m, 2H), 3.14 (td, *J* = 7.3, 5.6 Hz, 2H), 2.43 – 2.36 (m, 2H), 2.28 (t, *J* = 7.2 Hz, 2H), 2.08 (t, *J* = 7.3 Hz, 2H), 1.68 (s, 3H). $^{13}$C NMR (100 MHz, DMSO) δ 173.8 (C), 170.7 (C), 143.0 (C), 111.3 ($CH_2$), 37.2 ($CH_2$), 37.0 ($CH_2$), 30.0 ($CH_2$), 29.2 ($CH_2$), 22.3 ($CH_3$).

***N*-(3-methylbut-3-en-1-yl)benzamide (17)**

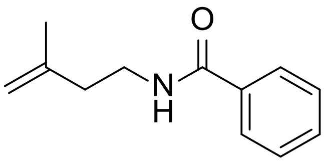

To 3-methyl-3-buten-1-amine (100 mg, 1.18 mmol) and triethylamine (491 µl, 3.53 mmol) in DCM (4 ml) at 0 °C was added benzoyl chloride (122 µl, 1.06 mmol) dropwise. The reaction mixture was allowed to warm to room temperature and stir for 16 hours before being diluted with DCM (25 ml) and washed with 1 M HCl (3 x 10 ml), sat. $NaHCO_3$ (3 x 10 ml) and brine (10 ml). The organic layer was then dried over $MgSO_4$, filtered and concentrated to product a pale-yellow oil. This was purified by column chromatography (1:3 EtOAc:pentane) to yield the title compound as a colourless oil (113 mg, 0.598 mmol, 51% yield). HRMS Calc: 190.1226 [M+H]+ ; Obs: 190.2095 [M+H]+ . IR (ATR, $cm^{-1}$) 3306s, 3081w, 2934brm, 1690s, 1643s, 1549s, 1426m. $^{1}H$ NMR (400 MHz, $CDCl_3$) δ 7.76 – 7.71 (m, 2H), 7.51 – 7.46 (m, 1H), 7.44 – 7.38 (m, 2H), 6.21 (s, 1H), 4.88 – 4.79 (m, 2H), 3.58 (td, *J* = 6.7, 5.4 Hz, 2H), 2.34 (td, *J* = 6.7, 1.2 Hz, 2H), 1.78 (t, *J* = 1.1 Hz, 3H). $^{13}C$ NMR (100 MHz, $CDCl_3$) δ 167.5 (C), 142.9 (C), 134.8 (C), 131.5 (CH), 128.7 (CH), 126.9 (CH), 112.7 ($CH_2$), 37.5 ($CH_2$), 37.4 ($CH_2$), 22.0 ($CH_3$).

## Peptide Synthesis

### Ac-CAY-NH$_2$ (4a)

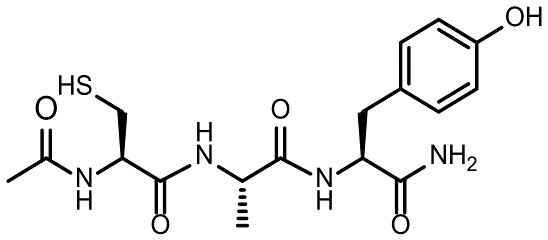


Peptide **4a** was synthesized using general automated synthesizer protocol with microwave assistance on rink amide resin (0.2 mmol). The crude peptide was purified by preparative RP-HPLC (5-60% B over 30 minutes) and lyophilized to produce the desired peptide (**4a**, 38 mg, 0.096 mmol, 48% yield).

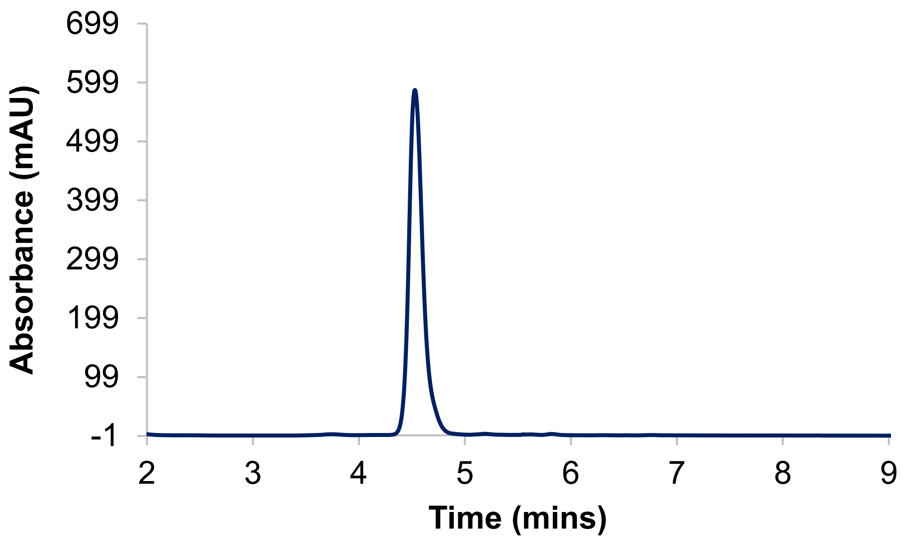


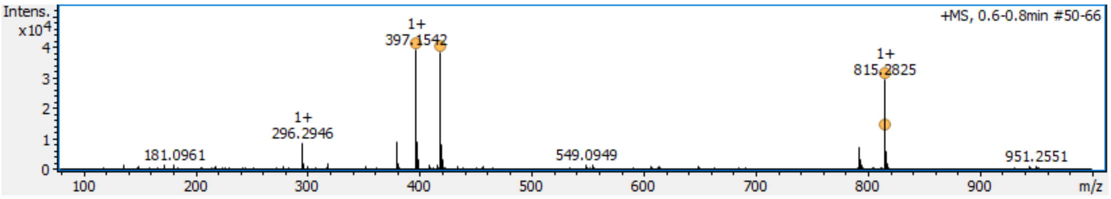


**Figure S2** - Analytical HPLC trace and ESI MS of purified Ac-CAY-NH$_2$ (**4a**). Analytical gradient 5-60% B over 10 min, 210 nm. Calculated Mass $[M+H]^+$: 397.15. Observed Mass $[M+H]^+$: 397.15.

**Ac-D-CAY-NH2 (4b)**

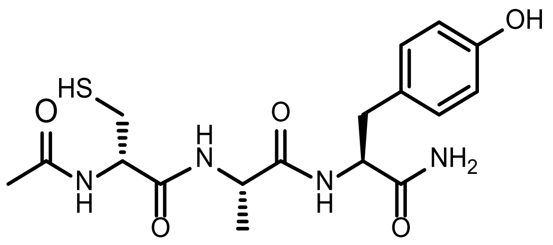


Peptide **4b** was synthesized using general automated synthesizer protocol with microwave assistance on rink amide resin (0.2 mmol). The crude peptide was purified by preparative RP-HPLC (5-60% B over 30 minutes) and lyophilized to produce the desired peptide (**4b**, 32 mg, 0.081 mmol, 41% yield).

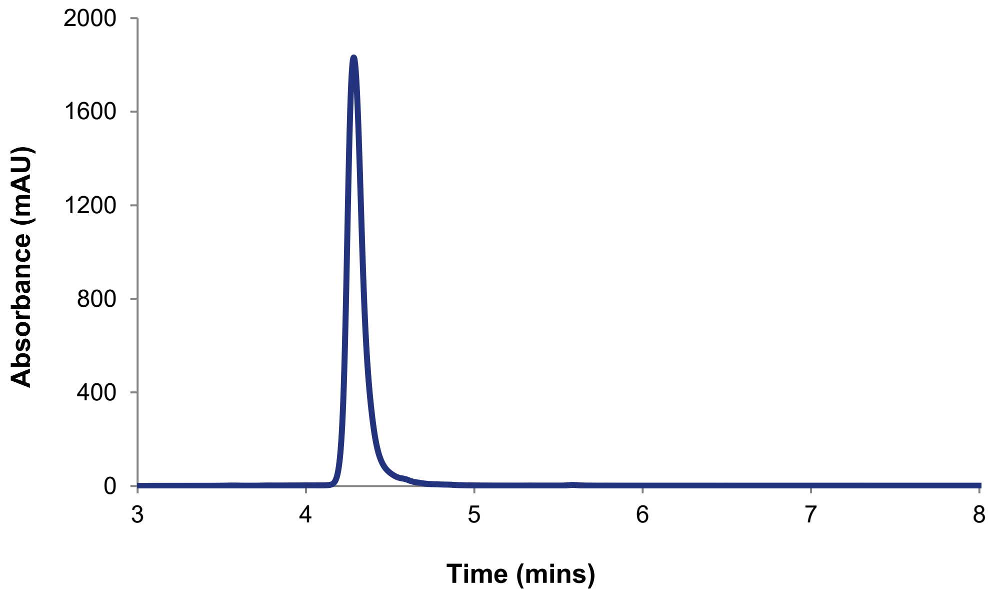


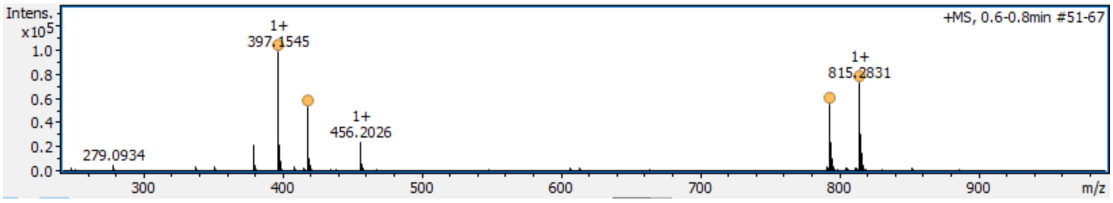


**Figure S3** - Analytical HPLC trace and ESI MS of purified Ac-D-CAY-NH2 (**4b**). Analytical gradient 5-60% B over 10 min, 210 nm. Calculated Mass $[M+H]^+$: 397.15. Observed Mass $[M+H]^+$: 397.15.

**Ac-CWHISKEY-NH2 (1)**

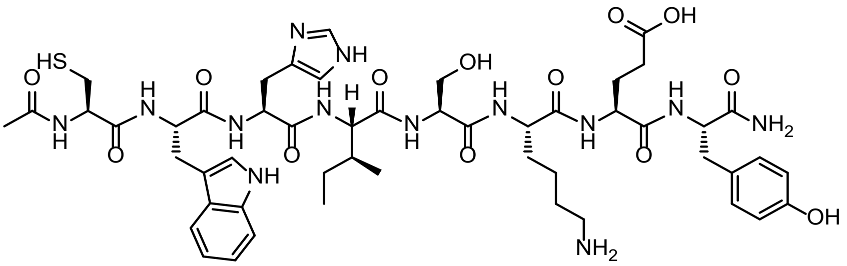


Peptide **1** was synthesized using general automated synthesizer procedure with microwave assistance on rink amide resin (0.2 mmol). The crude peptide was purified by preparative RP-HPLC (15-80% B over 30 minutes) and lyophilized to produce the desired peptide (**1**, 89 mg, 0.08 mmol, 40% yield).

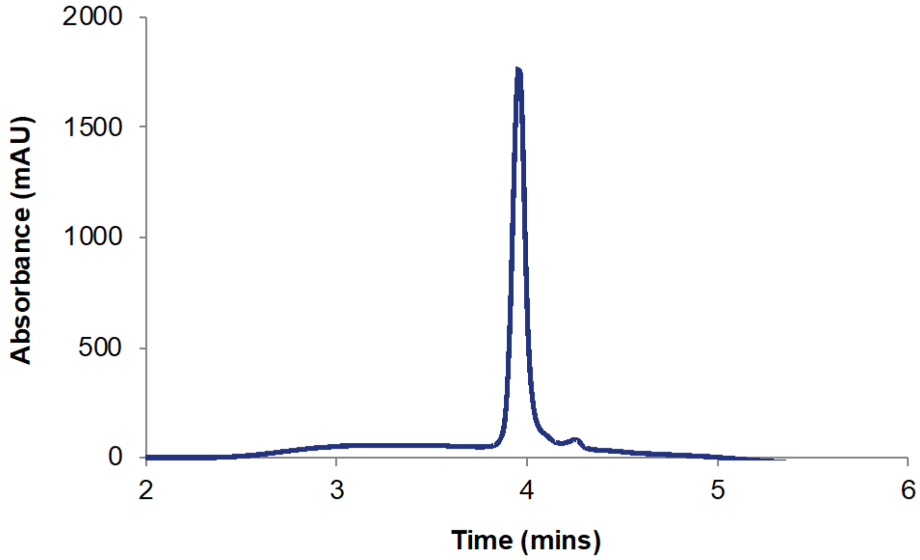


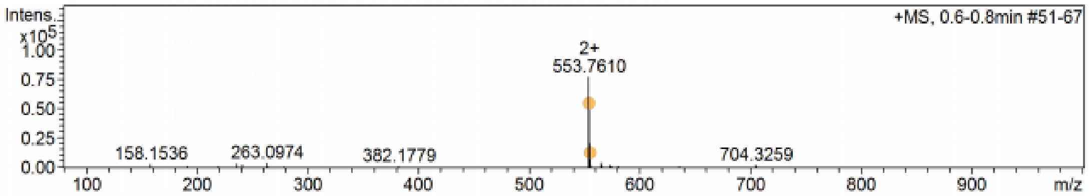


**Figure S4** - Analytical HPLC trace and ESI MS of purified Ac-CWHISKEY-NH2 (**1**). Analytical gradient 10-100% B over 5 min, 210 nm. Calculated Mass $[M+2H]^{2+}$: 553.76. Observed Mass $[M+2H]^{2+}$: 553.76.

## Synthesis of Ac-MACY-$NH_2$ (6)

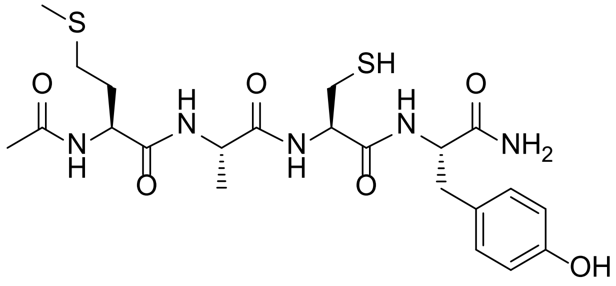

Model peptide **6** was synthesized using general automated synthesizer procedure with microwave assistance on rink amide resin (0.25 mmol). The crude peptide was purified by preparative RP-HPLC (2-95% B over 30 minutes) and lyophilized to produce the desired peptide (64 mg, 0.12 mmol, 49% yield).

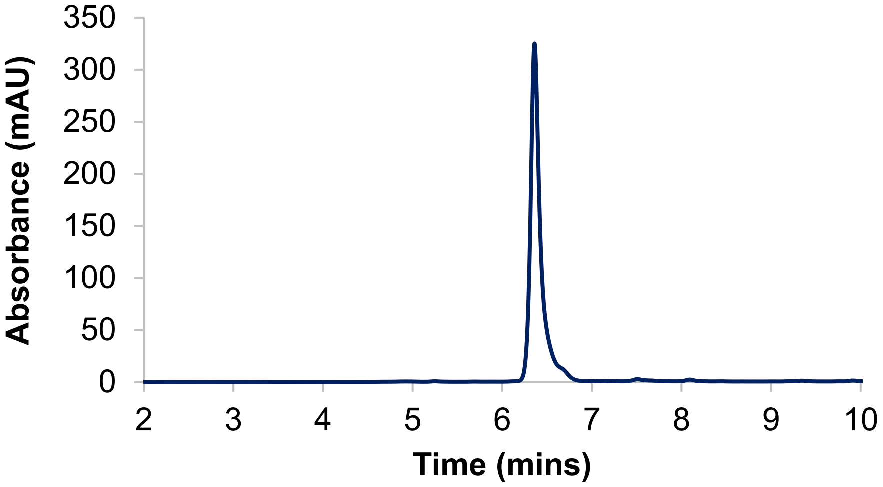


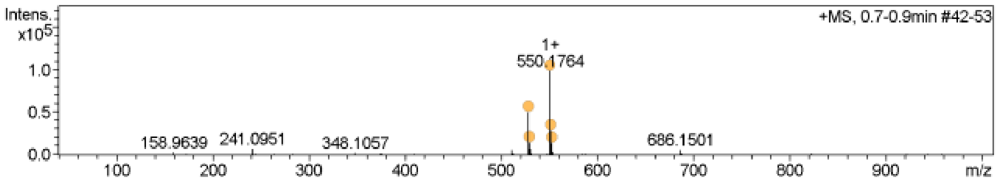


**Figure S5** - Analytical HPLC trace and ESI MS for purified Ac-MACY-$NH_2$ (**6**); analytical gradient 5-60% B over 10 min, 280 nm. Calculated mass $[M+Na]^+$: 550.18; observed mass $[M+Na]^+$: 550.18.

**Ac-YEPLACHISKY-NH2 (8)**

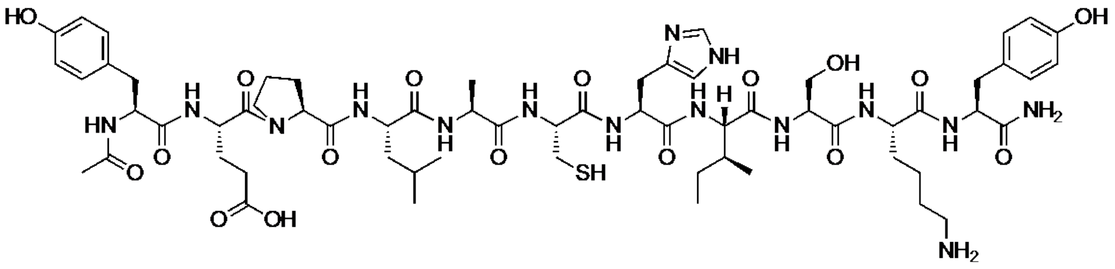

Peptide **8** was synthesized using general automated synthesizer procedure with microwave assistance on rink amide resin (0.2 mmol). The crude peptide was purified by preparative RP-HPLC (15-80% B over 30 minutes) and lyophilized to produce the desired peptide (**6**, 44 mg, 0.032 mmol, 16% yield).

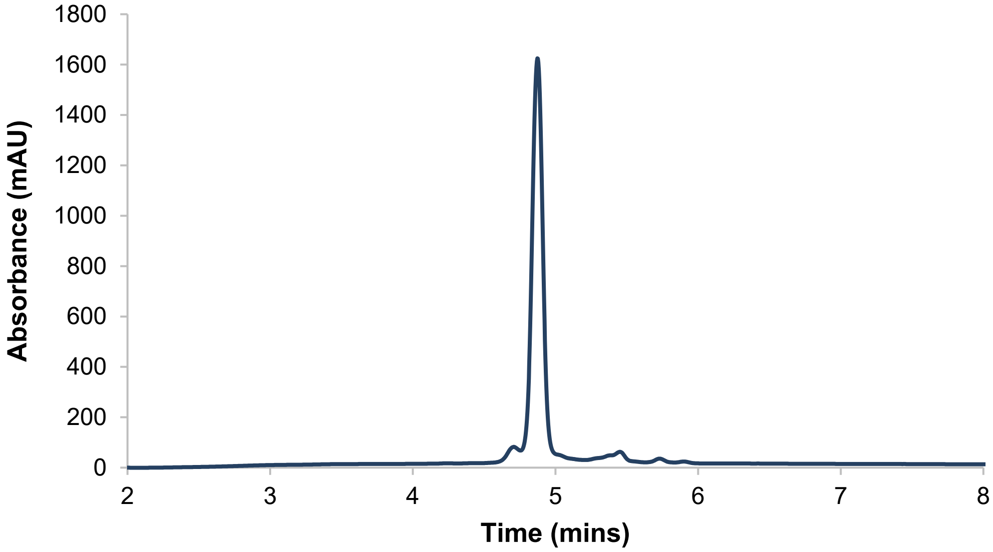


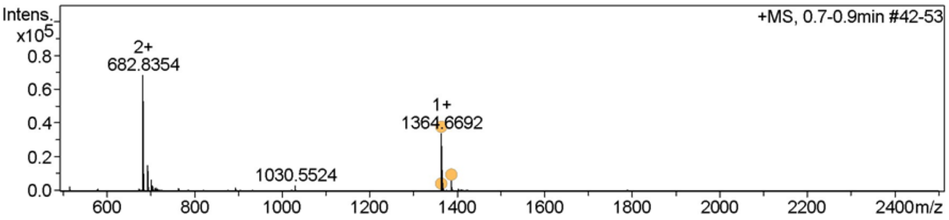


**Figure S6** - Analytical HPLC trace and ESI MS of purified Ac-YEPLACHISKY-NH2 (**6**). Analytical gradient 15-60% B over 10 min, 210 nm. Calculated Mass $[M+H]^{2+}$: 1364.67. Observed Mass $[M+H]^{2+}$: 1364.67

**Ac-YECPLAHISCKY-$NH_2$ (26)**

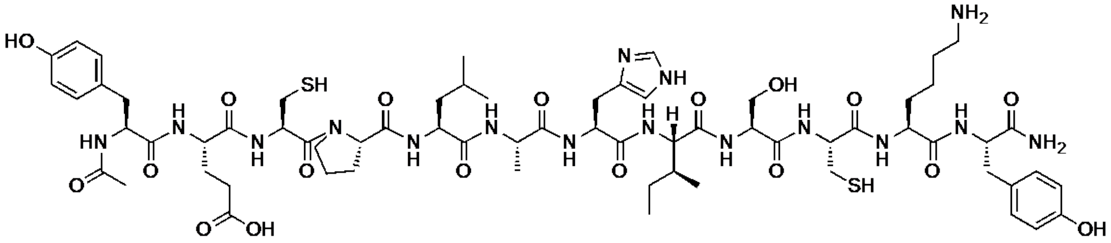

Peptide **26** was synthesized using general automated synthesizer protocol with microwave assistance on rink amide resin (0.2 mmol). The crude peptide was purified by preparative RP-HPLC (15-80% B over 30 minutes) and lyophilized to produce the desired peptide (**26**, 76.2 mg, 0.05 mmol, 52% yield).

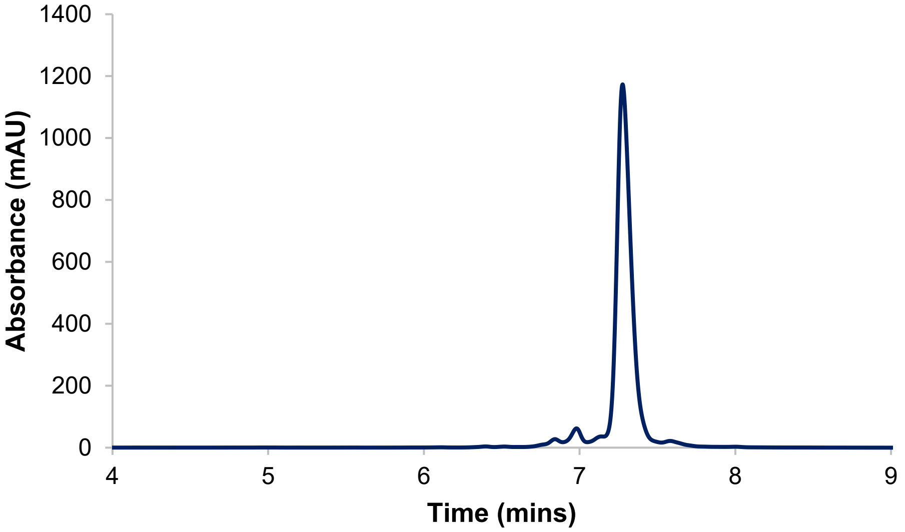


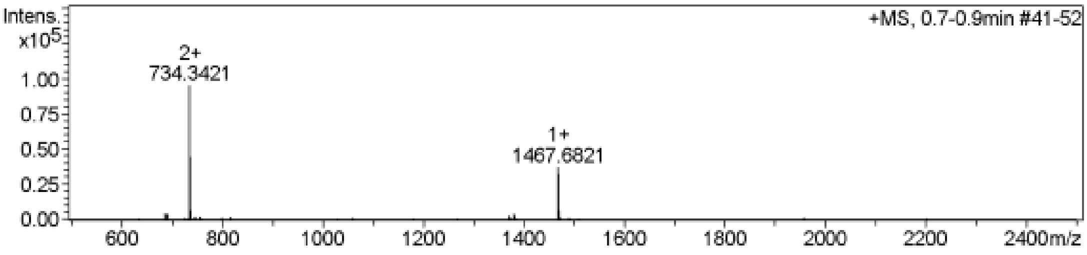


**Figure S7** - Analytical HPLC trace and ESI MS of purified Ac-YECPLAHISCKEY-$NH_2$ (**26**). Analytical gradient 5-60% B over 10 min, 280 nm. Calculated Mass $[M+H]^+$: 1467.67; Observed Mass $[M+H]^+$: 1467.68.

## Optimization of visible-light-mediated conjugation

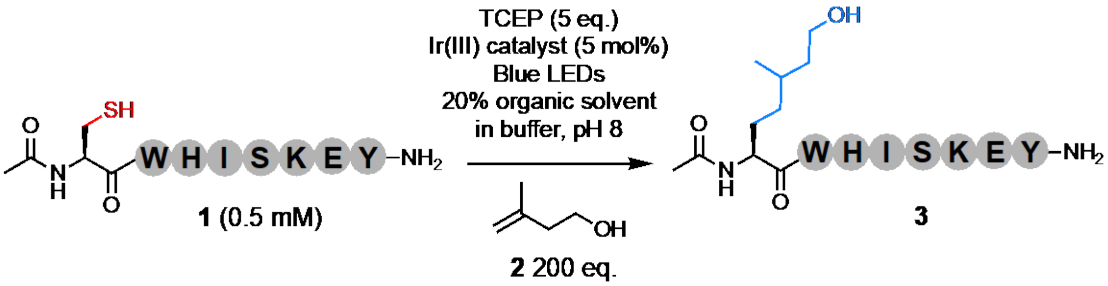


Product **3** was synthesised using protocol A conditions with Ac-CWHISKEY-$NH_2$ (**1**, 2 mg, 1.81 μmol) and isoprenol (**2**, 31.2 mg, 0.362 mmol); after 60 mins, the conversion to product was observed to be 83% (determined via analytical HPLC) with 17% conversion to the Ala by-product. After analysis the remainder of material (1.745 μmol) was purified using semi- preparative HPLC (10-70% B over 30 minutes) to yield the desired conjugate (**3**, 1.6 mg, 1.38 μmol, 79% yield).

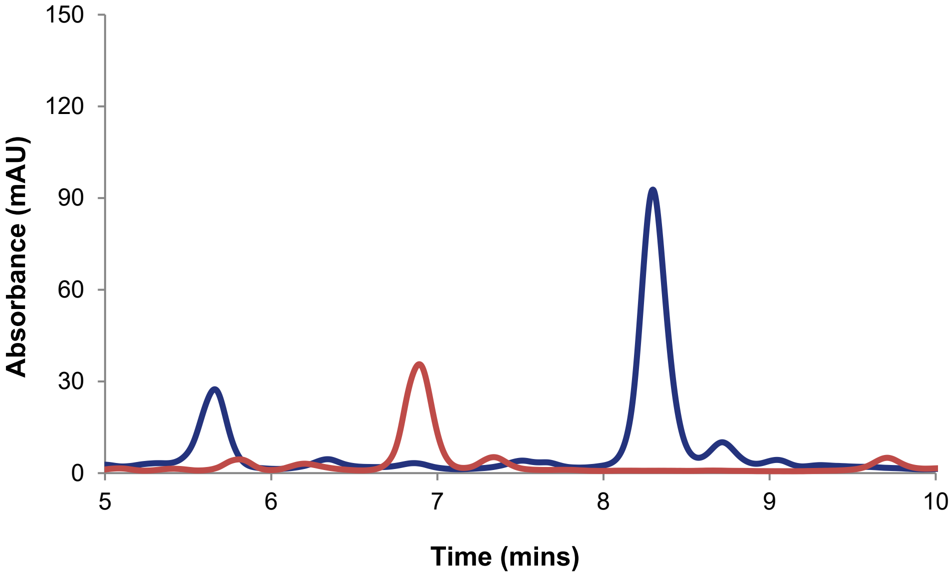


**Figure S8** - Crude HPLC trace for the modification of Ac-CWHISKEY-$NH_2$ (**1**) with 3-methyl-3-buten-1-ol (isoprenol, **2**); analytical gradient 10-70% B over 10 min, 280 nm. Pure SM in red and crude trace in blue. Peak at 5.5 minutes is the Ala by-product, the desired product elutes at 8.2 min.

**Table S1** - Optimisation data showing initiator compatibility using 0.5 mM peptide, 5 eq. TCEP and 200 eq. isoprenol (**2**) in 20% DMSO in CB at pH 7.5-8.5 for 1 hour. Ir and EY reactions performed in blue LEDs, Mn reaction at 50 °C.

| Trial | Initiator | Eq. | Product %conv. | Ala %conv. | SM %rem. |
|---|---|---|---|---|---|
| 1 | Ir | 0.05 | 83 | 17 | 0 |
| 2 | Eosin Y | 0.1 | 73 | 27 | 0 |
| 3 | $Mn(OAc)_3$ | 5 | 49 | 21 | 30 |

**Table S2**- Optimisation data for TCEP and alkene eq. Conditions used were 0.5 mM peptide and 5 mol% Ir in 20% DMSO in CB at pH 8 for 1 hour.

| Trial | Isoprenol (**2**) eq. | TCEP eq. | Product %conv. | Ala %conv. |
|---|---|---|---|---|
| 4 | 200 | 5 | 83 | 17 |
| 5 | 100 | 5 | 61 | 39 |
| 6 | 50 | 5 | 51 | 49 |
| 7 | 20 | 5 | 30 | 70 |
| 8 | 100 | 50 | 46 | 54 |
| 9 | 50 | 50 | 58 | 42 |
| 10 | 20 | 50 | 19 | 81 |

Note: trial 4 was repeated with 10% DMSO; No reduction in conversion was observed.

**Table S3** - Organic co-solvent compatibility was tested to offer alternatives to DMSO. The reactions were performed on Ac-CWHISKEY-$NH_2$ at 0.5 mM with 5 eq. TCEP, 200 eq. isoprenol (**2**), 5 mol% Ir(III) in CB at pH 8 with the final solvent composition consisting of 10% of the specified solvent. Reactions were monitored after 1 hour.

| Trial | Solvent | Product %conv. | Ala %conv. |
|---|---|---|---|
| 11 | Methanol | 84 | 16 |
| 12 | Acetonitrile | 85 | 15 |

**Table S4** – Low concentration trials on Ac-CWHISKEY-$NH_2$ to assess protein applicability. Reactions use 0.05 mM Ac-CWHISKEY-$NH_2$ and isoprenol (**2**) in 10% DMSO in CB at pH 8. Values given in brackets is the percentage of starting material remaining.

| Trial | TCEP equiv. | Alkene equiv. | Ir equiv. | 1 hour %conv. | 2 hours %conv. | 3 hours % conv. |
|---|---|---|---|---|---|---|
| 13 | 5 | 200 | 0.05 | 0 (100) | 0 (100) | 0 (100) |
| 14 | 25 | 500 | 0.05 | 10(86) | 72 (10) | 77 (6) |
| 15 | 25 | 500 | 0.1 | 66 (19) | 82 (0) | 82(0) |
| 16 | 25 | 1000 | 0.1 | 77 (11) | 86 (0) | 86 (0) |
| 17 | 100 | 1000 | 0.1 | 87 (0) | 87 (0) | 87 (0) |

**Table S5** - Low concentration studies using Ac-YEPLACHISKY-$NH_2$ with 100 eq. TCEP, 1000 eq. isoprenol (**2**) and 10 mol% Ir in 10% DMSO in CB at pH 8. Values given in brackets is the percentage of starting material remaining.

| Trial | Peptide μM | 1 hour %conv |
|---|---|---|
| 18 | 50 | 82 (0) |
| 19 | 25 | 75 (8) |
| 20 | 10 | 25(57) |
| 21 | 5 | 0 (100) |
| 22 | 1 | 0 (100) |

## Progress of the reaction in the absence of blue light

Model peptide **1** (Ac-CWHISKEY-$NH_2$) was treated with the standard conjugation conditions described in Table 1, Entry 2 of the manuscript. Analytical HPLC analysis was performed prior to irradiation with blue light at t = 0 and 60 mins to demonstrate that the reaction only proceeds under blue light. The reaction was then irradiated for 2 mins to allow the reaction to partially proceed before being removed from the light and analysed by HPLC. The sample was left on the bench for 120 mins before further analysis to demonstrate that the reaction stalls completely when not irradiated.

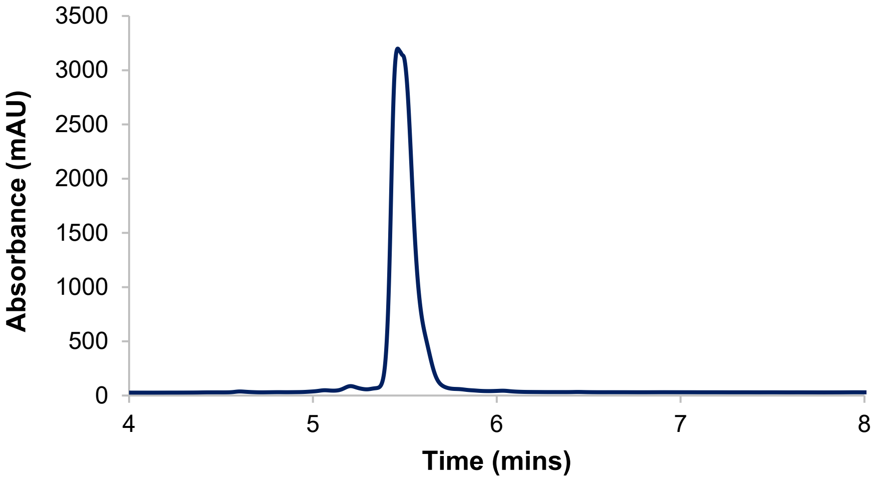


**Figure S9 -** Analytical HPLC trace of peptide **1** under C-C bond forming conditions in the dark, t = 0 mins; analytical gradient 10-70% B over 10 min, 280 nm.

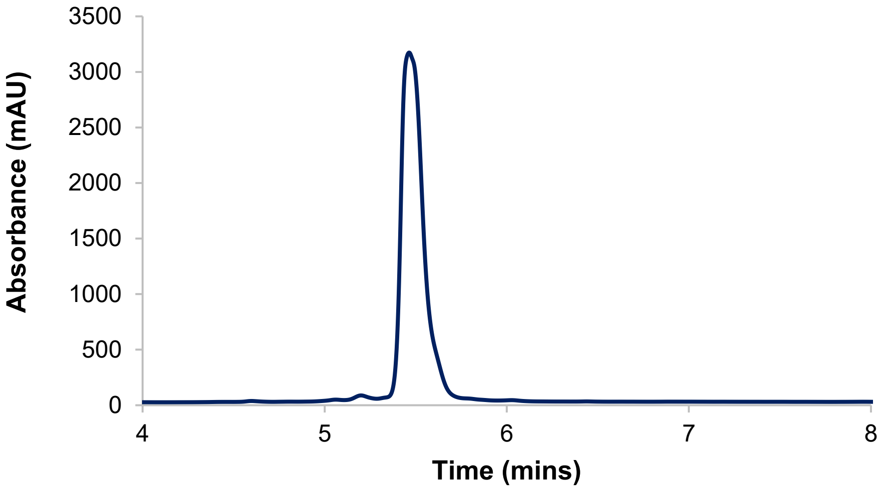


**Figure S10 -** Analytical HPLC trace of peptide **1** under C-C bond forming conditions in the dark, t = 60 mins; analytical gradient 10-70% B over 10 min, 280 nm.

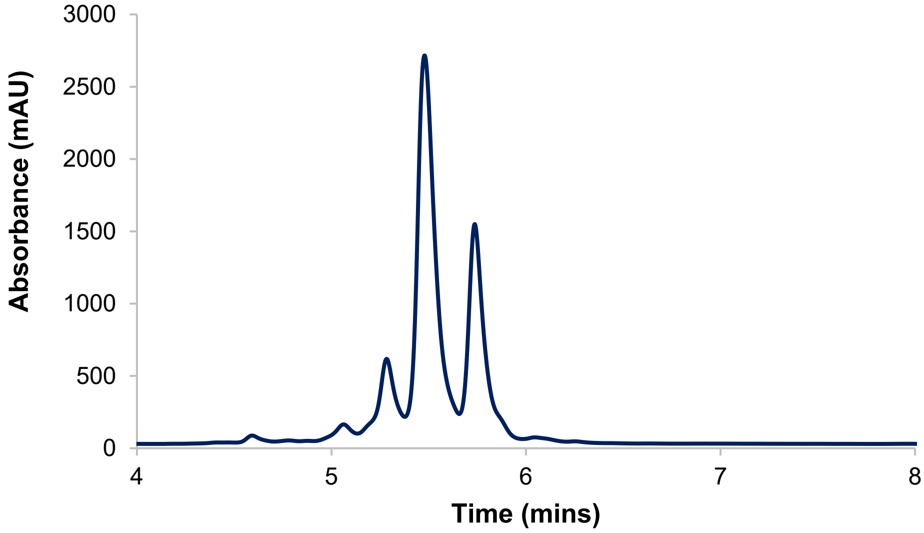


**Figure S11 -** Analytical HPLC trace of peptide **1** under C-C bond forming conditions under blue light, t = 2 mins; analytical gradient 10-70% B over 10 min, 280 nm.

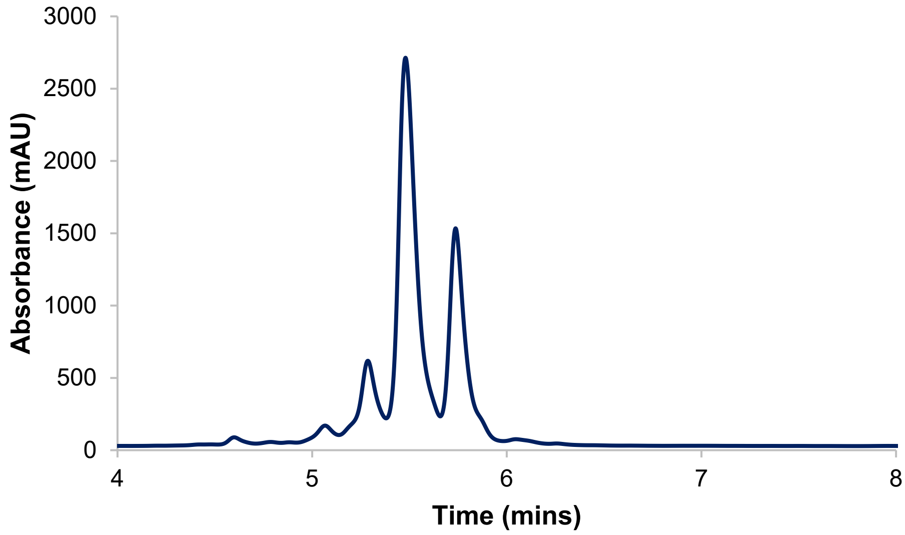


**Figure S12 -** Analytical HPLC trace of peptide **1** under C-C bond forming conditions under blue light for 2 mins and then stored in the dark for 120 mins prior to analysis; analytical gradient 10-70% B over 10 min, 280 nm.

## NMR analysis of conjugation

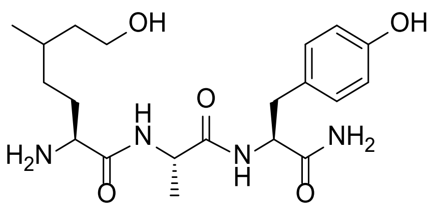

Product **5a** was synthesized following general protocol A using Ac-CAY-$NH_2$ (**4a**) (4.0 mg, 10.1 µmol) and isoprenol (**2**, 174 mg, 2.02 mmol), followed by purification using semi-preparative RP-HPLC (15-80% B over 30 minutes) and lyophilisation to produce the desired conjugated peptide **5a** (3.5 mg, 7.80 µmol, 77% yield). ESI MS Calc.: 251.26 $[M+H]^+$; obs.: 251.26 $[M+H]^+$. $^1H$ NMR (500 MHz, MeOD) δ 7.08 (d, J = 8.4 Hz, 2H), 6.71 (d, J = 8.4 Hz, 2H), 4.49 (dd, J = 8.6, 5.8 Hz, 1H), 4.25 (qd, J = 7.2, 1.4 Hz, 1H), 4.20 (ddd, J = 8.3, 5.6, 2.5 Hz, 1H), 3.67 – 3.56 (m, 2H), 3.08 (dd, J = 14.0, 5.7 Hz, 1H), 2.92 (ddd, J = 14.0, 8.6, 1.8 Hz, 1H), 2.03 (d, J = 0.7 Hz, 3H), 1.87 – 1.55 (m, 4H), 1.49 – 1.32 (m, 2H), 1.29 (d, J = 7.2 Hz, 3H), 1.26 – 1.14 (m, 1H), 0.93 (dd, 3H). $^{13}C$ NMR (126 MHz, MeOD) δ 174.7 (C), 173.5 (C), 173.3 (C), 172.3 (C), 155.9 (Ar), 129.9 (Ar), 127.7 (Ar-H), 114.8 (Ar-H), 59.5 (CH), 54.6 (CH), 54.3 (CH), 49.5 (CH), 39.2 ($CH_2$), 39.0 ($CH_2$), 36.4 ($CH_2$), 32.7 ($CH_2$), 29.1 ($CH_3$),29.0 ($CH_2$), 21.1 ($CH_2$), 18.5 ($CH_3$), 18.4 ($CH_2$) 16.2 ($CH_3$).

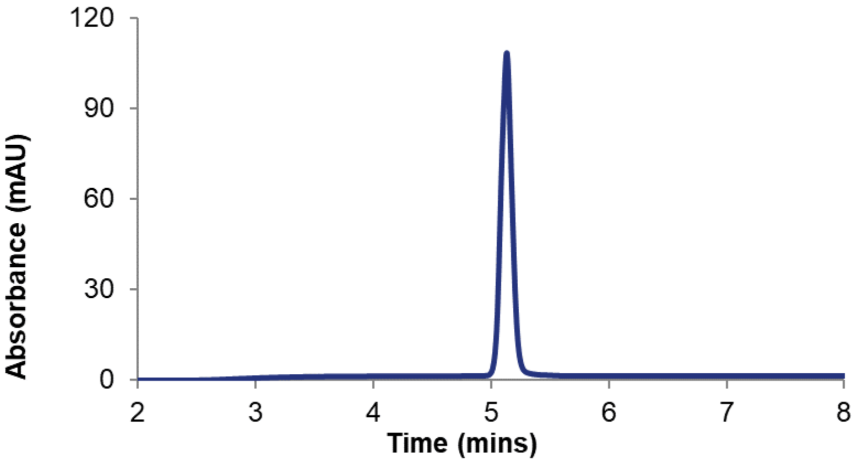

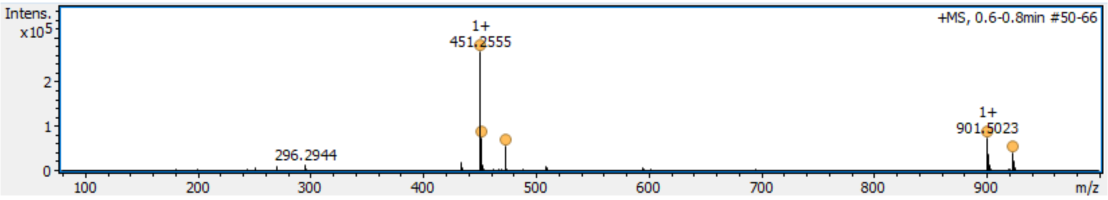


**Figure S13 -** Analytical HPLC trace and ESI MS of product **5a**; analytical gradient 5-60% B over 10 min, 280 nm. Calculated mass $[M+H]^+$: 451.26; observed mass $[M+H]^+$: 451.26.

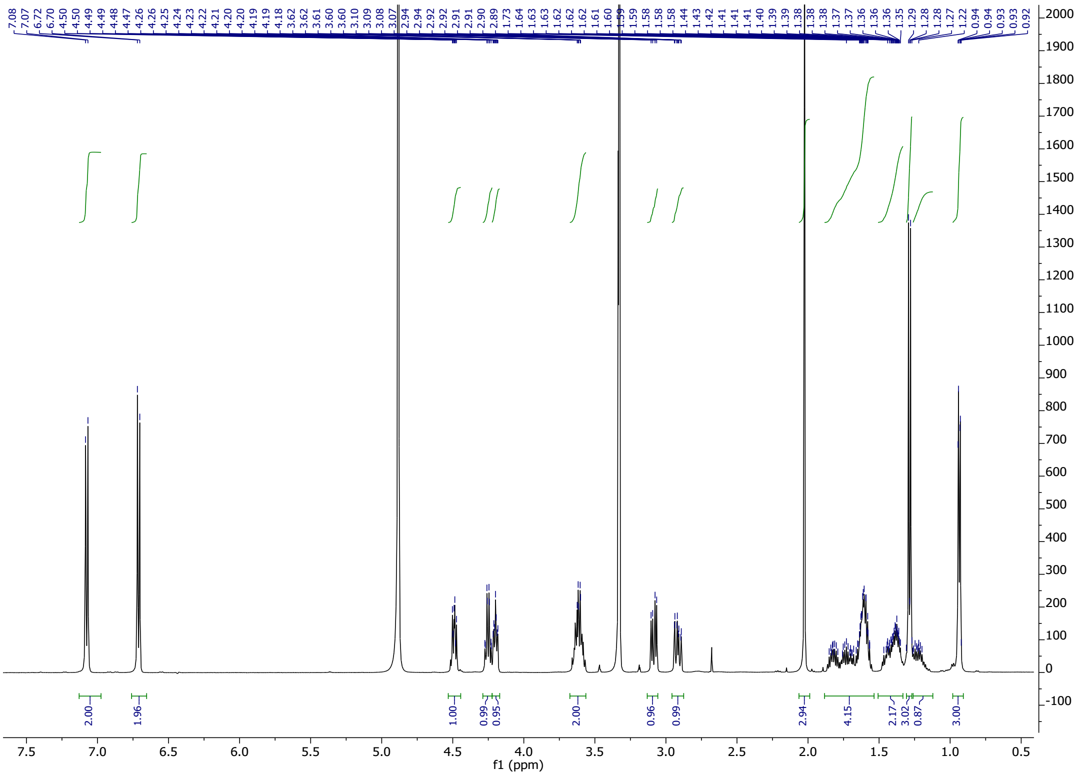


**Figure S14** – $^1$H NMR for product **5a**.

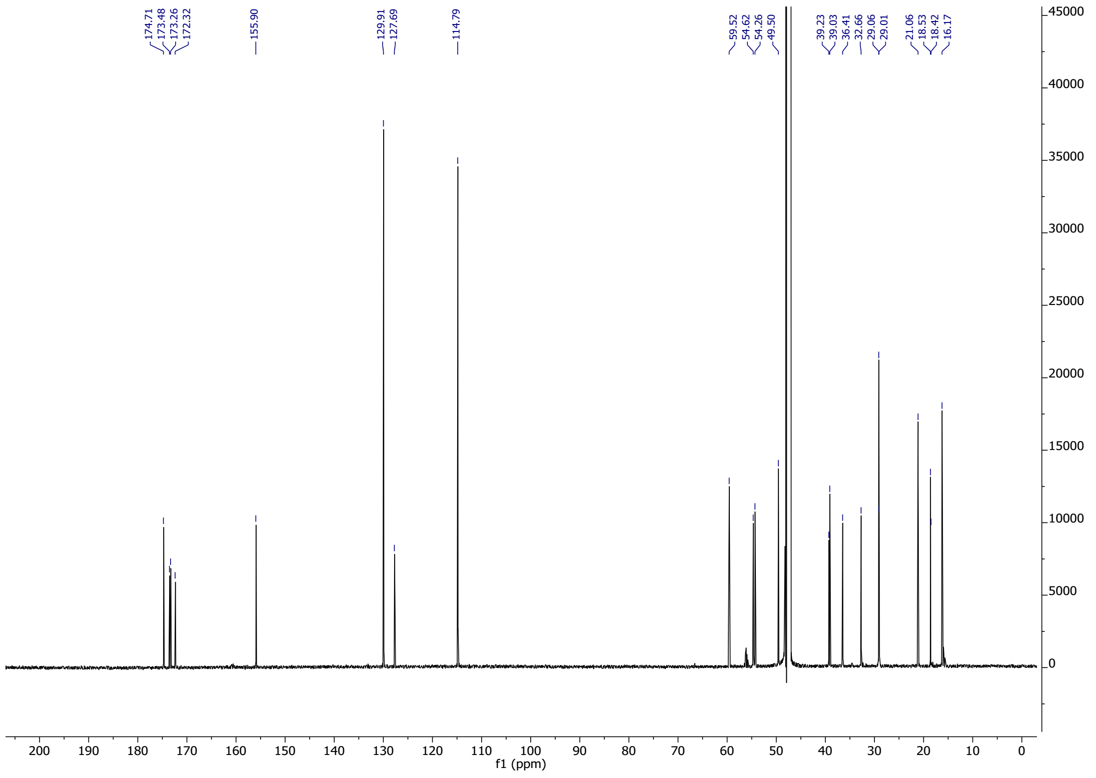

**Figure S15** – $^{13}$C NMR for product **5a**.

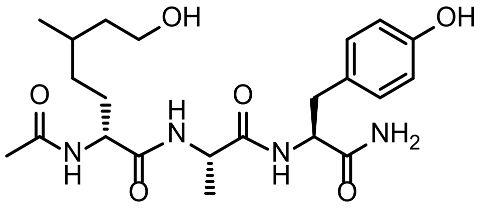

Product **5b** was synthesized following general protocol A using Ac-D-CAY-$NH_2$ (**4b**) (4.0 mg, 10.1 µmol) and isoprenol (**2**, 174 mg, 2.02 mmol), followed by purification using semi-preparative RP-HPLC (15-80% B over 30 minutes) and lyophilisation to produce the desired conjugated peptide **5b** (3.3 mg, 8.08 µmol, 80% yield). ESI MS Calc.: 251.26 $[M+H]^+$; obs.: 251.26 $[M+H]^+$. $^1$H NMR (500 MHz, Methanol-d4) δ 7.97 – 7.89 (m, 1H), 7.17 – 7.10 (m, 2H), 6.71 (d, J = 8.5 Hz, 2H), 4.49 – 4.40 (m, 1H), 4.10 (tt, J = 7.6, 2.3 Hz, 2H), 3.70 – 3.54 (m, 2H), 3.21 (ddd, J = 14.1, 4.3, 1.7 Hz, 1H), 2.94 (dd, J = 14.0, 11.2 Hz, 1H), 2.68 (s, 1H), 2.00 (s, 3H), 1.83 – 1.68 (m, 1H), 1.68 – 1.54 (m, 2H), 1.53 – 1.45 (m, 1H), 1.42 – 1.28 (m, 2H), 1.20 (d, J = 7.3 Hz, 3H), 0.94 (dd, J = 6.5, 3.6 Hz, 3H). $^{13}$C NMR (126 MHz, MeOD) δ 175.4 (C), 175.4 (C), 173.7 (C), 172.5 (C), 155.8

(Ar), 129.8 (Ar), 128.4 (Ar-H), 114.7 (Ar-H), 59.5 (CH), 59.4 (CH), 55.1 (CH), 54.8 (CH), 50.3 ($CH_2$), 39.1 ($CH_2$), 35.8 ($CH_2$), 32.7 ($CH_2$), 32.6 ($CH_2$), 29.1 ($CH_2$), 29.0 ($CH_3$), 28.1 ($CH_2$), 20.9 ($CH_2$), 18.4 ($CH_3$), 15.6 ($CH_3$).

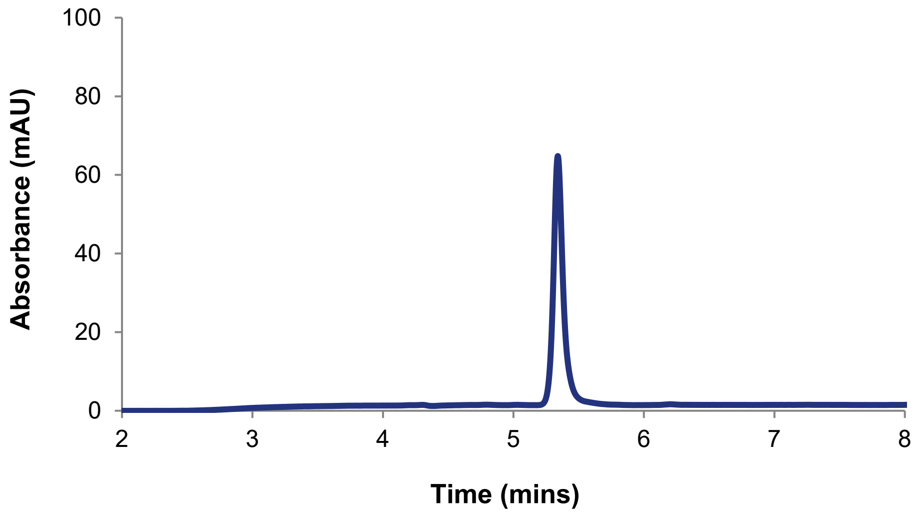


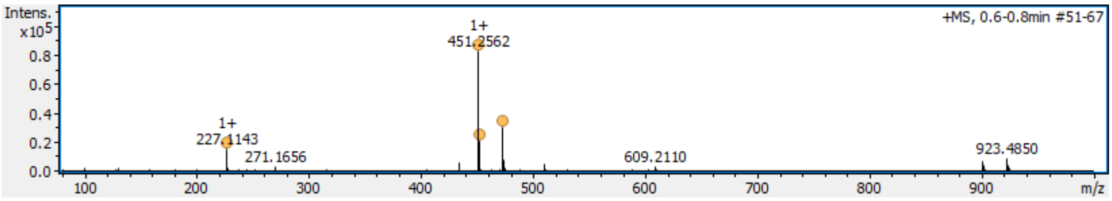


**Figure S16 -** Analytical HPLC trace and ESI MS of product **5b**; analytical gradient 5-60% B over 10 min, 280 nm. Calculated mass $[M+H]^+$: 451.26; observed mass $[M+H]^+$: 451.26.

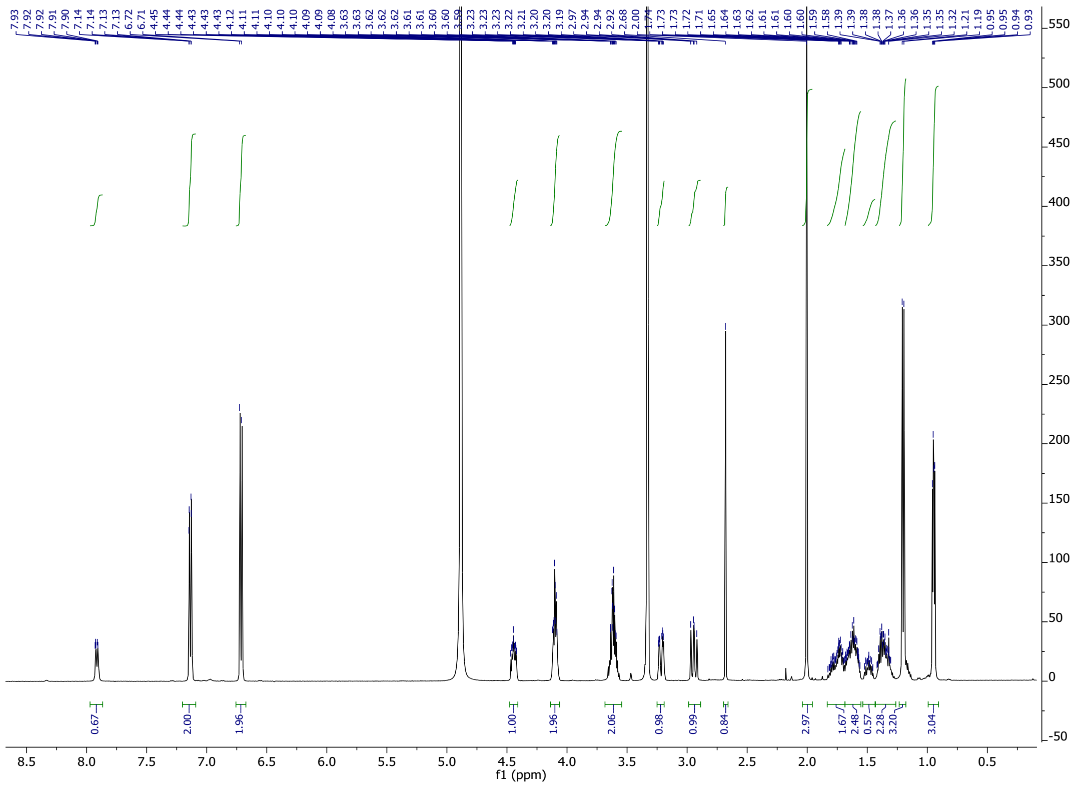

**Figure S17 -** $^{1}$H NMR for product **5b**.

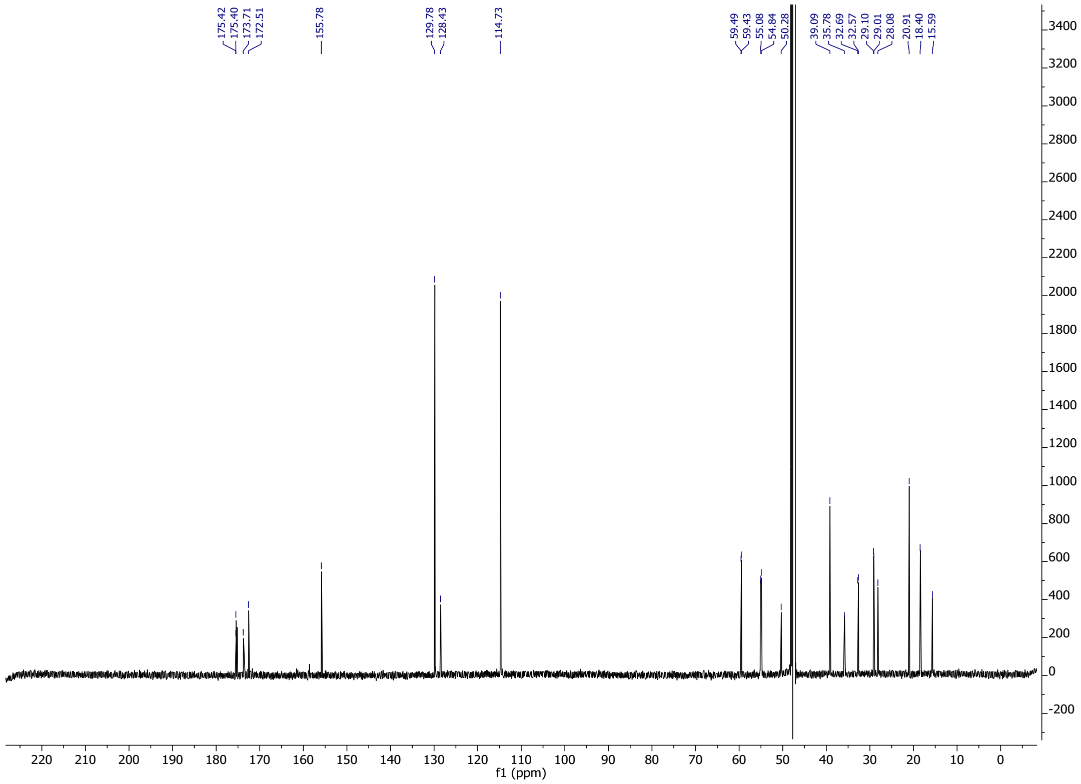

**Figure S18 –** $^{13}$C NMR for product **5b**.

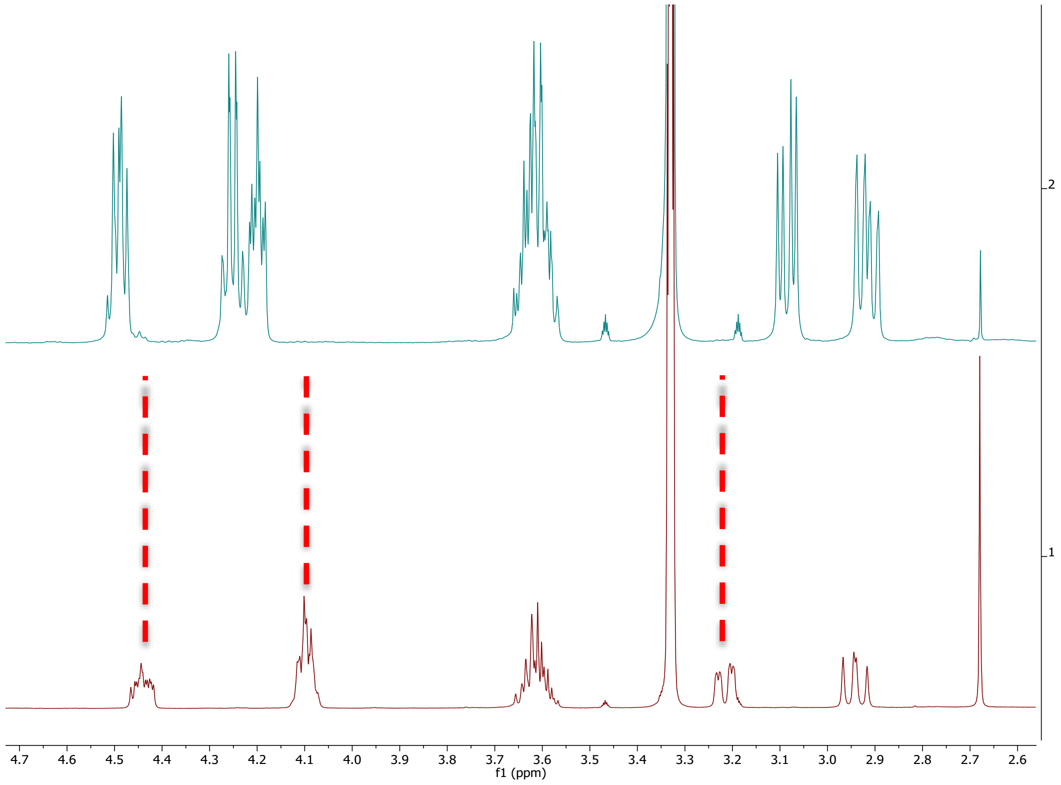


**Figure S19** - NMR comparison between products **5a** (L-stereochemistry, green) and **5b** (D-stereochemistry, red).

## Exploration of reaction scope

### Ac-CWHISKEY-NH2 (1) modified with isoprenol (2)

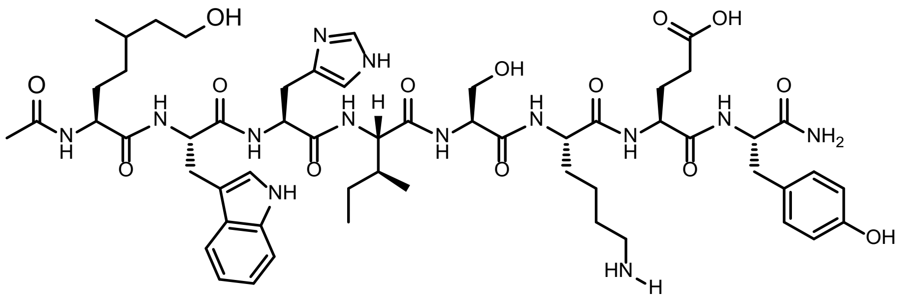


Product **3** was synthesized following general conjugation protocol A over 60 mins* using Ac-CWHISKEY-NH2 (**1**, 2 mg, 1.81 µmol) and isoprenol (**2**, 31 mg, 0.36 mmol). After analysis, the remainder of material (1.745 µmol) was purified using semi-preparative HPLC (10-70% B over 30 minutes) to yield the desired conjugated peptide (**3**, 1.6 mg, 1.38 µmol, 79% yield).

*Reaction observed to reach completion in under 10 minutes.

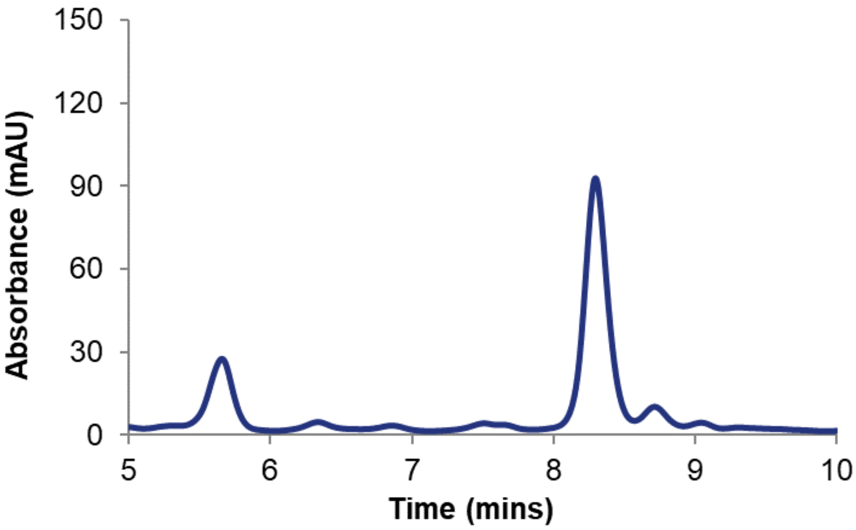


**Figure S20** - Analytical HPLC trace (crude reaction mixture) of Ac-CWHISKEY-NH2 (**1**) reacted with 3-methyl-3-buten-1-ol (**2**) after 1 hour. Analytical gradient 15-25% B over 10 min, 210 nm.

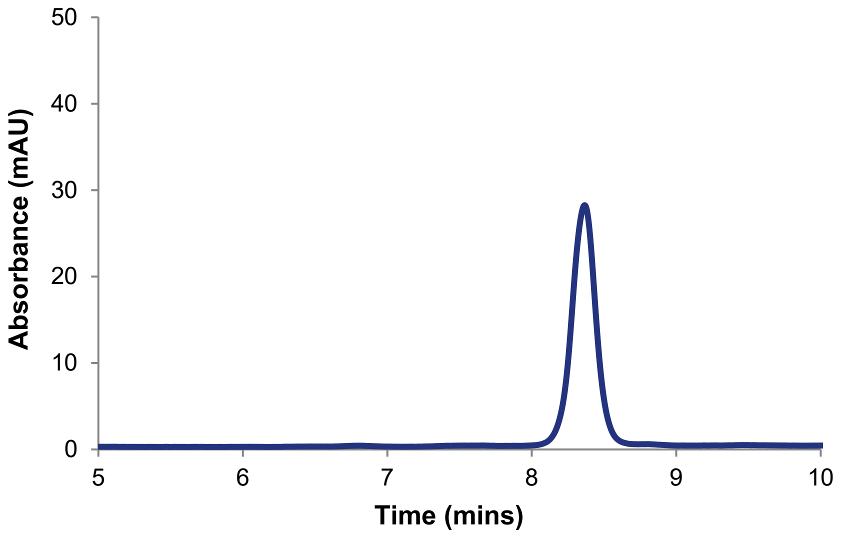


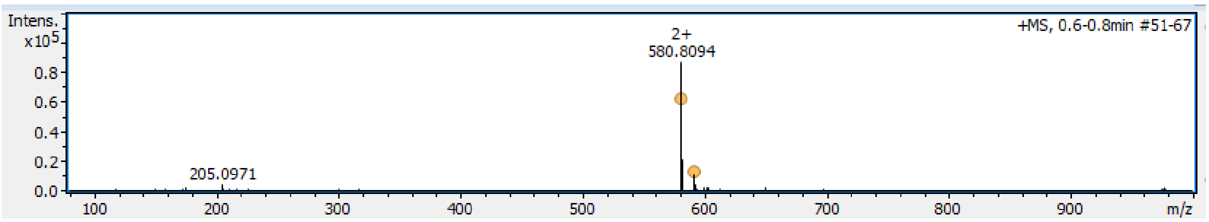


**Figure S21** - Analytical HPLC trace and ESI MS for purified product **3**; analytical gradient 10-70% B over 10 min, 280 nm. Calculated mass $[M+2H]^{2+}$: 580.80; observed mass $[M+2H]^{2+}$: 580.81.

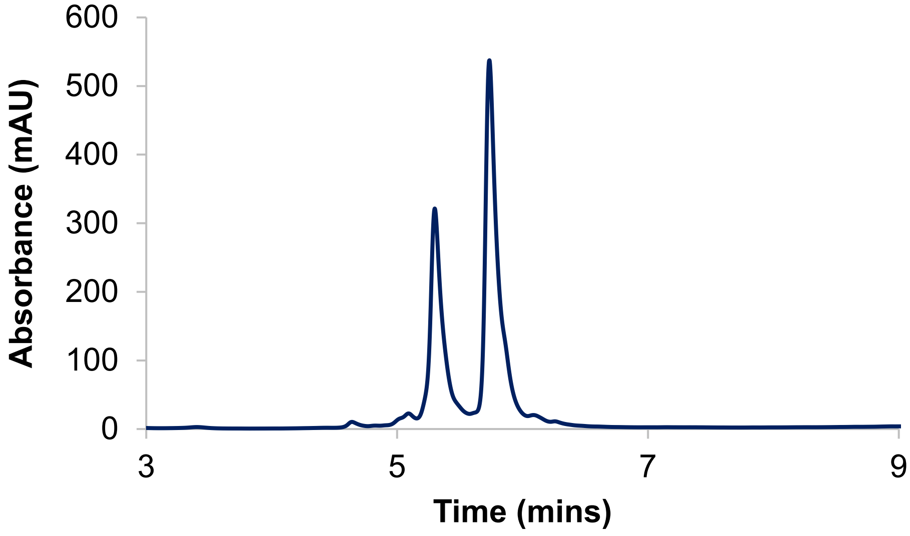


**Figure S22** - Analytical HPLC trace (crude reaction mixture) of Ac-CWHISKEY-$NH_2$ (**1**) reacted with 3-methyl-3-buten-1-ol (**2**) under protocol A conditions after 10 mins in phosphate buffered saline (PBS) in place of conjugation buffer (CB). Analytical gradient 10-70% B over 10 min, 280 nm. Ratio of product **3** : Ala by-product - 63:37.

**Ac-MACY-NH2 (6) modified with 3-methyl-3-buten-1-ol (2)**

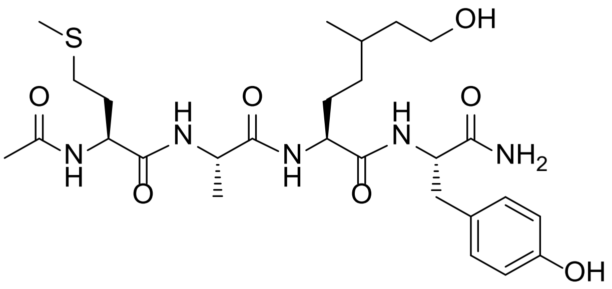


Product **7** was synthesised following the general protocol B over 30 mins using Ac-MACY-NH2 (**6**, 2 mg, 3.80 μmol) and compound **2** (77 mg, 0.76 mmol). After analysis the remainder of material (3.75 μmol) was purified using semi-preparative HPLC (5-60% B over 30 minutes) to yield the desired conjugated peptide (**7**, 1.5 mg, 2.58 μmol, 69% yield).

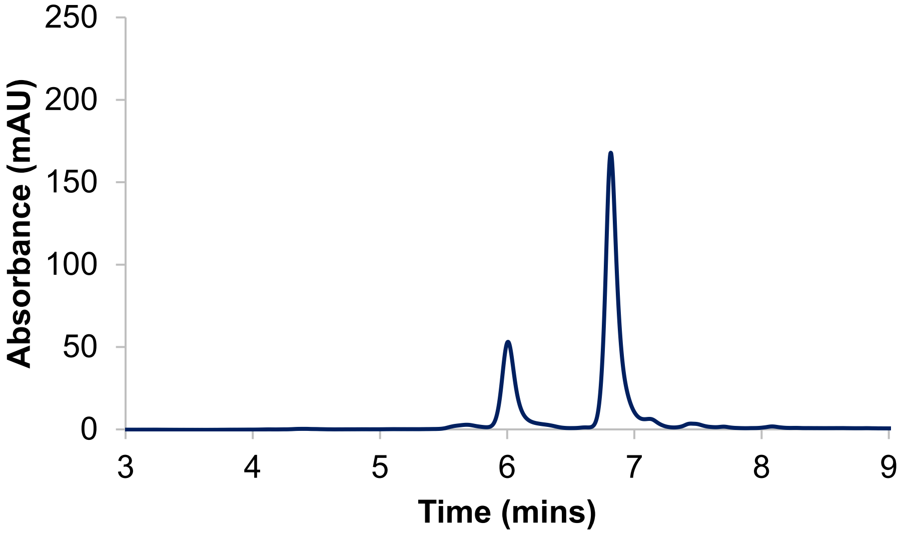


**Figure S23** - Analytical HPLC trace (crude reaction mixture) of Ac-MACY-NH2 (**6**) reacted with **2**; analytical gradient 5-60% B over 10 minutes, 280 nm.

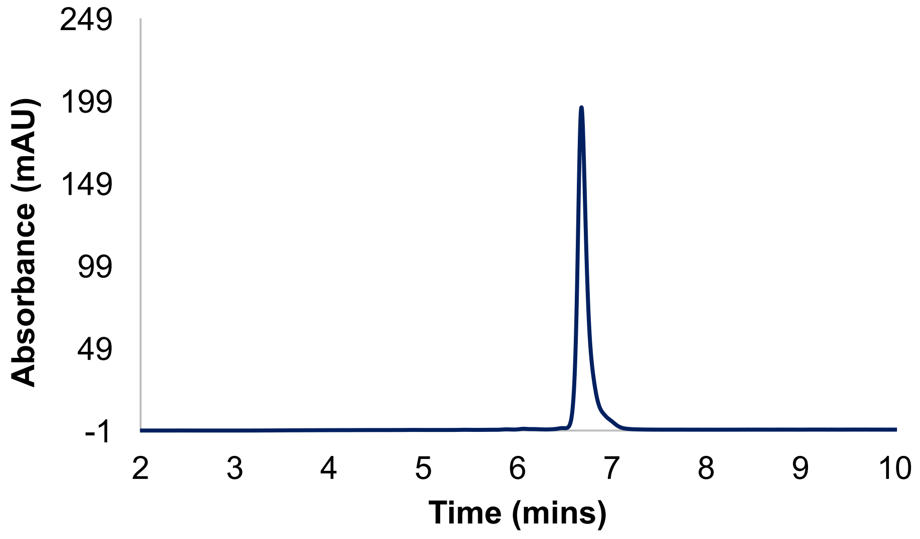


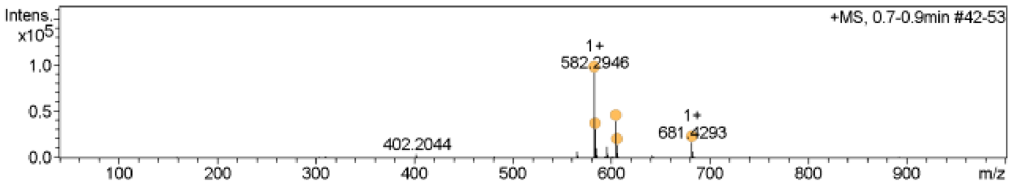


**Figure S24** - Analytical HPLC trace and ESI MS for purified product **7**; analytical gradient 5-60% B over 10 min, 280 nm. Calculated mass $[M+H]^+$: 582.29, observed mass $[M+H]^+$: 582.29.

## Ac-YEPLACHISKY-NH2 (8) modified with isoprenol (2)

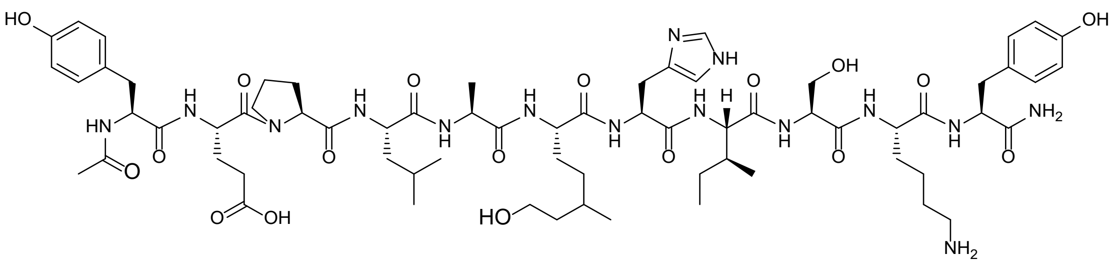

Product **9** was synthesized following the general conjugation procedure B over 10 mins using Ac-YEPLACHISKY-NH2 (**8**, 2 mg, 1.47 µmol) and isoprenol (**2**, 25 mg, 0.29 mmol), followed by purification using semi-preparative HPLC (15-80% B over 30 minutes) to yield the desired conjugated peptide (**9**, 1.6 mg, 1.13 µmol, 77% yield).

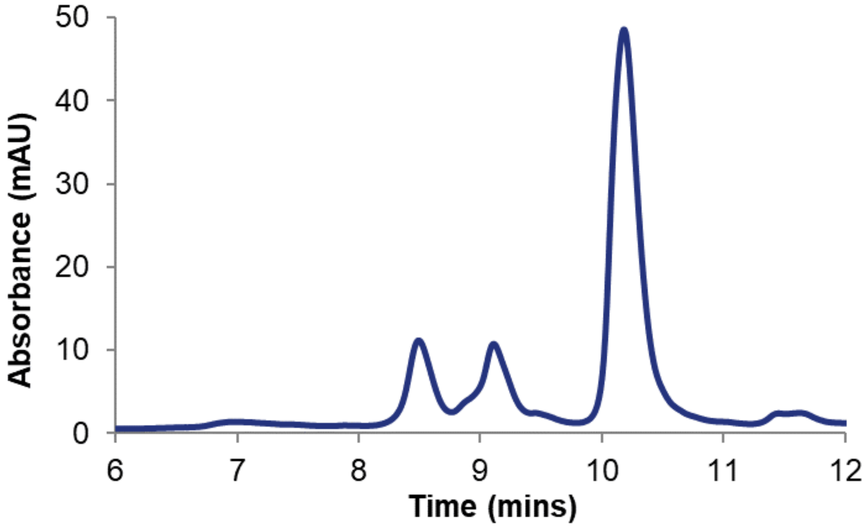


**Figure S25** - Analytical HPLC trace (crude reaction mixture) of Ac-YEPLACHISKY-NH2 (**8**) reacted with 3-methyl-3-buten-1-ol (**2**). Analytical gradient 15-25% B over 10 min, 280 nm.

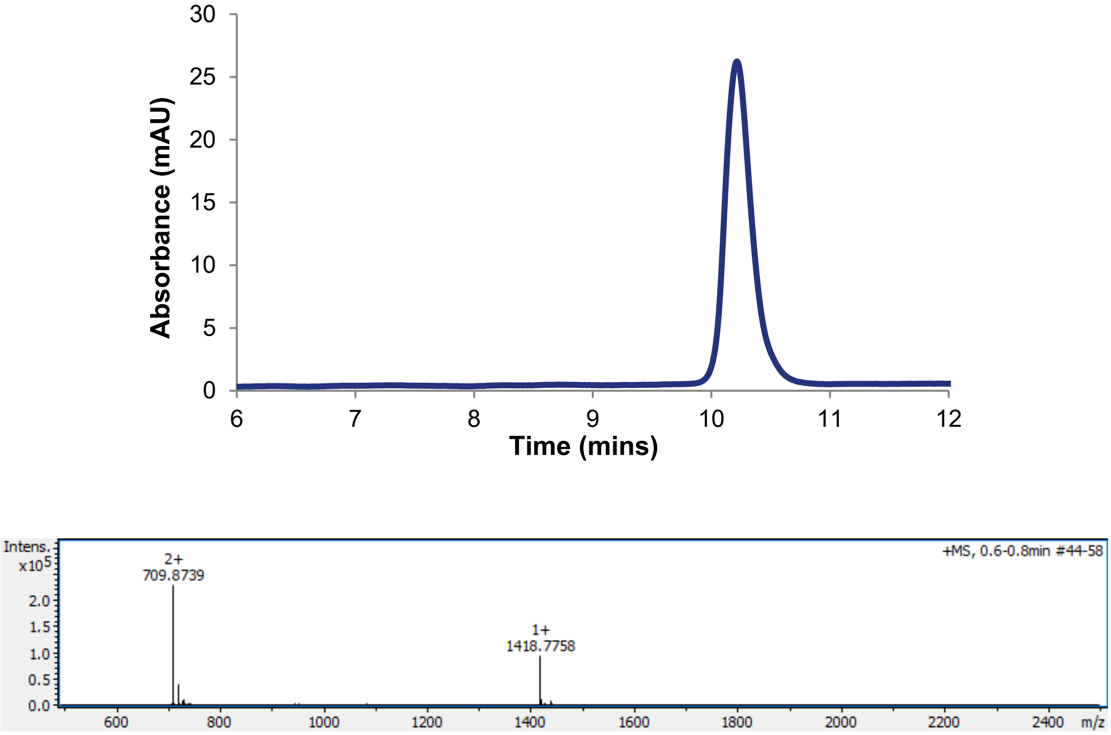


**Figure S26** - Analytical HPLC trace and ESI MS for purified product **9**; analytical gradient 15-25% B over 10 min, 210 nm. Calculated mass $[M+H]^+$: 1418.77, $[M+2H]^{2+}$: 709.89; observed mass $[M+H]^+$: 1418.78, $[M+2H]^{2+}$: 709.87.

**Ac-YEPLACHISKY-NH2 (6) modified with *N,N*-3-trimethylbut-3-en-1-amine (10)**

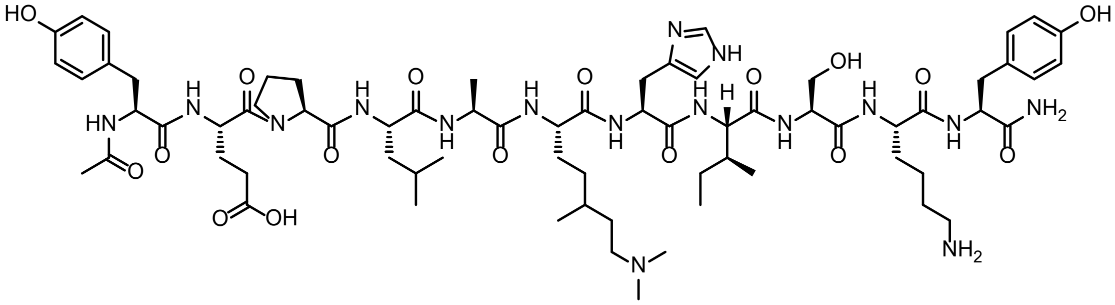

Product **18** was synthesized following general protocol B over 10 mins using Ac-YEPLACHISKY-NH2 (**8**, 2 mg, 1.466 µmol) and compound **10** (33 mg, 0.29 mmol). After analysis the remainder of material (1.365 µmol) was purified using semi-preparative HPLC (5-60% B over 30 minutes) to yield the desired conjugated peptide (**18**, 1.6 mg, 1.11 µmol, 81% yield).

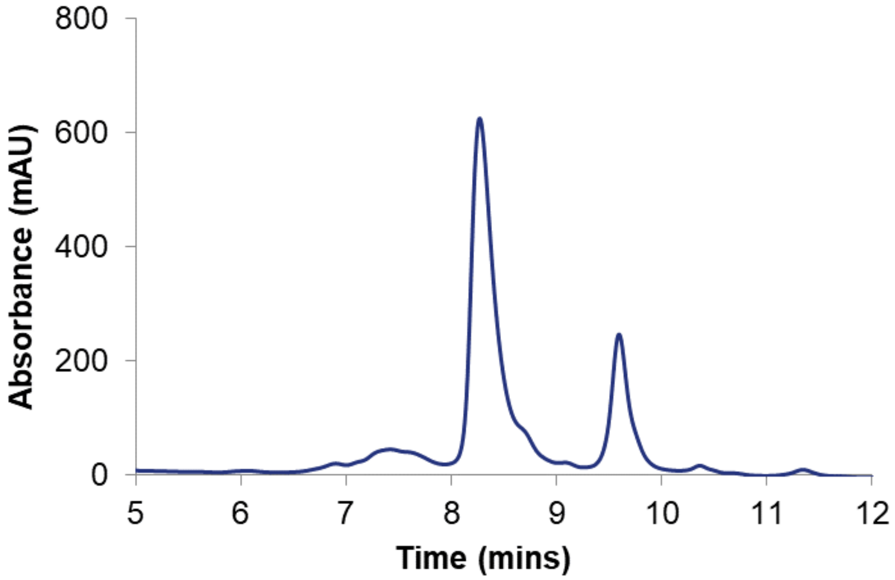


**Figure S27** - Analytical HPLC trace (crude reaction mixture) of Ac-YEPLACHISKY-NH2 (**8**) reacted with *N,N*-3-trimethylbut-3-en-1-amine (**10**). Analytical gradient 15-25% B over 10 min, 210 nm.

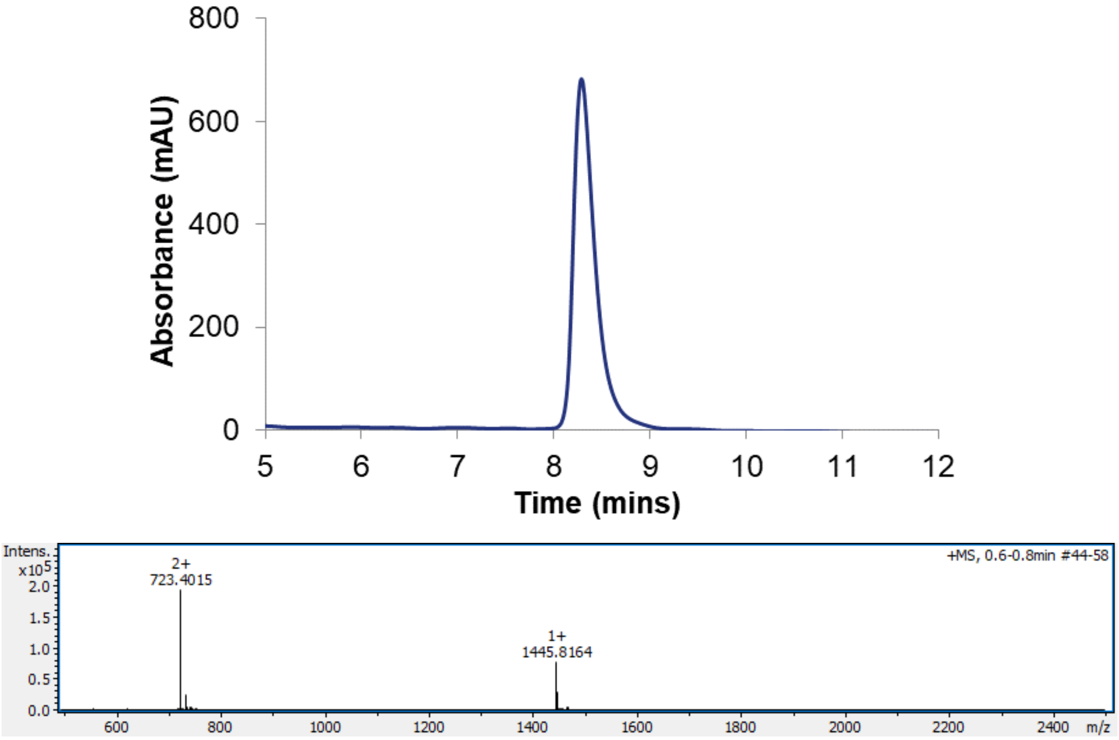


**Figure S28** - Analytical HPLC trace and ESI MS for purified product **18**; analytical gradient 15-25% B over 10 min, 210 nm. Calculated mass $[M+H]^{+}$: 1445.82, $[M+2H]^{2+}$: 723.41; observed mass $[M+H]^{+}$: 1445.82, $[M+2H]^{2+}$: 723.40.

**Ac-YEPLACHISKY-NH2 (8) modified with *N,N,N*-3-tetramethylbut-3-en-1-aminium iodide (11)**

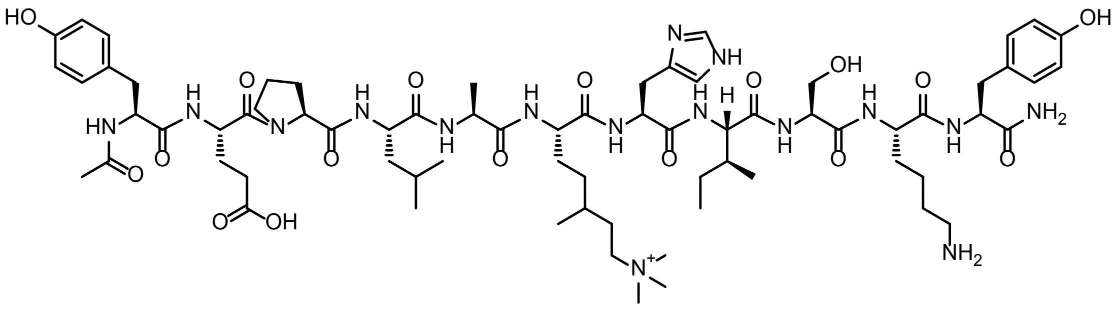

Product **19** was synthesised following the general protocol B over 60 mins using Ac-YEPLACHISKY-NH2 (**8**, 2 mg, 1.466 µmol) and compound **11** (75 mg, 0.29 mmol). After analysis the remainder of material (1.365 µmol) was purified using semi-preparative HPLC (5-60% B over 30 minutes) to yield the desired conjugated peptide (**19**, 1.7 mg, 1.07 µmol, 78% yield).

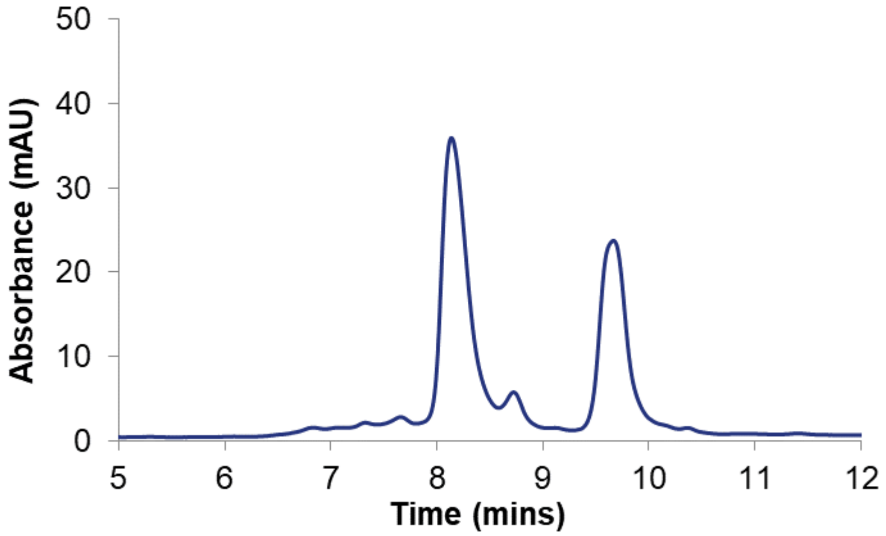


**Figure S29** - Analytical HPLC trace (crude reaction mixture) of Ac-YEPLACHISKY-NH2 (**8**) reacted with *N,N,N*-3-tetramethylbut-3-en-1-aminium iodide (**11**). Analytical gradient 15-25% B over 10 min, 210 nm.

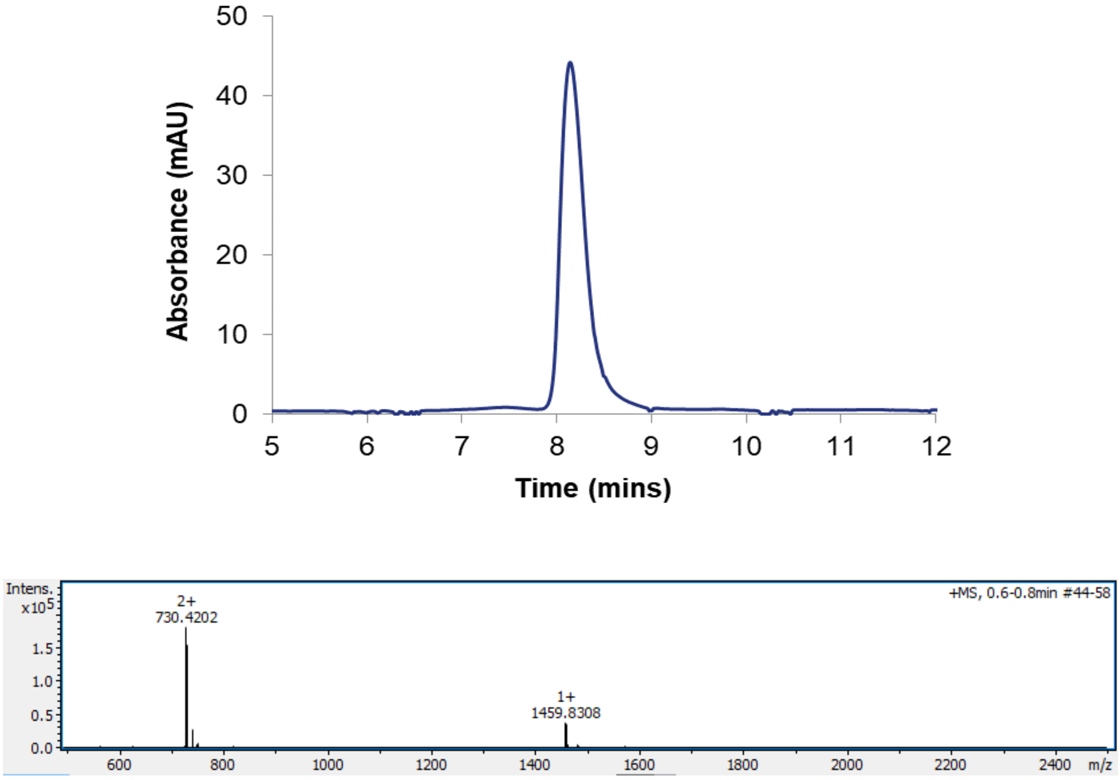


**Figure S30** - Analytical HPLC trace and ESI MS for purified product **19**; analytical gradient 15-25% B over 10 min, 210 nm. Calculated mass [M+H]$^{+}$: 1459.83, [M+H]$^{2+}$: 730.42; observed mass [M+H]$^{+}$: 1459.83, [M+H]$^{2+}$: 730.42.

**Ac-YEPLACHISKY-NH2 (8) modified with biotin probe (12)**

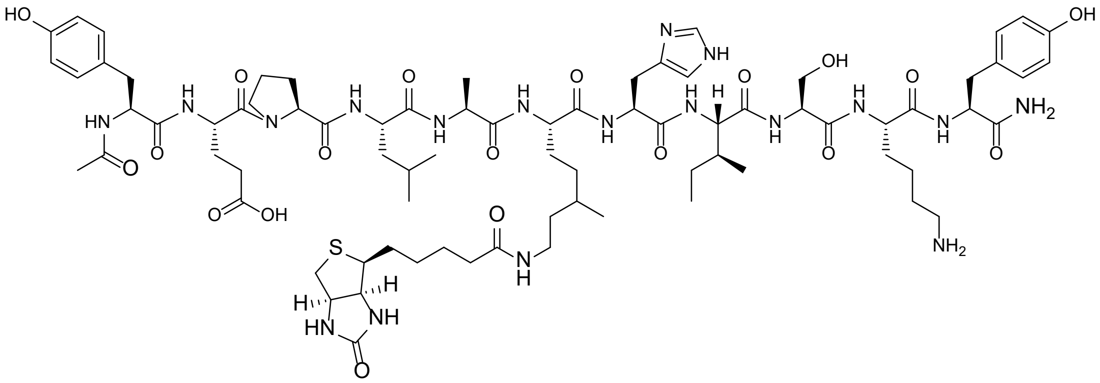

Product **20** was synthesised following the general protocol B over 10 mins using Ac-YEPLACHISKY-NH2 (**8**, 2 mg, 1.466 µmol) and compound **12** (91 mg, 0.29 mmol). After analysis the remainder of material (1.365 µmol) was purified using semi-preparative HPLC (15-80% B over 30 minutes) to yield the desired conjugated peptide (**20**, 1.8 mg, 1.094 µmol, 80% yield).

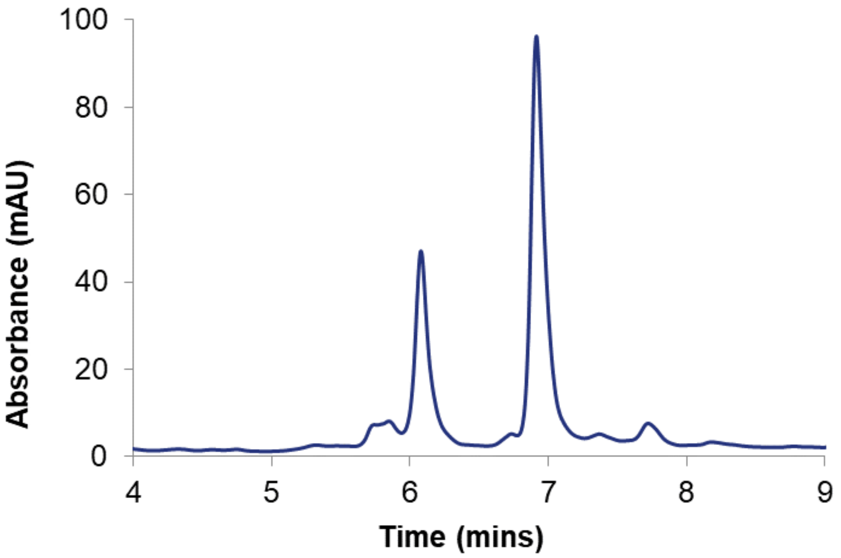


**Figure S31** - Analytical HPLC trace (crude reaction mixture) of Ac-YEPLACHISKY-NH2 (**8**) reacted with bute-biotin (**12**). Analytical gradient 15-45% B over 10 min, 280 nm.

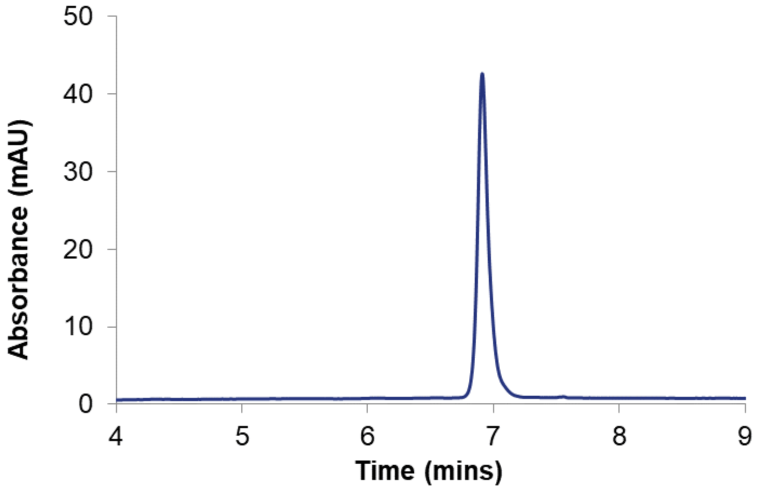


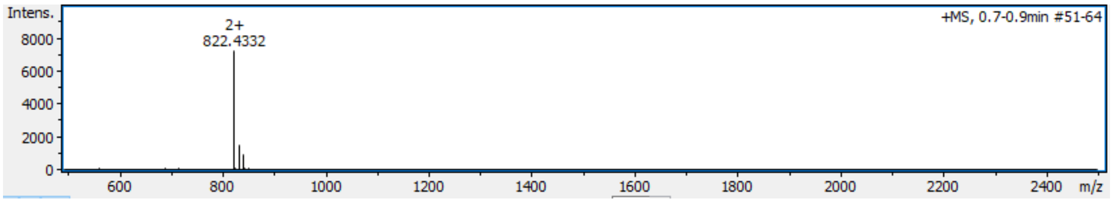


**Figure S32** - Analytical HPLC trace and ESI MS for purified product **20**; analytical gradient 15-45% B over 10 min, 210 nm. Calculated mass $[M+2H]^{2+}$: 822.43; observed mass $[M+2H]^{2+}$: 822.43.

**Ac-YEPLACHISKY-NH2 (8) modified with *N,N*,2-trimethylprop-2-en-1-amine (13)**

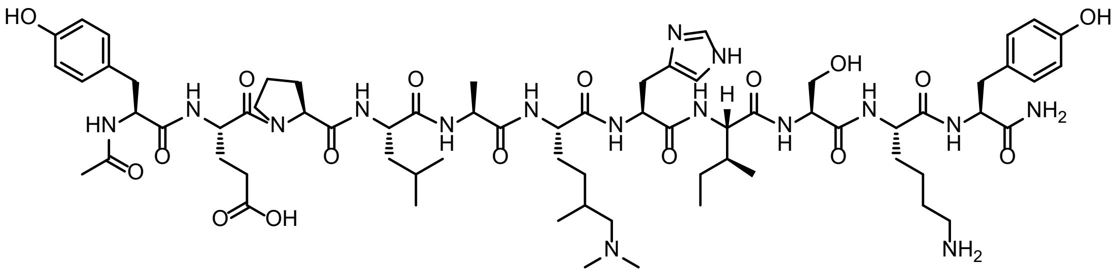

Product **21** was synthesised following the general protocol B over 30 mins using Ac-YEPLACHISKY-NH2 (**8**, 2 mg, 1.466 µmol and compound **13** (29 mg, 0.29 mmol). After analysis, the remainder of material (1.365 µmol) was purified using semi-preparative HPLC (5-60% B over 30 minutes) to yield the desired conjugated peptide (**21**, 1.3 mg, 0.908 µmol, 67% yield).

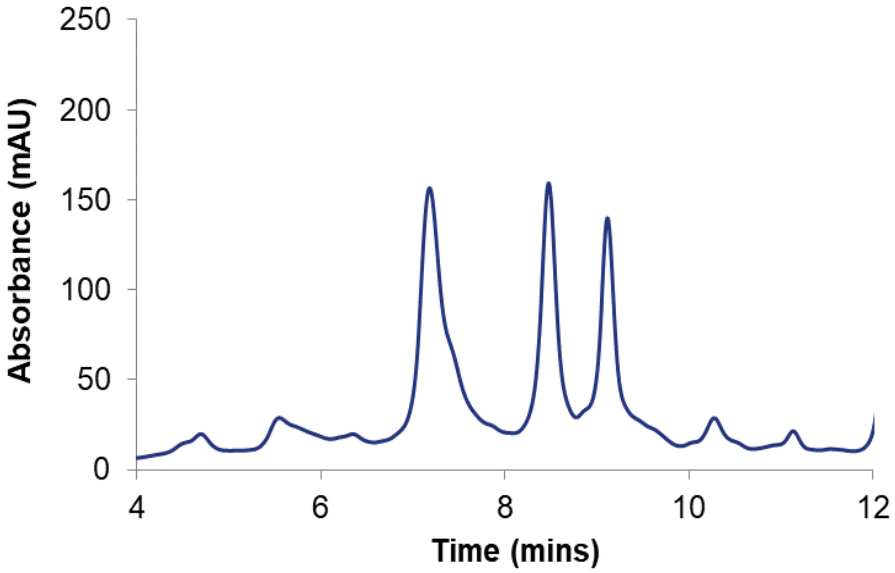


**Figure S33** - Analytical HPLC trace (crude reaction mixture) of Ac-YEPLACHISKY-NH2 (**8**) reacted with *N,N*,2-trimethylprop-2-en-1-amine (**13**). Analytical gradient 15-25% B over 10 min, 210 nm.

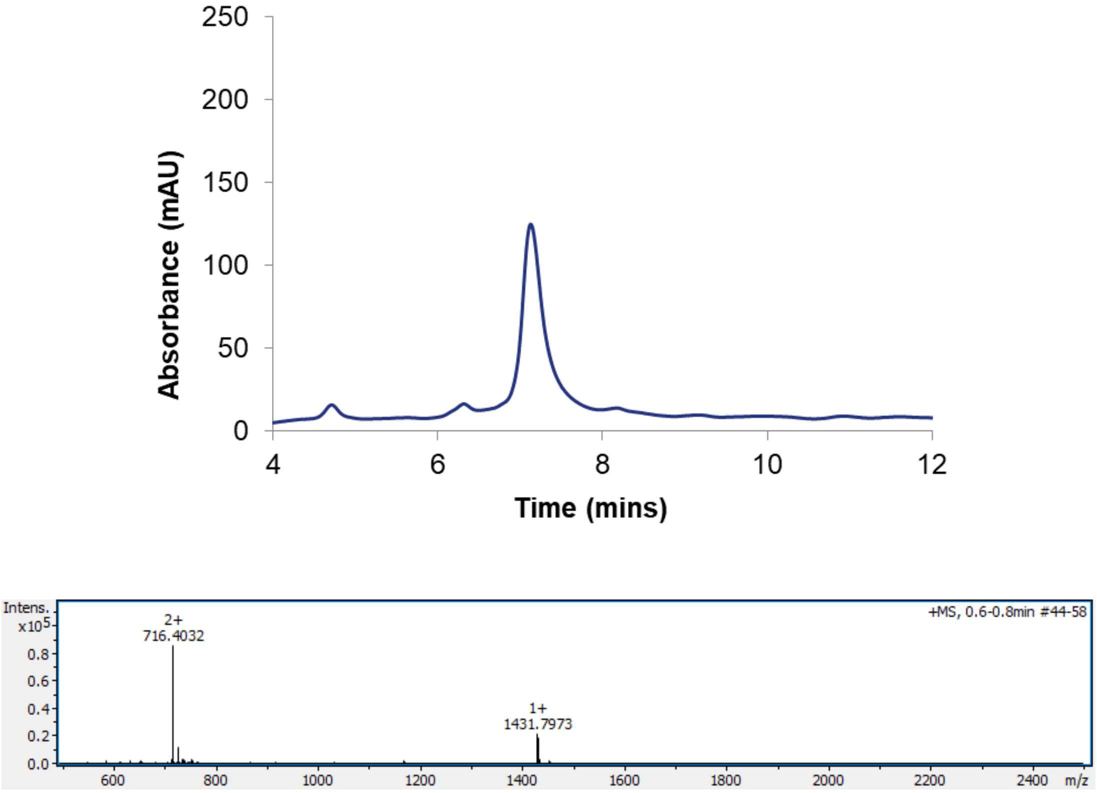


**Figure S34** - Analytical HPLC trace and ESI MS for purified product **21**; analytical gradient 15-25% B over 10 min, 210 nm. Calculated mass $[M+H]^{+}$: 1431.80, $[M+2H]^{2+}$: 716.40; observed mass $[M+H]^{+}$: 1431.80, $[M+2H]^{2+}$: 716.40.

**Ac-YEPLACHISKY-NH2 (8) modified with diethyl(2-methylallyl)phosphonate (14)**

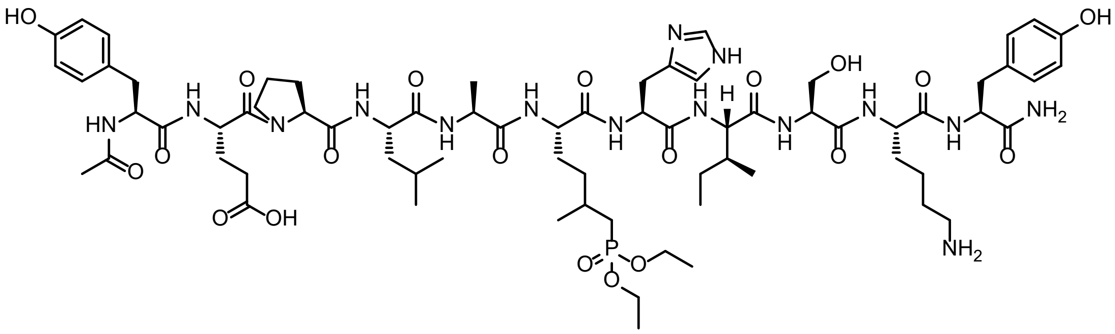

Product **22a** was synthesised following the general protocol B over 30 mins using Ac-YEPLACHISKY-NH2 (**8**, 2 mg, 1.466 µmol) and compound **14** (56 mg, 0.29 mmol). After analysis the remainder of material (1.365 µmol) was purified using semi-preparative HPLC (5-60% B over 30 minutes) to yield the desired conjugated peptide (**22a**, 1.7 mg, 1.11 µmol, 81% yield).

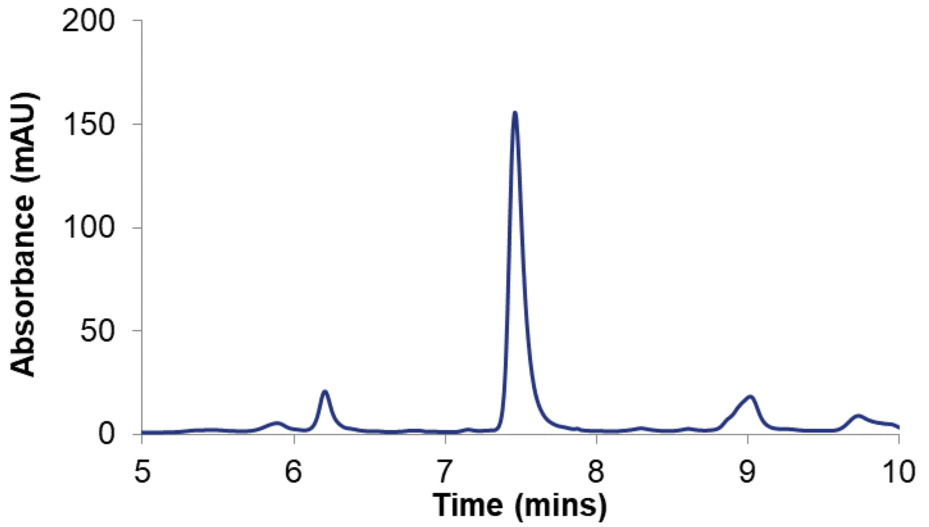


**Figure S35** - Analytical HPLC trace (crude reaction mixture) of Ac-YEPLACHISKY-NH2 (**8**) reacted with diethyl(2-methylallyl)phosphonate (**14**). Analytical gradient 15-45% B over 10 min, 210 nm.

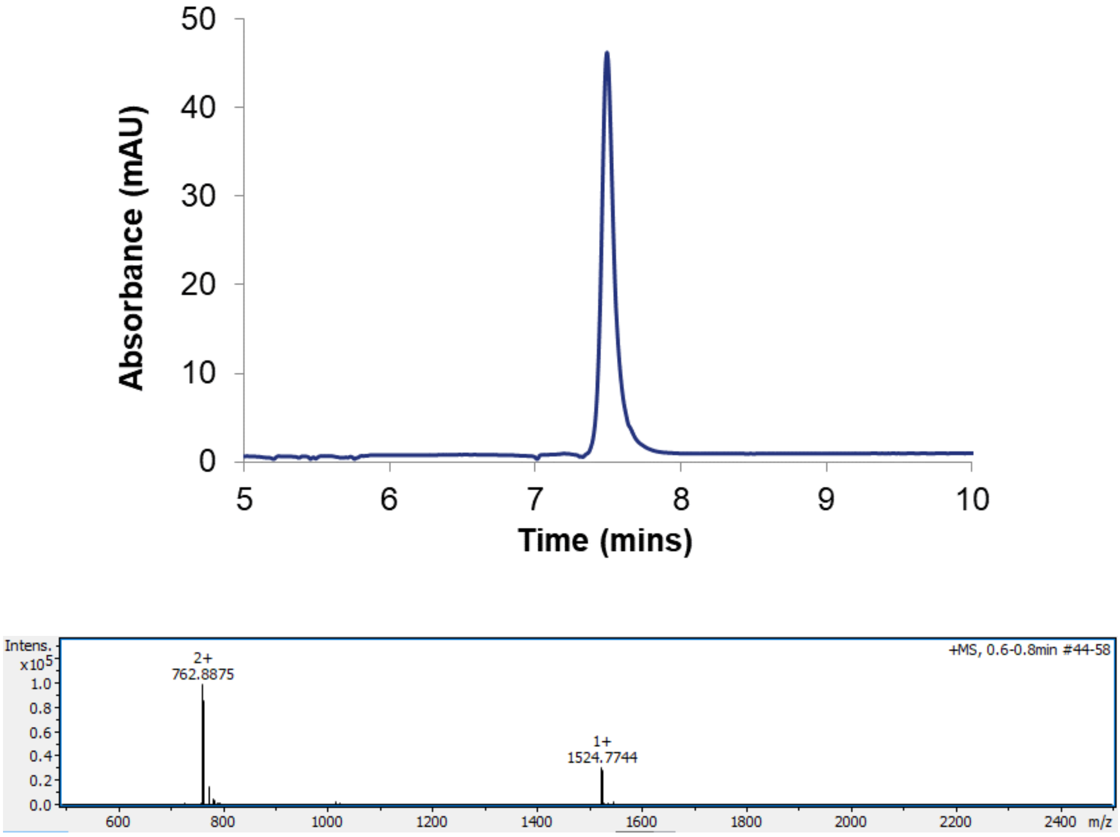


**Figure S36** - Analytical HPLC trace and ESI MS for purified product **22a**; analytical gradient 15-45% B over 10 min, 210 nm. Calculated mass $[M+H]^+$: 1524.79, $[M+2H]^{2+}$: 762.90; observed mass $[M+H]^+$: 1524.77, $[M+2H]^{2+}$: 762.89.

**Deprotection of Ac-YEPLACHIKSY-NH2 modified with diethyl (2-methylallyl) phosphonate (22b)**

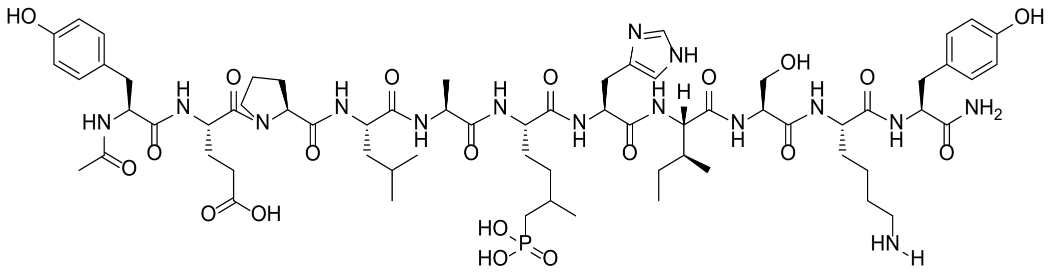

Peptide **22b** (1.3 mg, 0.853 µmol) was dissolved in anhydrous DCM (1 ml) and bromotrimethylsilane (1.35 µl, 0.01 mmol) was added. The reaction mixture was stirred at 35 °C for 16 h, before cooling to room temperature and concentrating *in vacuo*. The crude solid was then dissolved in a 1:1 mixture of methanol and water and stirred at room temperature for 4 h. The crude peptide was purified by preparative RP-HPLC (15-80% B over 30 minutes) and lyophilized to produce the desired peptide (**22b**, 0.6 mg, 0.853 µmol, 48% yield).

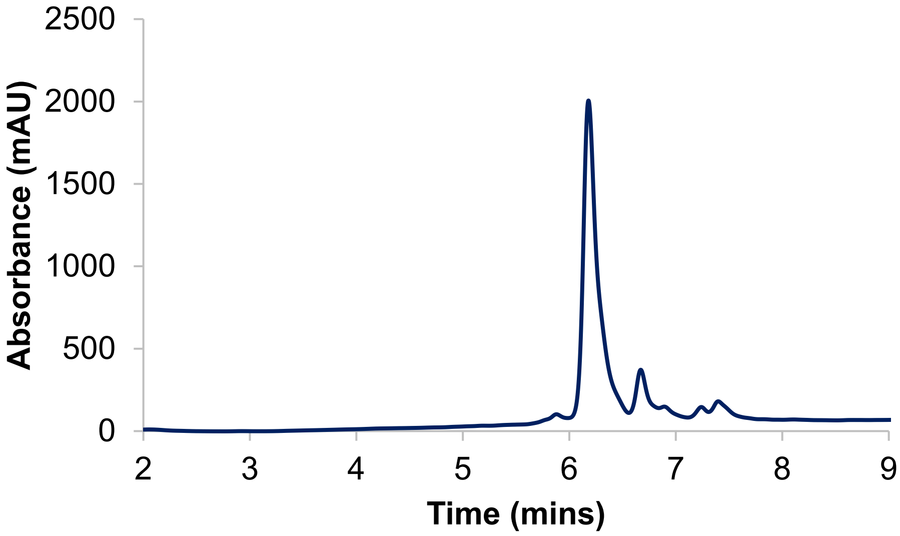


**Figure S37** - Analytical HPLC trace (crude) of the deprotection of 22a; analytical gradient 15-80% B over 10 minutes, 280 nm.

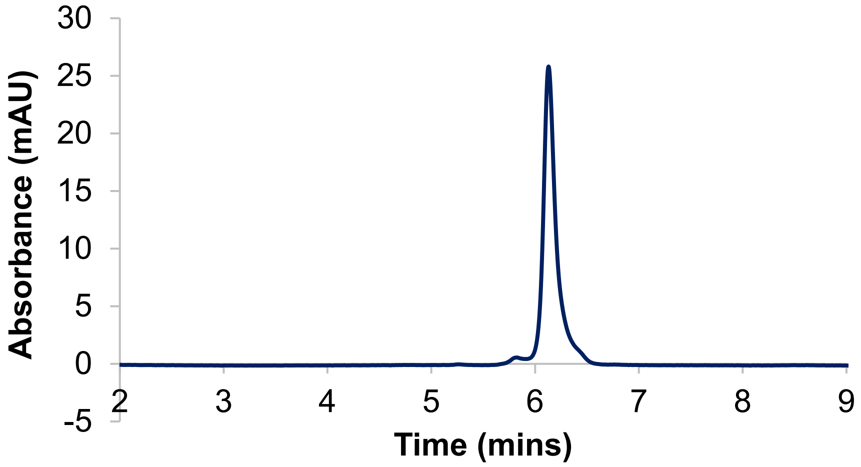


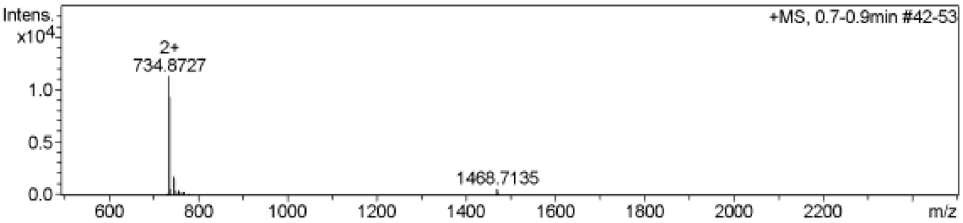


**Figure S38** - Analytical HPLC trace and ESI MS for purified product **22b**; analytical gradient 15-45% B over 10 min, 280 nm. Calculated mass $[M+H]^+$: 1468.72, $[M+2H]^{2+}$: 734.86; observed mass $[M+H]^+$: 1468.71, $[M+2H]^{2+}$: 734.87

## Ac-YEPLACHISKY-NH2 (8) modified with biotin probe 15

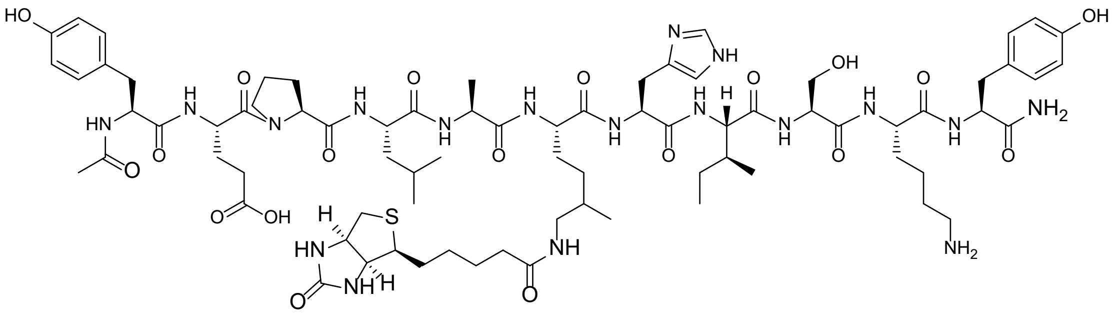

Product **23** was synthesised following the general protocol B over 30 mins using Ac-YEPLACHISKY-NH2 (**8**, 2 mg, 1.466 µmol) and compound **15** (87 mg, 0.29 mmol). After analysis the remainder of material (1.365 µmol) was purified using semi-preparative HPLC (15-80% B over 30 minutes) to yield the desired conjugated peptide (**23**, 1.4 mg, 0.858 µmol, 63% yield).

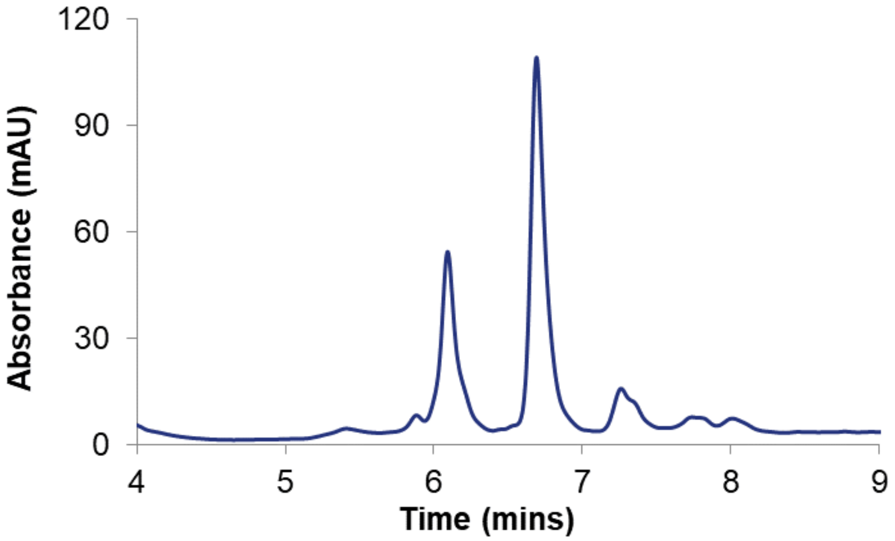


**Figure S39** - Analytical HPLC trace (crude reaction mixture) of Ac-YEPLACHISKY-NH2 (**8**) reacted with prop-biotin (**15**) after 1 hour. Analytical gradient 15-45% B over 10 min, 280 nm.

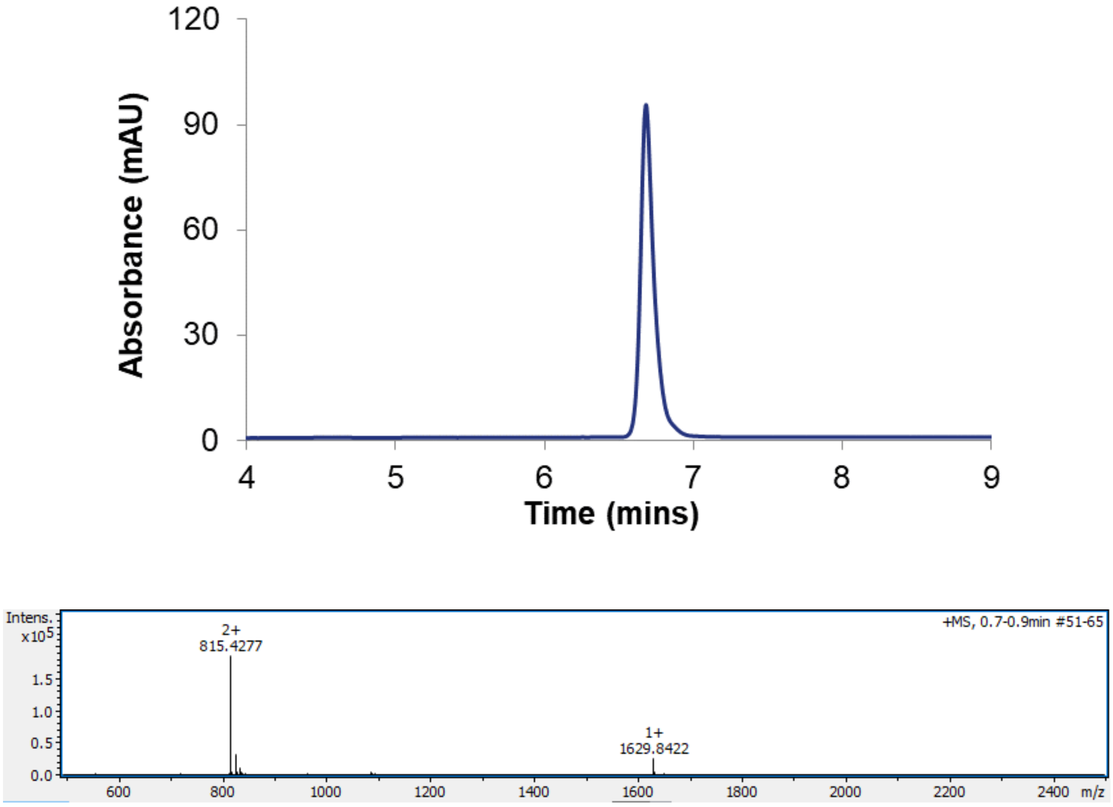


**Figure S40** - Analytical HPLC trace and ESI MS for purified product **23**; analytical gradient 15-45% B over 10 min, 210 nm. Calculated mass $[M+H]^+$: 1629.85, $[M+2H]^{2+}$: 815.43; observed mass $[M+H]^+$: 1629.84, $[M+2H]^{2+}$: 815.43.

**Ac-YEPLACHISKY-NH2 (6) modified with 4-((3-methylbut-3-en-1-yl)amino)-4-oxobutanoic acid (16)**

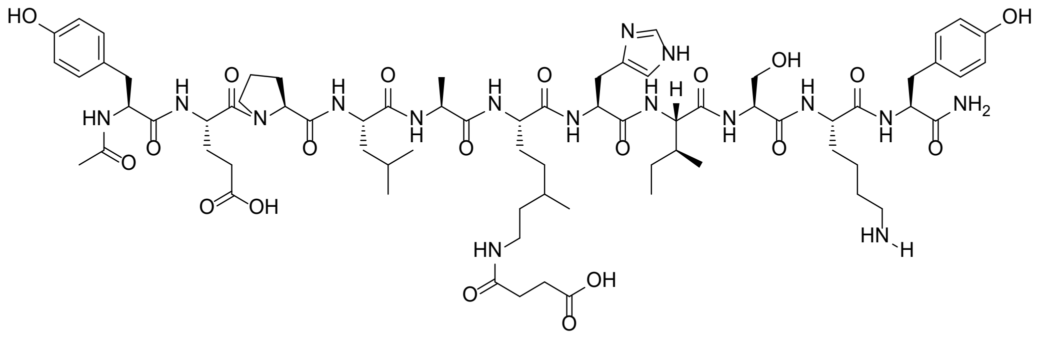

Product **24** was synthesised following the general protocol B over 30 mins using Ac-YEPLACHISKY-NH2 (**6**, 2 mg, 1.466 µmol) and compound **16** (54 mg, 0.29 mmol). After analysis the remainder of material (1.365 µmol) was purified using semi-preparative HPLC (15-80% B over 30 minutes) to yield the desired conjugated peptide (**24**, 1.6 mg, 1.06 µmol, 75% yield).

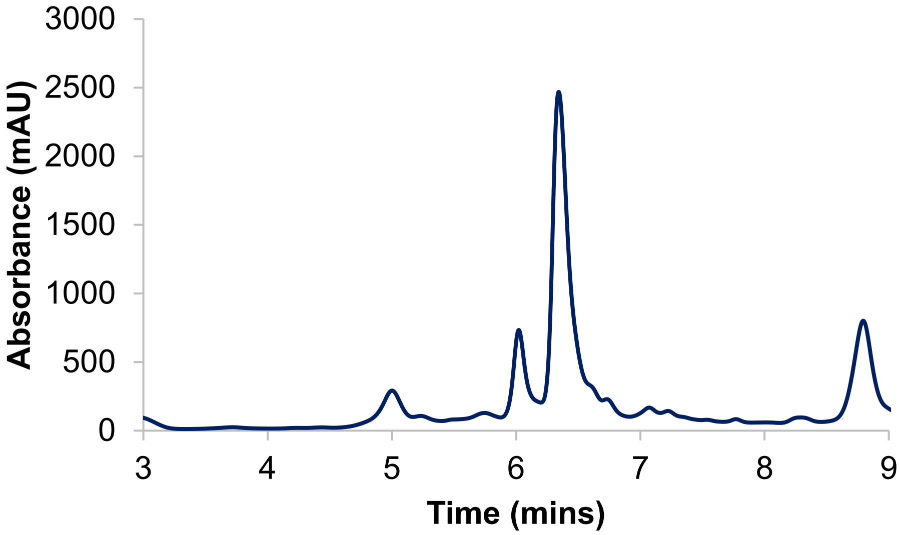


**Figure S41** – Analytical HPLC trace (crude reaction mixture) of Ac-YEPLACHISKY-NH2 (**6**) reacted with 4-((3-methylbut-3-en-1-yl)amino)-4-oxobutanoic acid (**16**); analytical gradient 15-45% B over 10 minutes, 210 nm

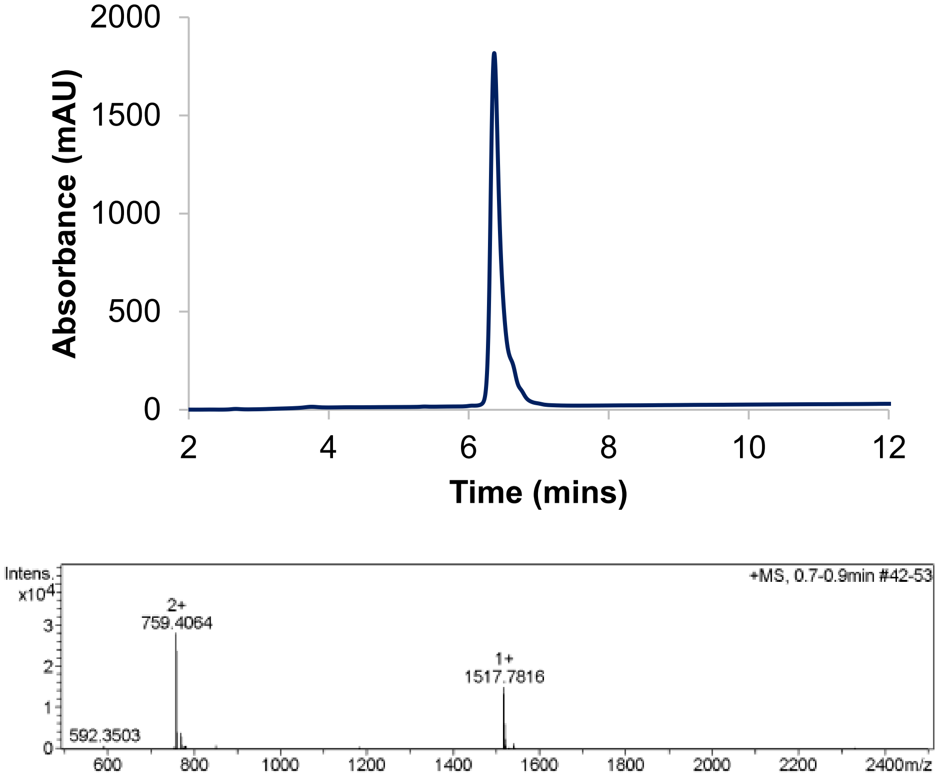


**Figure S42** - Analytical HPLC trace and ESI MS for purified product **24**; analytical gradient 15-45% B over 10 min, 210 nm. Calculated mass $[M+H]^+$: 1517.99, $[M+2H]^{2+}$: 759.50; observed mass $[M+H]^+$: 1517.78, $[M+2H]^{2+}$: 759.41

**Ac-YEPLACHISKY-NH2 (6) modified with *N*-(3-methylbut-3-en-1-yl)benzamide (17)**

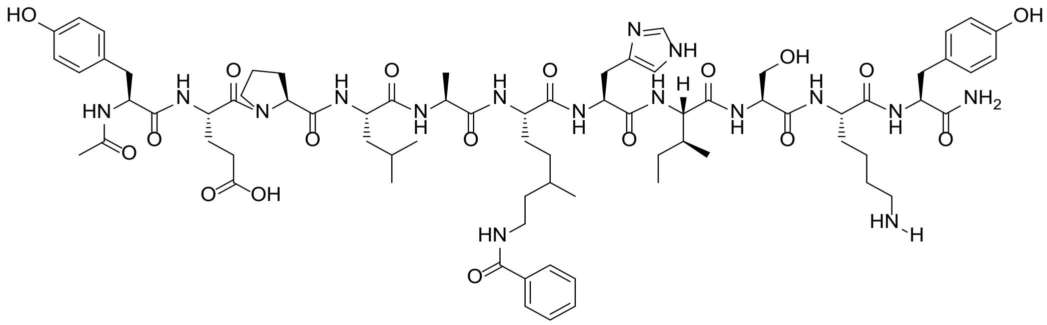

Product **25** was synthesised following the general protocol B over 30 mins using Ac-YEPLACHISKY-NH2 (**6**, 2 mg, 1.466 μmol) and compound **17** (55 mg, 0.29 mmol). After analysis the remainder of material (1.365 μmol) was purified using semi-preparative HPLC (15-80% B over 30 minutes) to yield the desired conjugated peptide (**25**, 1.1 mg, 0.723 μmol, 51% yield*). **Note that the probe* ***17*** *wasn't fully miscible with reaction solvent.*

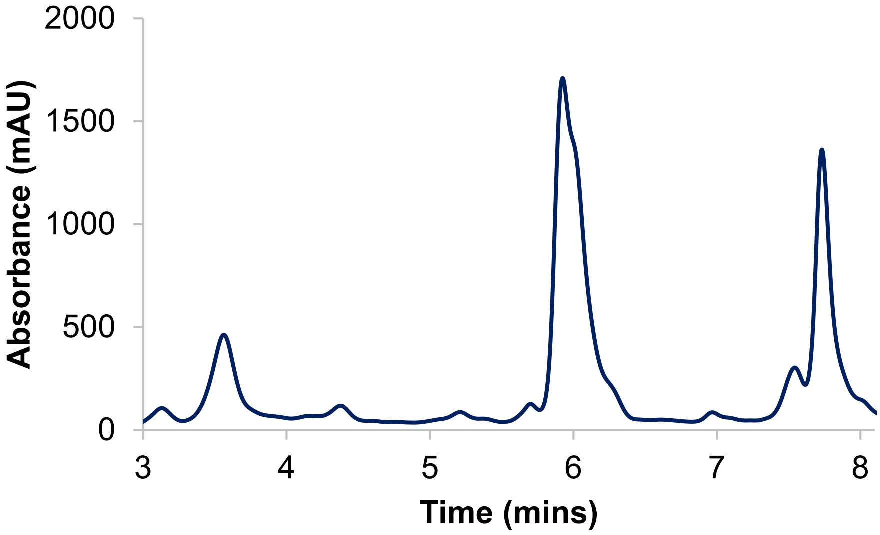


**Figure S43** – Analytical HPLC trace (crude reaction mixture) of Ac-YEPLACHISKY-NH2 (**6**) reacted with *N*-(3-methylbut-3-en-1-yl)benzamide (**17**) ; analytical gradient 15-45% B over 10 minutes, 210 nm.

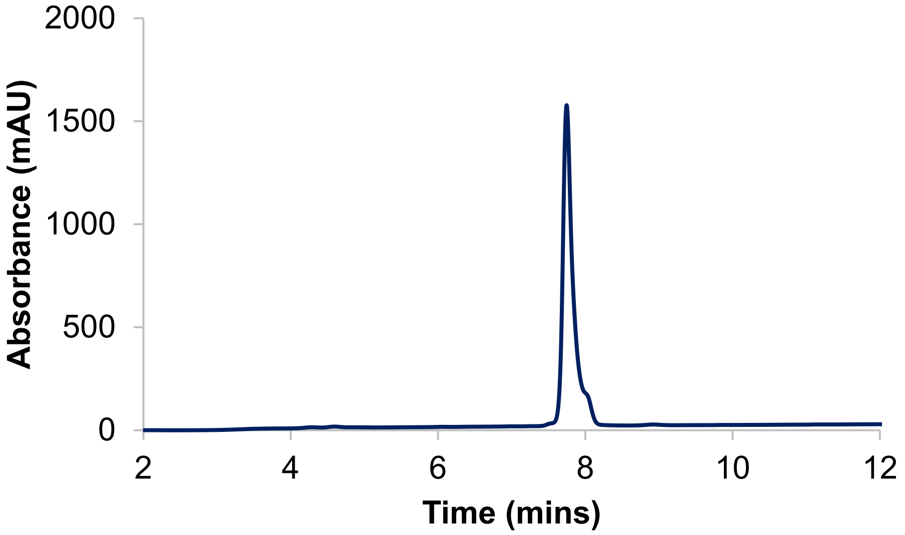


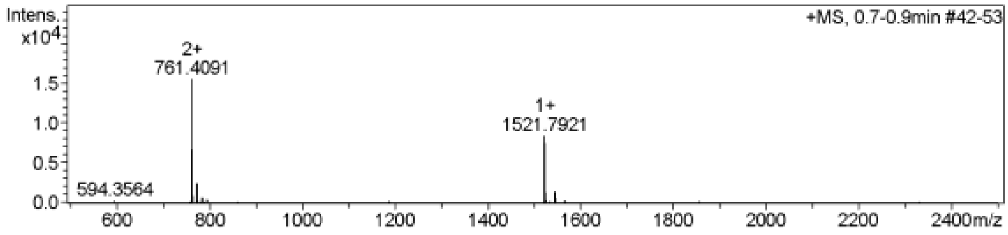


**Figure S44** - Analytical HPLC trace and ESI MS for purified product **25**; analytical gradient 15-45% B over 10 min, 210 nm. Calculated mass $[M+H]^+$: 1521.81, $[M+2H]^{2+}$: 761.90; observed mass $[M+H]^+$: 1521.79, $[M+2H]^{2+}$: 761.41.

**Ac-YEPLACHISKY-NH2 (8) modified with 2-methyl-2-propene-1-sulfonic acid (27)**

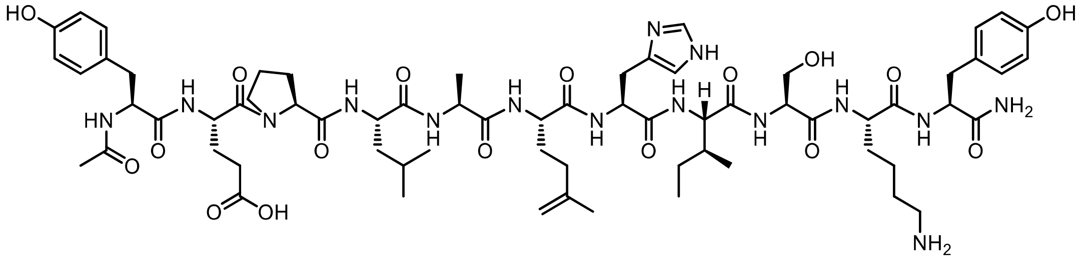

Product **28** was synthesised following the general protocol B over 30 mins using Ac-YEPLACHISKY-NH2 (**8**, 2 mg, 1.466 μmol) and compound **27** (46 mg, 0.29 mmol). After analysis the remainder of material (1.365 μmol) was purified using semi-preparative HPLC (5-60% B over 30 minutes) to yield the desired conjugated peptide (**28**, 1.5 mg, 1.08 μmol, 79% yield).

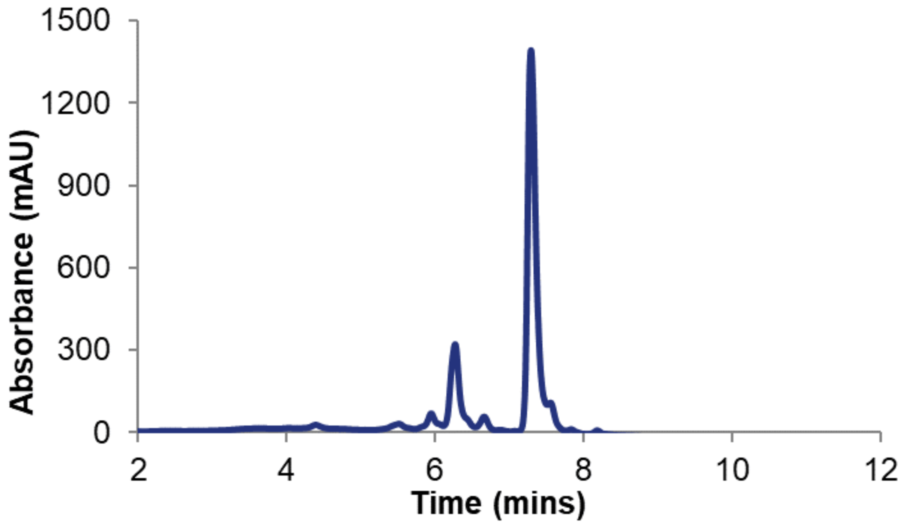


**Figure S45** - Analytical HPLC trace (crude reaction mixture) of Ac-YEPLACHISKY-NH2 (**8**) reacted with 2-methyl-2-propene-1-sulfonic acid (**27**). Analytical gradient 15-45% B over 10 min, 210 nm.

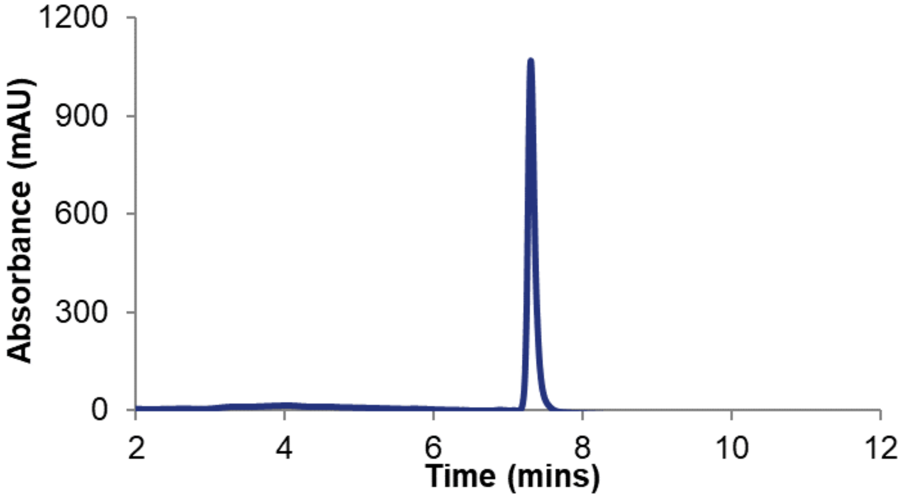


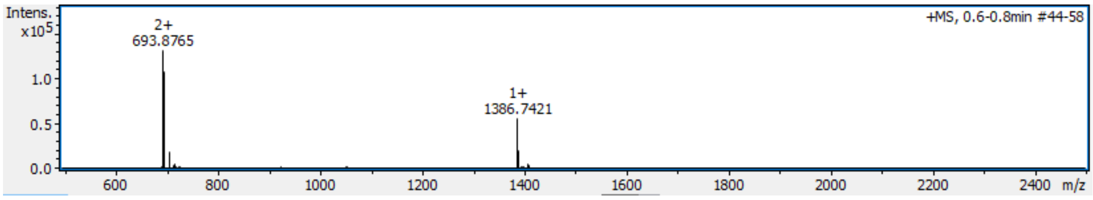


**Figure S46** - Analytical HPLC trace and ESI MS for purified product **28**; analytical gradient 15-45% B over 10 min, 210 nm. Calculated mass $[M+H]^+$: 1386.74, $[M+2H]^{2+}$: 693.88; observed mass $[M+H]^+$: 1386.74, $[M+2H]^{2+}$: 693.88.

**Hydrofluorination of product 28**

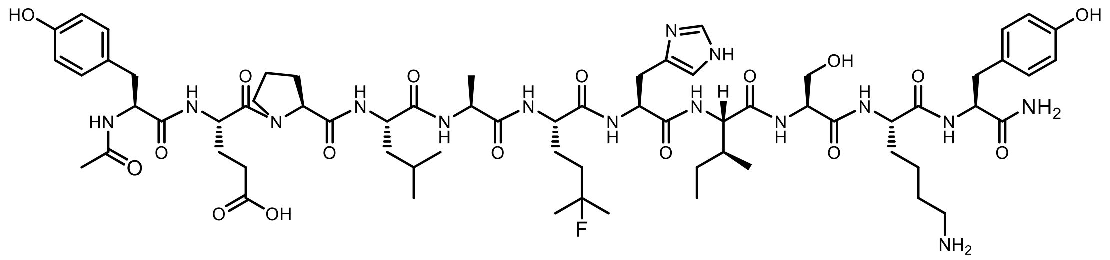

Hydrofluorination was performed as previously described.[9] The following solutions were prepared: 0.05 M iron (III) oxalate (2.4 mg per 0.1 ml $H_2O$, degassed with Ar), 0.5 M Selectfluor (17.7 mg per 0.1 ml $H_2O$) and 0.5 M $NaBH_4$ (1.9 mg per 0.1 ml MeCN). The final concentrations of the initial reaction mixture were 0.0125 M peptide (**28**), 0.025 M iron (III) oxalate, 0.025 M Selectfluor and 0.04 M $NaBH_4$. To peptide **28** (1 mg, 0.722 μmol) in acetonitrile (0.024 ml) was added iron (III) oxalate (0.029 ml, 0.05 M stock, 2 eq.) and Selectfluor (0.003 ml, 0.5 M stock, 2 eq.) and the solution briefly degassed with Ar. The reaction mixture was placed on ice and $NaBH_4$ (0.005 ml, 0.5 M stock, 1.6 eq.) was added. The reaction mixture was shaken at 0 ºC for 2 minutes and a further quantity of $NaBH_4$ (0.005 ml, 0.5 M stock, 2 eq.) was added. The reaction mixture was shaken on ice for a further 30 minutes, after which time the reaction was analysed by HPLC and purified by semi-preparative HPLC (5-60% B over 30 minutes). The fractions containing the desired peptide were lyophilised to give the title compound as a fluffy white solid (**30**, 0.7 mg, 0.498 μmol, 69% yield).

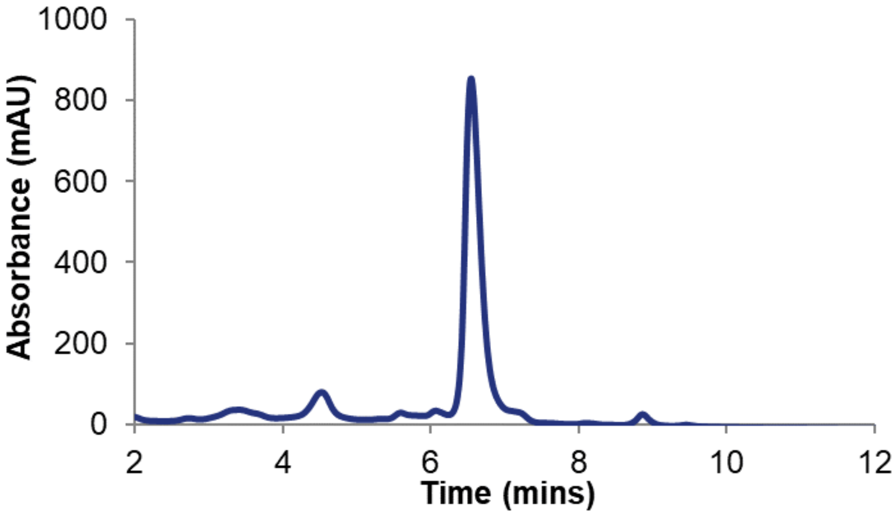


**Figure S47** – Analytical HPLC trace (crude) of Ac-YEPLAXHISKY-$NH_2$ (**28**) hydrofluorinated using literature conditions after 30 minutes. Analytical gradient 20-30% B over 10 min, 210 nm.

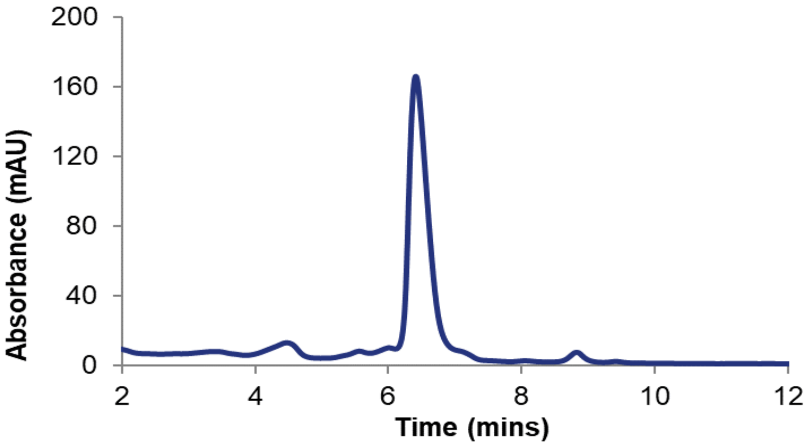


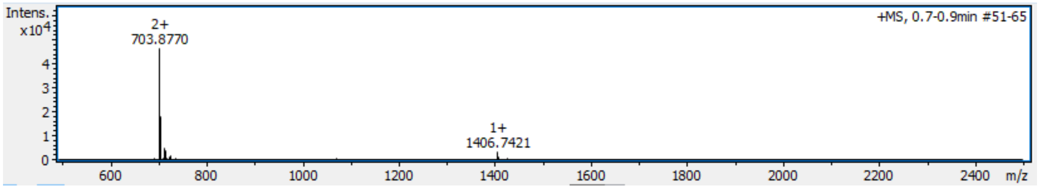


**Figure S48** – Analytical HPLC trace and ESI MS for purified product **30**; analytical gradient 20-30% B over 10 min, 210 nm. Calculated mass $[M+H]^+$: 1406.75, $[M+2H]^{2+}$:703.88; observed mass $[M+H]^+$: 1386.74, $[M+2H]^{2+}$: 703.88.

**Ac-CAY-NH2 (4a) modified with 2-methylprop-2-ene-2-sulfonic acid (27)**

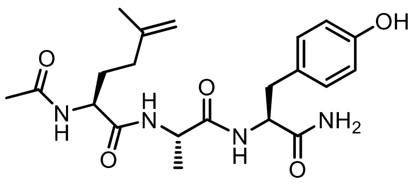


Product **29** was synthesised following the general protocol B using Ac-CAY-$NH_2$ (**4a**, 5 mg, 12.6 µmol) and compound **27** (399 mg, 2.52 mmol), and purified using semi-preparative HPLC (5-60% B over 30 minutes) to yield the desired conjugated peptide (**29**, 4.1 mg, 9.75 µmol, 77% yield). $^1$H NMR (500 MHz, $CD_3OD$) δ 7.07 (d, *J* = 8.5 Hz, 2H), 6.71 (d, *J* = 8.5 Hz, 2H), 4.80 – 4.72 (m, 2H), 4.49 (dd, *J* = 8.6, 5.7 Hz, 1H), 4.29 – 4.15 (m, 2H), 3.08 (dd, *J* = 14.1, 5.6 Hz, 1H), 2.91 (dd, *J* = 14.0, 8.6 Hz, 1H), 2.11 (dt, *J* = 9.4, 6.3 Hz, 2H), 2.03 (s, 3H), 1.97 – 1.88 (m, 1H), 1.83 – 1.78 (m, 1H), 1.76 (t, *J* = 1.1 Hz, 3H), 1.28 (d, *J* = 7.2 Hz, 3H). $^{13}$C NMR (126 MHz, $CD_3OD$) δ 174.7, 173.4, 173.3, 172.4, 155.9, 144.3, 129.9, 127.7, 114.8, 109.9, 54.6, 53.7, 49.5, 36.4, 33.4, 29.5, 21.2, 21.1, 16.2.

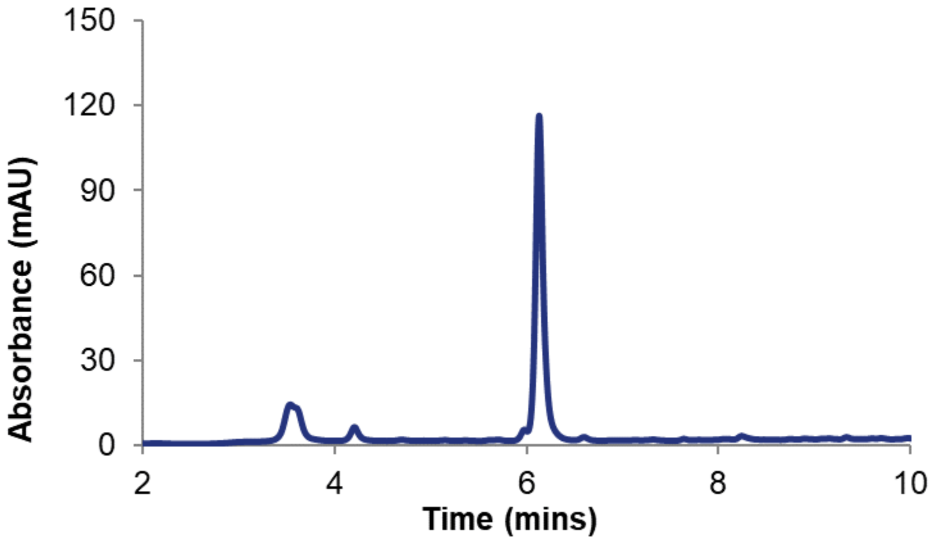


**Figure S49** - Analytical HPLC trace (crude) of Ac-CAY-$NH_2$ (**4a**) reacted with 2-methyl-2-propene-1-sulfonic acid sodium salt (**27**). Analytical gradient 5-60% B over 10 min, 280 nm.

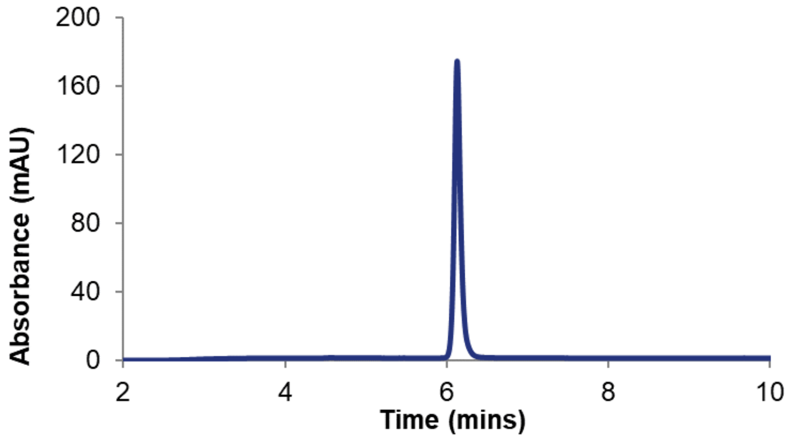


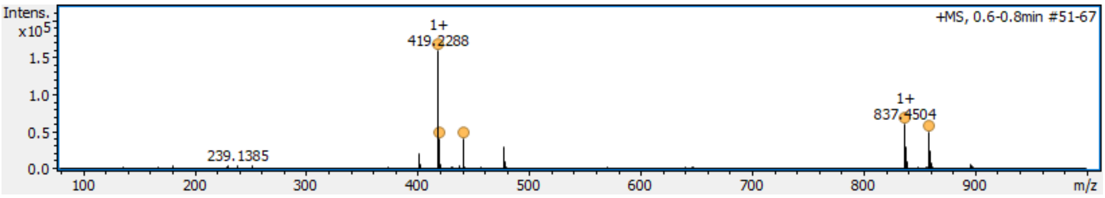


**Figure S50** - Analytical HPLC trace and ESI MS for purified product **29**; analytical gradient 5-60% B over 10 min, 280 nm. Calculated mass $[M+H]^+$: 419.23; observed mass $[M+H]^+$: 419.23.

## Hydrofluorination of product 29

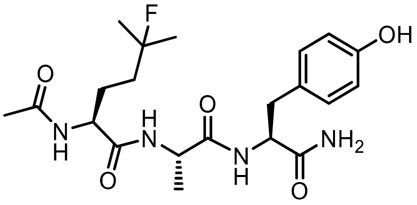


Hydrofluorination was performed as previously described.[1] The following solutions were prepared: 0.05 M solution of iron (III) oxalate (2.4 mg per 0.1 ml $H_2O$, degassed with Ar), 0.5 M Selectfluor (17.7 mg per 0.1 ml $H_2O$) and 0.5 M $NaBH_4$ (1.9 mg per 0.1 ml MeCN). The final concentrations of the initial reaction mixture were 0.0125 M peptide (**29**), 0.025 M iron (III) oxalate, 0.025 M Selectfluor and 0.04 M $NaBH_4$. To peptide **29** (2 mg, 4.78 µmol) was added iron (III) oxalate (2 eq.) and Selectfluor (2 eq.) and the solution briefly degassed with Ar. The reaction mixture was placed on ice and $NaBH_4$ (1.6 eq.) was added. The reaction mixture was shaken at 0 ºC for 2 minutes and a further quantity of $NaBH_4$ (2 eq.) was added. The reaction mixture was shaken on ice for a further

30 minutes, after which time the reaction was analysed by HPLC and purified by semi-preparative HPLC (5-60% B over 30 minutes) to yield the title compound as a fluffy white solid (**31**, 1.5 mg, 3.42 μmol, 72% yield). $^{1}H$ NMR (500 MHz, $CD_3OD$) δ 7.08 (d, J = 8.5 Hz, 2H), 6.71 (d, J = 8.5 Hz, 2H), 4.48 (dd, J = 8.5, 5.8 Hz, 1H), 4.24 (dd, J = 8.2, 6.2 Hz, 2H), 3.08 (dd, J = 14.0, 5.8 Hz, 1H), 2.91 (dd, J = 14.0, 8.5 Hz, 1H), 2.03 (s, 3H), 1.95 – 1.84 (m, 1H), 1.81 – 1.61 (m, 3H), 1.37 (d, J = 1.8 Hz, 3H), 1.33 (d, J = 1.8 Hz, 3H), 1.29 (d, J = 7.2 Hz, 3H). $^{13}C$ NMR (126 MHz, $CD_3OD$) δ 174.7 (C), 173.2 (C), 173.1 (C), 172.3 (C), 155.9 (Ar), 129.9 (Ar), 127.7 (Ar-H), 114.78 (Ar-H), 93.4 (C), 54.6 (CH), 54.0 (CH), 49.5 (CH), 37.1 (CH), 36.9 ($CH_2$), 36.4 ($CH_2$), 29.3 ($CH_3$), 26.0 ($CH_2$), 25.4 ($CH_3$), 21.0 ($CH_3$), 16.2 ($CH_3$). $^{19}F$ NMR (376 MHz, $CD_3OD$) δ -140.15.

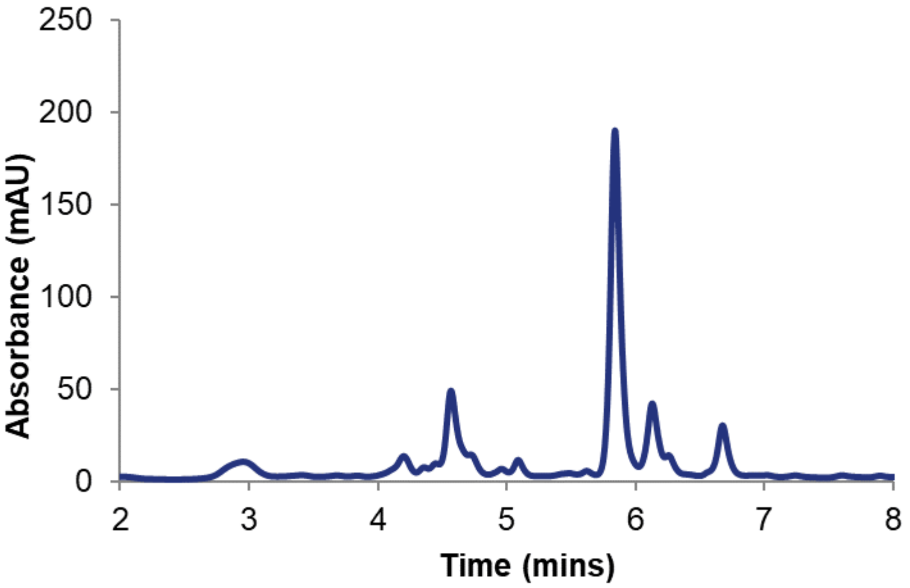


**Figure S51** - Analytical HPLC trace (crude) of Ac-XAY-$NH_2$ (**29**) hydrofluorinated after 1 hour. Analytical gradient 5-60% B over 10 min, 280 nm.

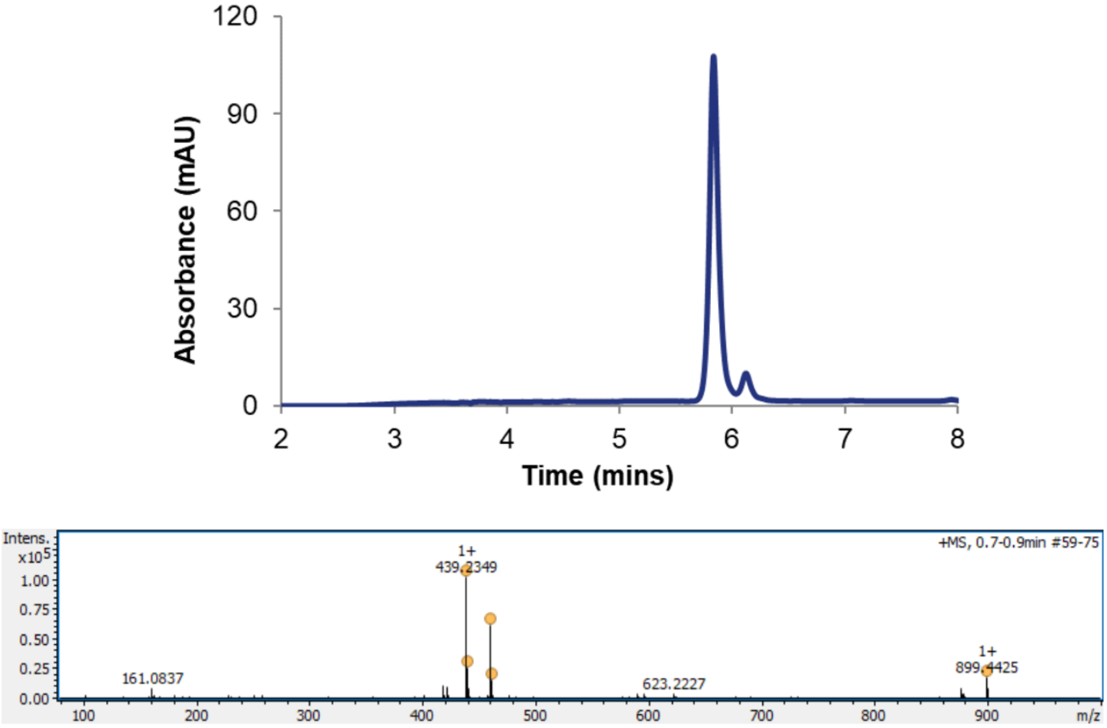


**Figure S52** - Analytical HPLC trace and ESI MS for purified product **31**; analytical gradient 5-60% B over 10 min, 280 nm. Calculated mass $[M+H]^+$: 439.24; observed mass $[M+H]^+$: 439.23.

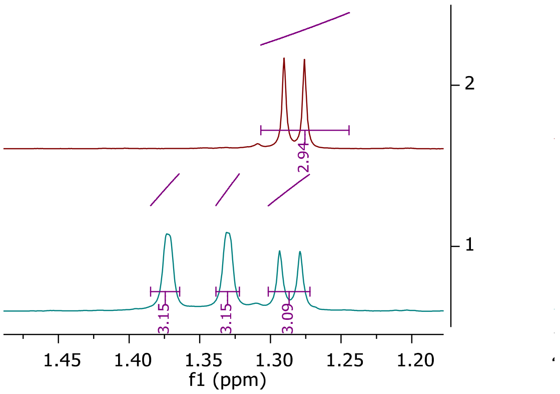


**Figure S53** - NMR overlay of the starting alkene (**29**, red) and the hydrofluorinated product (**31**, green). The right expansion shows the disappearance of the alkene protons at 4.75. The left expansion shows the appearance of two methyl groups, showing fluorination occurred on the internal carbon of the alkene.

## Dual modification of model peptide 26

A mixture of model peptide **20** (2 mg, 1.36 μmol) and isoprenol (**2**, 47 mg, 0.545 mmol) was subjected to the general protocol B and analysed using semi-preparative HPLC (5-60% B over 30 minutes). The products were isolated and analyzed by ESI MS – proposed structures are shown below, no further characterization was attempted.

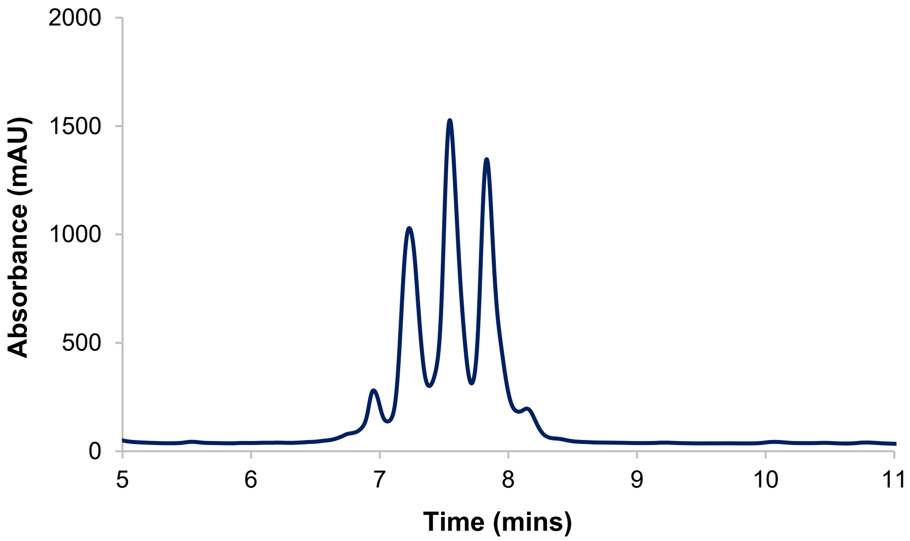


**Figure S54** – Analytical HPLC trace (crude reaction mixture) for the dual modification of peptide **26** with isoprenol **2**; 5-60% over 10 mins, 210 nm.

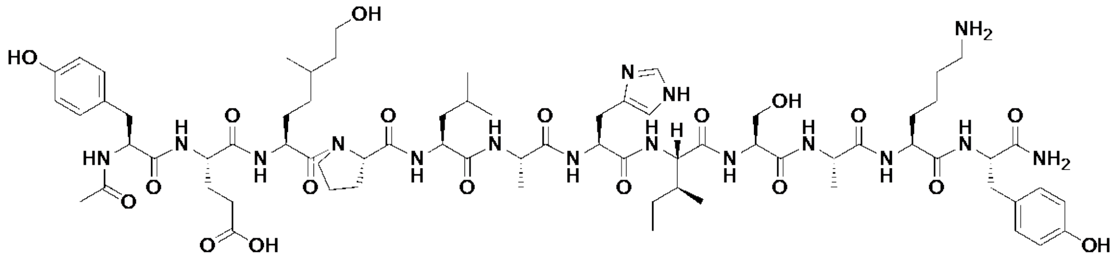


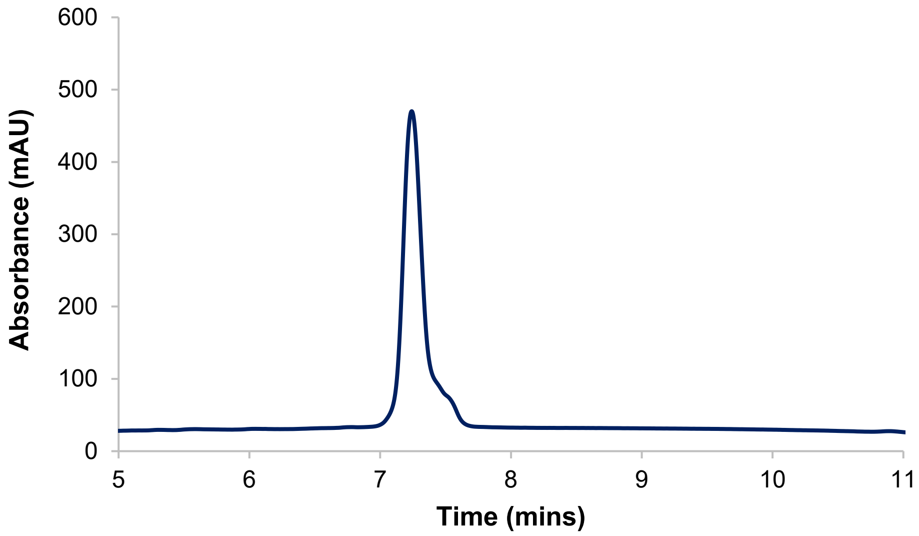


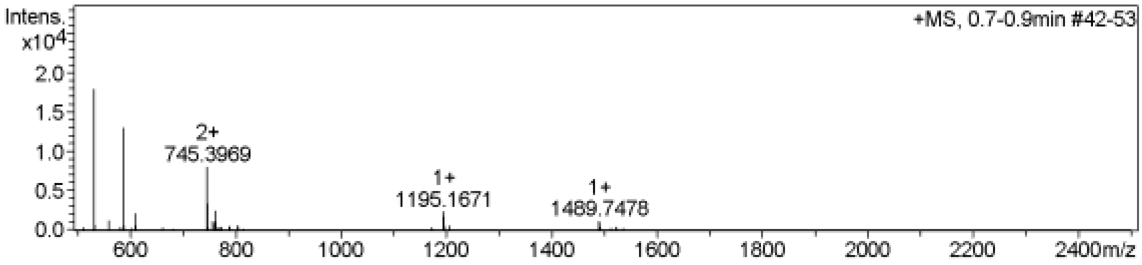


**Figure S55** - Analytical HPLC trace and ESI MS of isolated peak at 7.3 min from the reaction of Ac-YECPLAHISCKY-$NH_2$ (**26**) conjugated with isoprenol (**2**). Analytical gradient 5-60% B over 10 min, 210 nm. Calculated mass $[M+H]^+$: 1489.80, $[M+2H]^{2+}$: 745.40; observed mass $[M+H]^+$: 1489.75, $[M+2H]^{2+}$: 745.40.

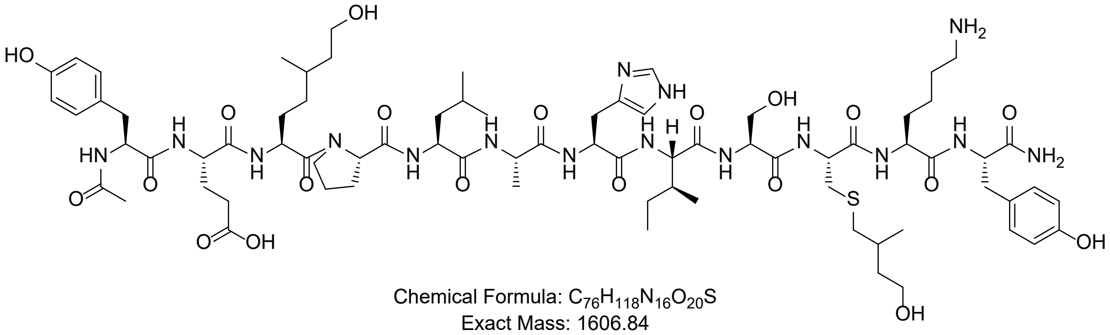


Chemical Formula: $C_{76}H_{118}N_{16}O_{20}S$
Exact Mass: 1606.84
Molecular Weight: 1607.93

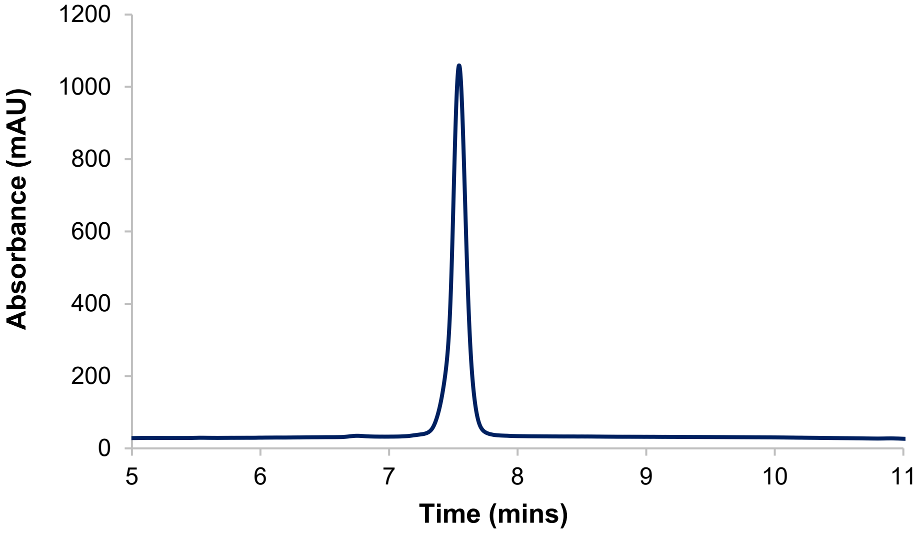


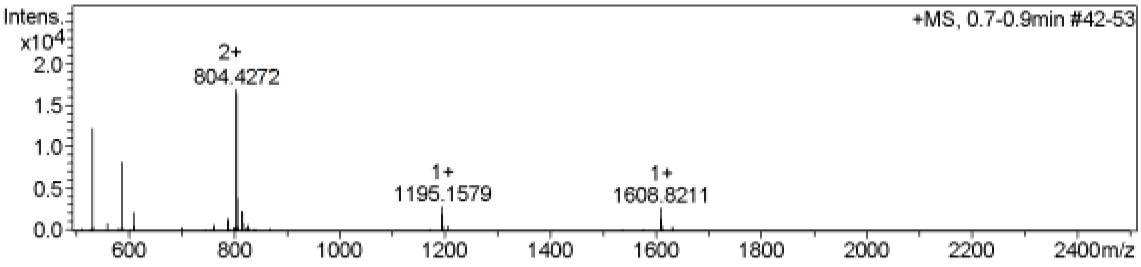


**Figure S56** - Analytical HPLC trace and ESI MS of isolated peak at 7.6 min from the reaction of Ac-YECPLAHISCKY-$NH_2$ (**26**) conjugated with isoprenol (**2**). Analytical gradient 5-60% B over 10 min, 210 nm. Calculated mass $[M+H]^+$: 1608.84, $[M+2H]^{2+}$: 804.42; observed mass $[M+H]^+$: 1608.82, $[M+2H]^{2+}$: 804.43.

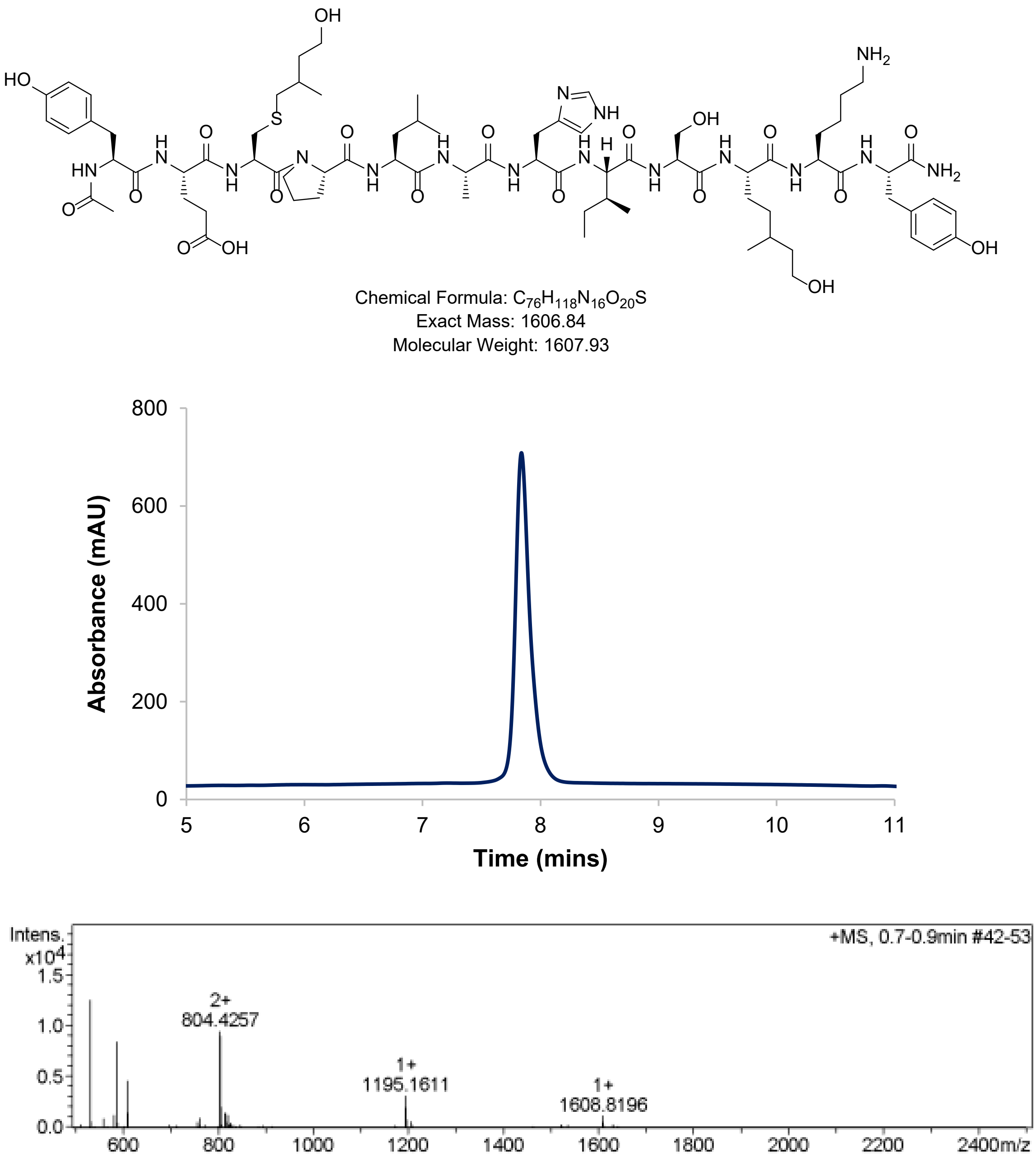


**Figure S57** - Analytical HPLC trace and ESI MS of isolated peak at 7.9 min from the reaction of Ac-YECPLAHISCKY-$NH_2$ (**26**) conjugated with isoprenol (**2**). Analytical gradient 5-60% B over 10 min, 210 nm. Calculated mass $[M+H]^+$: 1608.84, $[M+2H]^{2+}$: 804.42; observed mass $[M+H]^+$: 1608.82, $[M+2H]^{2+}$: 804.43.

## Modification of ubiquitin

### Modification of Ub K48C (32) with *N,N,N*-3-tetramethylbut-3-en-1-aminium iodide (11)

Ubiquitin K48C mutant protein **32** was recombinantly expressed and purified as described in Garner *et al.* 2011 Biochemistry[10] with SDS PAGE analysis confirming ~99% purity (not shown).

H-MQIFVKTLTGKTITLEVESSDTIDNVKSKIQDKEGIPPDQQRLIFAGCQL
EDGRTLSDYNIQKESTLHLVLRLRGG-OH

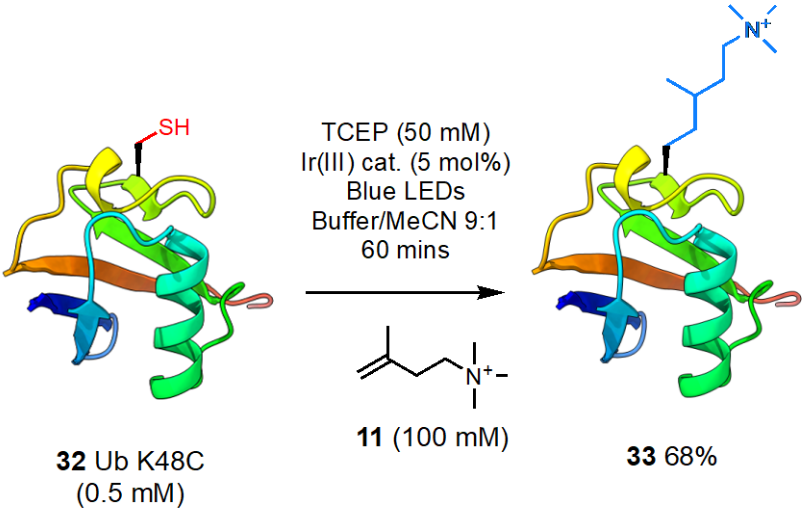


Product **33** was synthesised following the general protocol C (60 mins), using K48C Ub (**32**, 2 mg, 0.234 μmol) and **11** (12 mg, 47 μmol). After analysis the remainder of material (0.222 μmol) was purified using semi-preparative HPLC (25-40% B over 30 minutes) to yield the desired conjugated protein (**33**, 1.3 mg, 0.151 μmol, 68% yield).

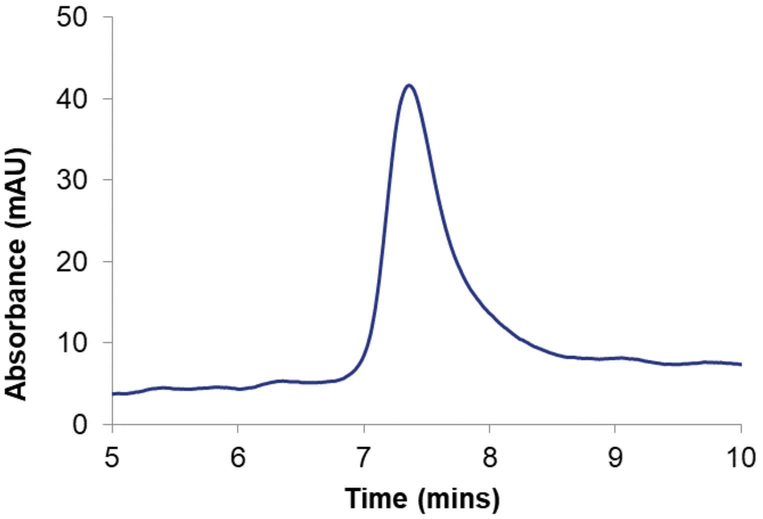


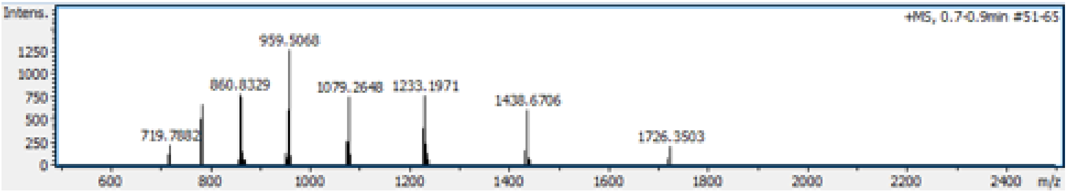


**Figure S58** - Analytical HPLC trace and ESI MS for purified product **33**; analytical gradient 25-35% B over 10 min, 210 nm. Calculated mass $[M+5H]^{5+}$: 1725.74, $[M+6H]^{6+}$: 1438.29, $[M+7H]^{7+}$: 1232.96, $[M+8H]^{8+}$: 1078.97, $[M+9H]^{9+}$: 959.19, $[M+10H]^{10+}$: 863.27. Observed mass $[M+5H]^{5+}$ : 1726.39, $[M+6H]^{6+}$: 1438.67, $[M+7H]^{7+}$: 1233.20, $[M+8H]^{8+}$: 1079.26, $[M+9H]^{9+}$: 959.51, $[M+10H]^{10+}$: 860.83.

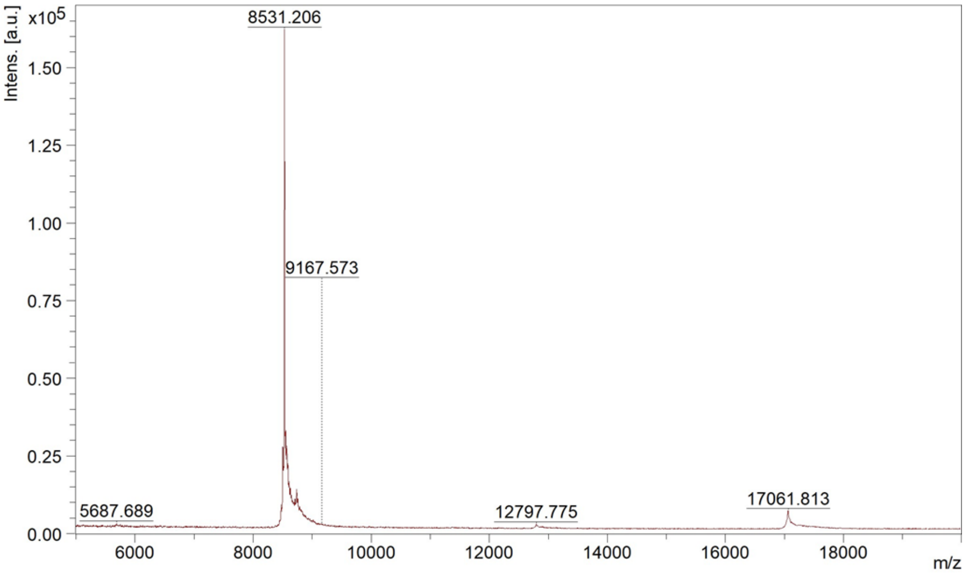

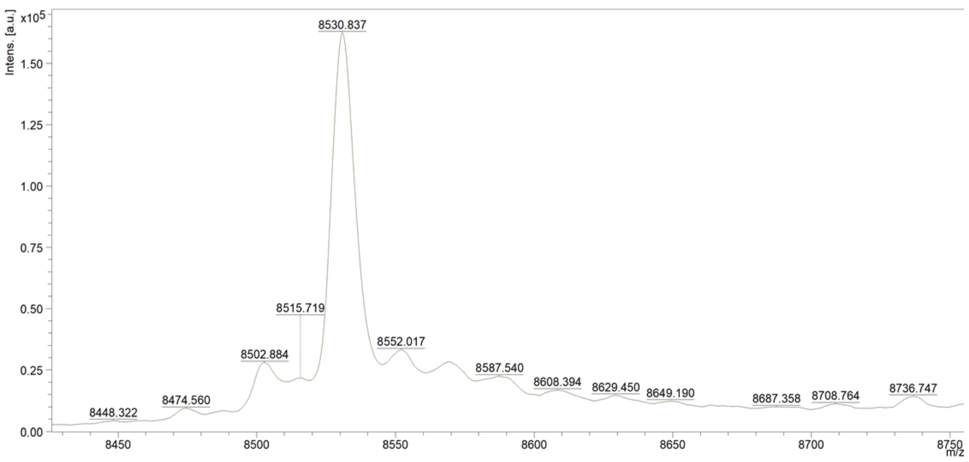


**Figure S59** – Maldi-autoflex mass spec for Ub K48C **32** prepared with 64% sinapinic acid; Calculated mass $[M+H]^+$ : 8531.65; observed mass $[M+H]^+$ :8530.84.

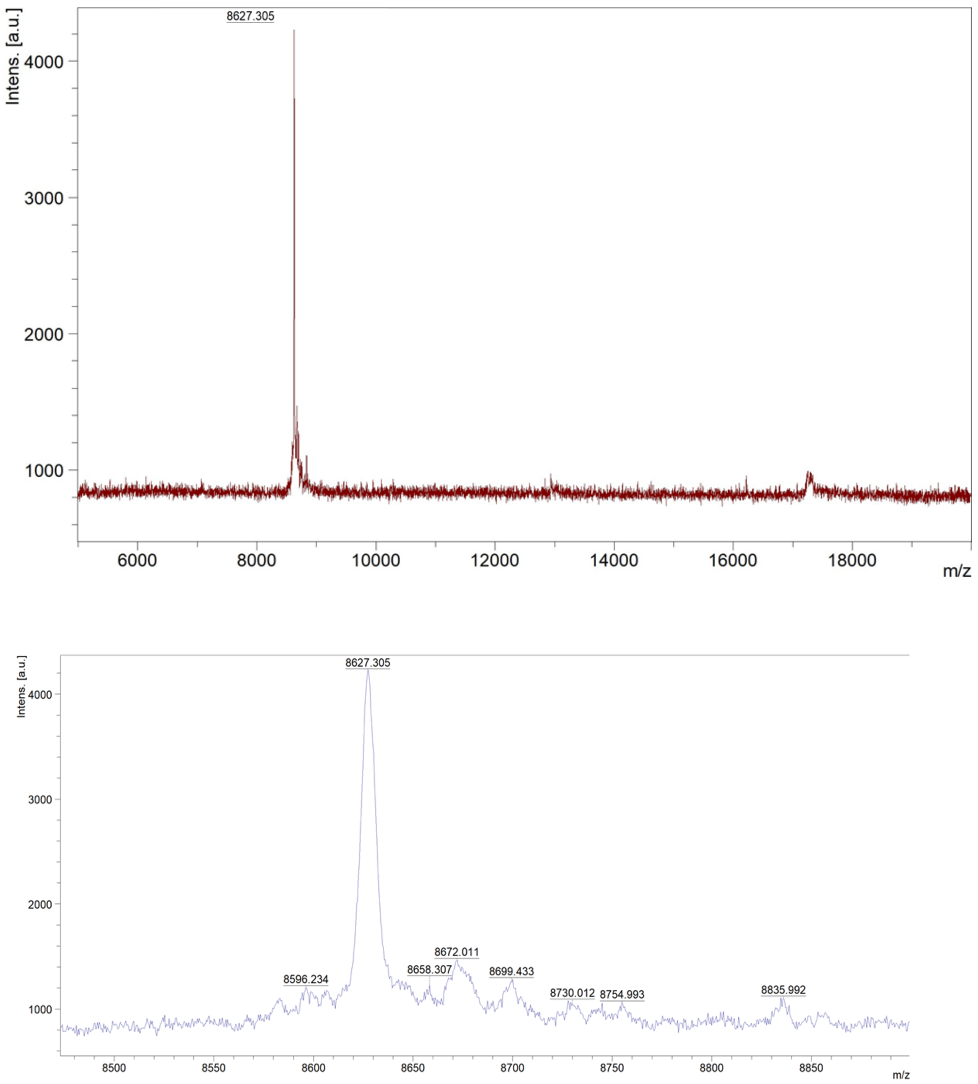


**Figure S60** – Maldi-autoflex mass spec for product **33** prepared with 64% sinapinic acid; Calculated mass $[M+H]^+$ : 8627.72; observed mass $[M+H]^+$ :8627.31.

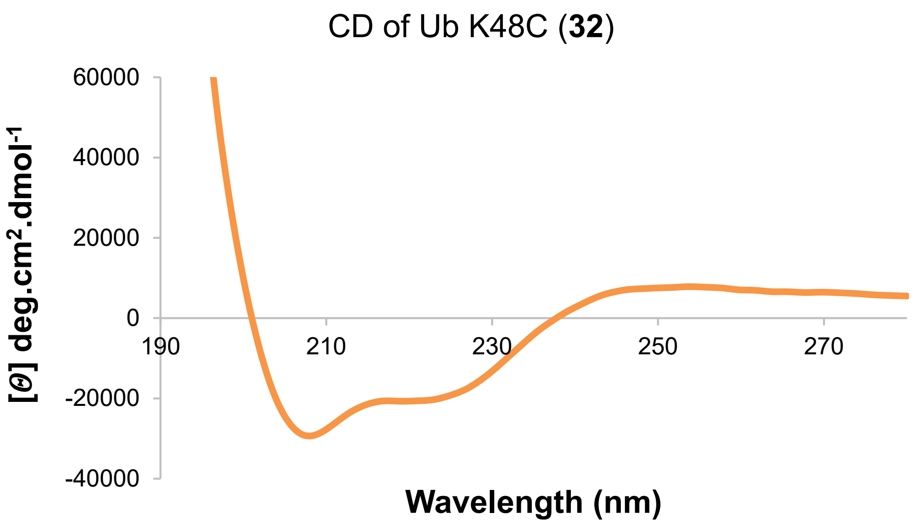


**Figure S61** – Circular dichroism of Ub K48C (**32**); folding buffer: 25 mM $Na_2PO_4$, 100 mM NaCl, pH 7.0.

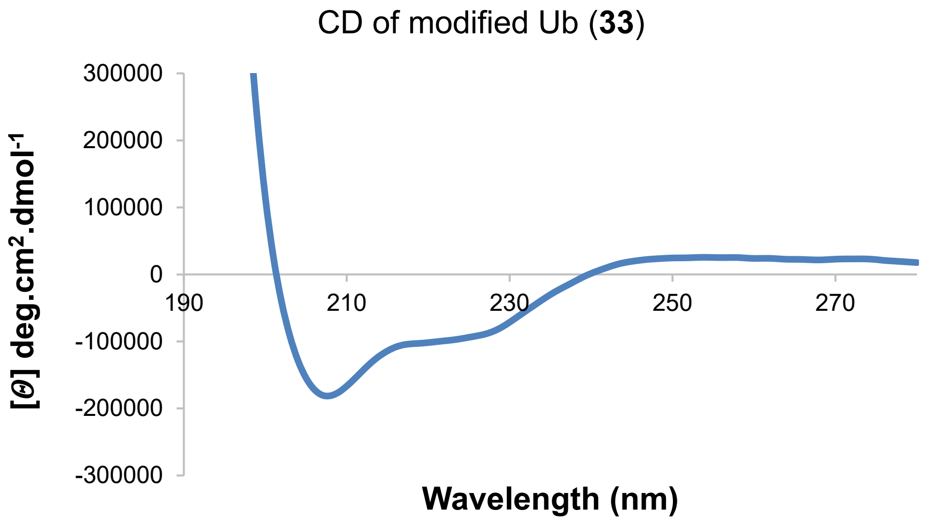


**Figure S62** – Circular dichroism of product **33;** folding buffer: 25 mM $Na_2PO_4$, 100 mM NaCl, pH 7.0.

**Modification of Ub K48C (32) with biotin modified 3-methyl-3-butene-1-amine (12)**

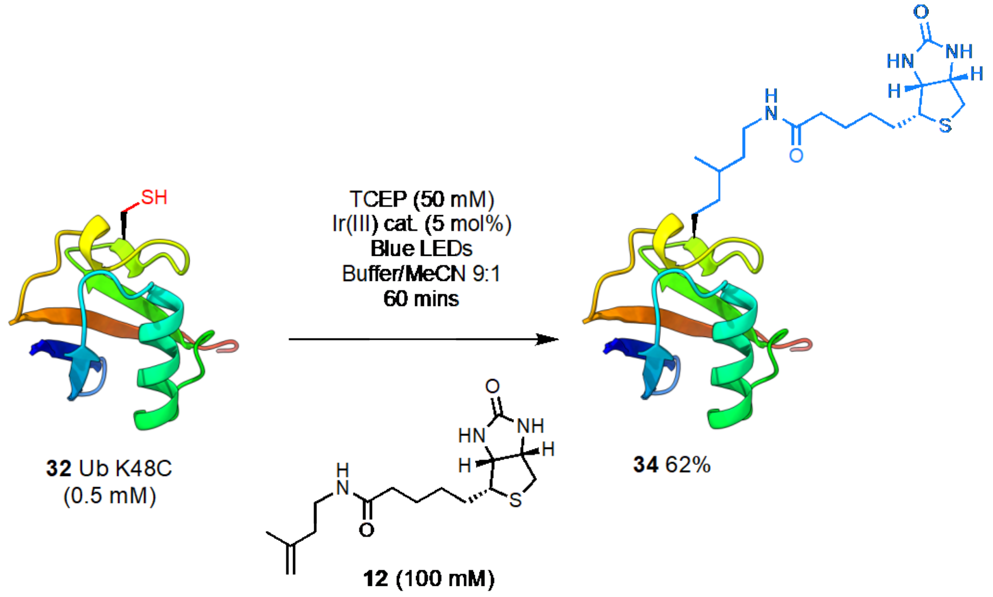


Product **34** was synthesised following the general conjugation procedure C (60 mins), using K48C Ub **32** (2 mg, 0.234 µmol) and **12** (15 mg, 47 µmol). After analysis the remainder of material (0.222 µmol) was purified using semi-preparative HPLC (25-40% B over 30 minutes) to yield the desired conjugated protein (**34**, 1.2 mg, 0.136 µmol, 62% yield).

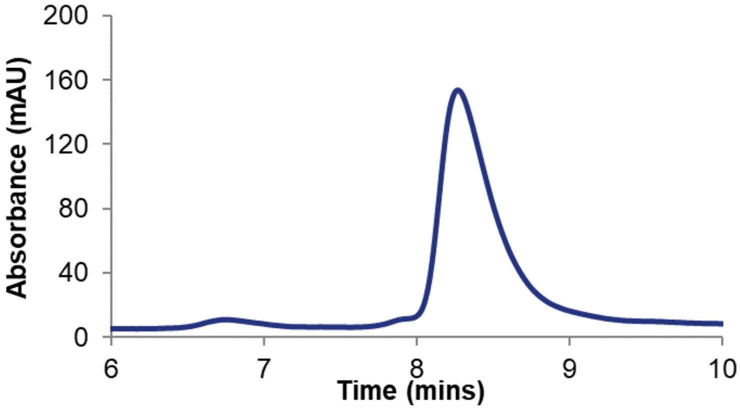


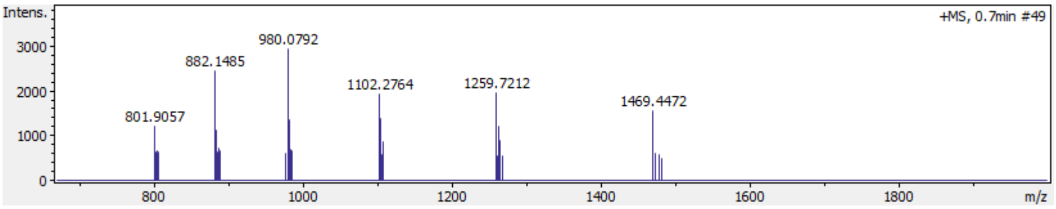

**Figure S63** - Analytical HPLC trace and ESI MS for purified product **34**; analytical gradient 25-35% B over 10 min, 210 nm. Calculated mass $[M+6H]^{6+}$: 1468.62, $[M+7H]^{7+}$: 1258.96, $[M+8H]^{8+}$: 1101.72, $[M+9H]^{9+}$: 979.42, $[M+10H]^{10+}$: 881.58, $[M+11H]^{11+}$: 801.52. Observed mass $[M+6H]^{6+}$: 1469.45, $[M+7H]^{7+}$: 1259.72, $[M+8H]^{8+}$: 1102.28, $[M+9H]^{9+}$: 980.08, $[M+10H]^{10+}$: 882.15, $[M+11H]^{11+}$: 801.91.

## Reaction optimisation to minimise by-product formation

### General method

To peptide dissolved in 10% acetonitrile (MeCN) in CB to a concentration of 1.0 mM was added a solution of TCEP (0.5 M stock solution in CB, pH adjusted to 8, 10 eq.), stabilised alkene compound (pure, 200-1000 eq.) and $(Ir[dF(CF_3)ppy]_2(dtbpy))PF_6$ (1 mM stock solution in MeCN, 0.05 eq.). The reaction mixture was diluted to a final peptide concentration of 0.5 mM and the pH of the reaction mixture was confirmed to be 7.8 - 8.2, this was adjusted as required by addition of either 10 M NaOH or 6 M HCl (care must be taken to not allow the pH to rise above 9). The reaction vessel was then placed into PhotoRedOx Box and directly analysed by analytical HPLC.

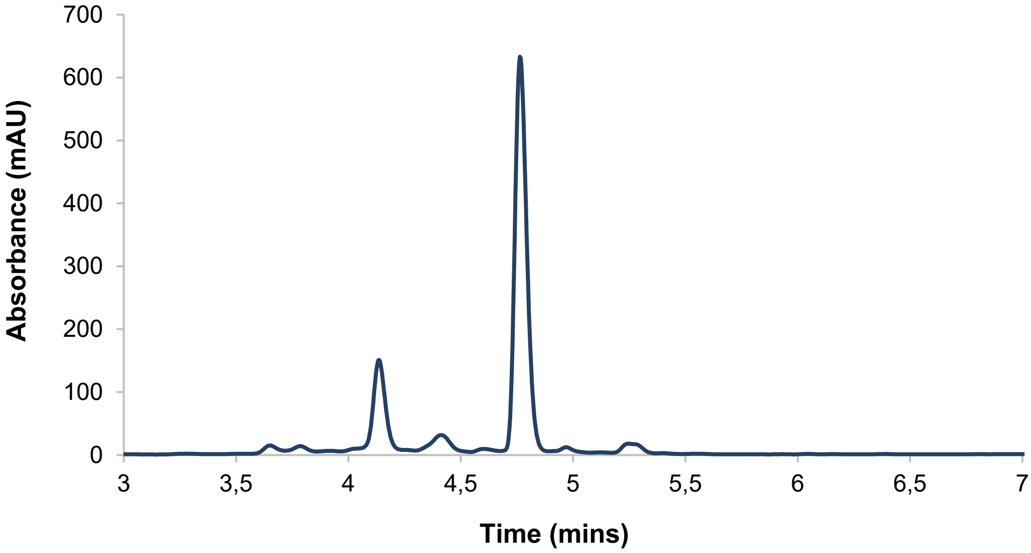


**Figure S64 -** Analytical HPLC trace of reaction mixture for Ac-CWHISKEY-$NH_2$ (**1**) modified with 200 eq. isoprenol (**2**) for 2 min in the PhotoRedOx box. Analytical gradient 15-60% B over 10 min, 210 nm (alanine by-product observed at 4.2 min).

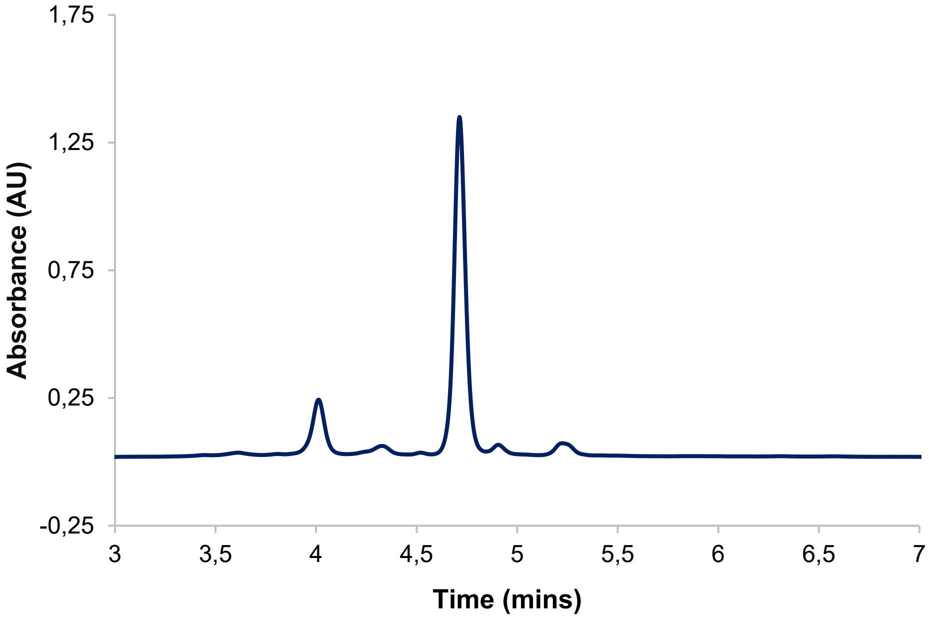


**Figure S65** - Analytical HPLC trace of reaction mixture for Ac-CWHISKEY-$NH_2$ (**1**) modified with 500 eq. isoprenol (**2**) for 2 min in the PhotoRedOx box. Analytical gradient 15-60% B over 10 min, 210 nm (alanine by-product observed at 4.2 min).

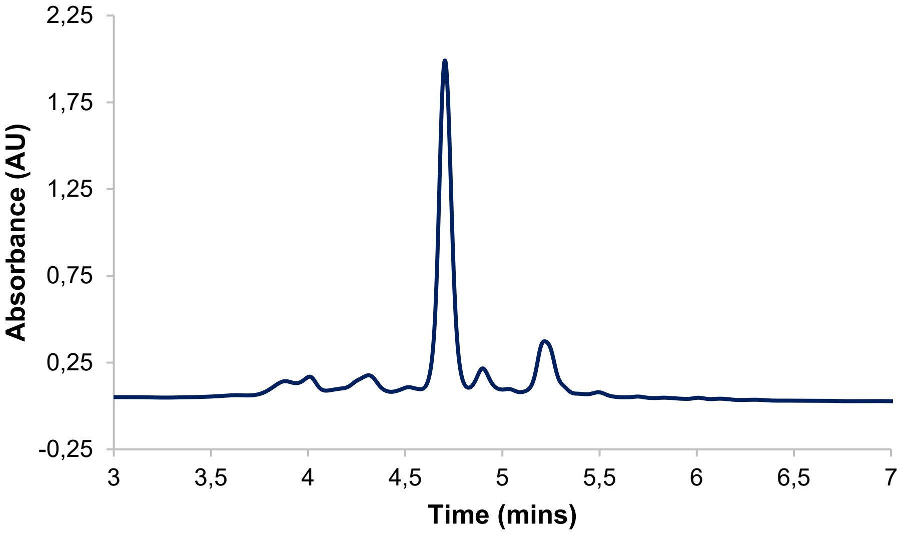


**Figure S66** - Analytical HPLC trace of reaction mixture for Ac-CWHISKEY-$NH_2$ (**1**) modified with 700 eq. isoprenol (**2**) for 2 min in the PhotoRedOx box. Analytical gradient 15-60% B over 10 min, 210 nm.

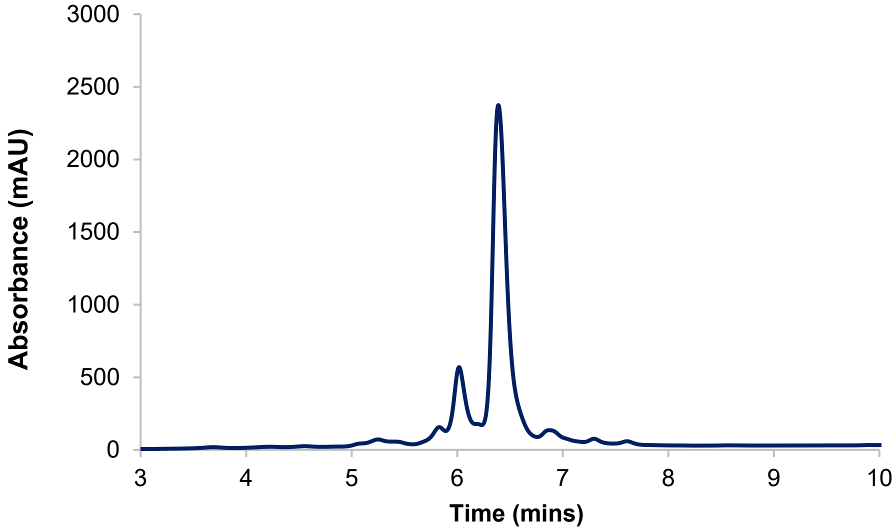


**Figure S67** - Analytical HPLC trace of reaction mixture for Ac-YEPLACHISKY-$NH_2$ (**8**) modified with 200 eq. isoprenol (**2**) for 5 min in the PhotoRedOx Box. Analytical gradient 15-45% B over 10 min, 210 nm.

**Table S6 –** Exploration of probe concentration on conversion of peptide **6** to product **15** (all reactions carried out using the PhotoRedOx box).

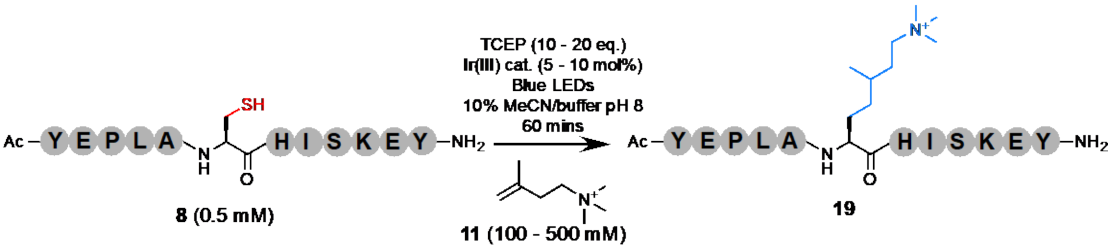


| Entry | Peptide [mM] | Probe [eq.] | Ir [%] | TCEP [eq.] | Conversion [%] | Reaction time |
|---|---|---|---|---|---|---|
| 23 | 0.5 | 200 | 5 | 10 | 23 | 5 min |
| 24 | 0.5 | 200 | 10 | 10 | 36 | 5 min |
| 25 | 0.5 | 200 | 5 | 10 | 35 | 10 min |
| 26 | 0.5 | 200 | 10 | 10 | 47 | 10 min |
| 27 | 0.5 | 200 | 5 | 10 | 54 | 15 min |
| 28 | 0.5 | 200 | 10 | 10 | 58 | 15 min |
| 29 | 0.5 | 200 | 5 | 10 | 62 | 20 min |
| 30 | 0.5 | 200 | 10 | 10 | 64 | 20 min |
| 31 | 0.5 | 200 | 5 | 10 | 74 | 30 min |
| 32 | 0.5 | 200 | 10 | 10 | 74 | 30 min |

| | | | | | | |
|---|---|---|---|---|---|---|
| 33 | 0.5 | 200 | 5 | 10 | 82 [78 isolated] | 1.0 h |
| 34 | 0.5 | 200 | 10 | 10 | 81 | 1.0 h |
| 35 | 0.5 | 500 | 5 | 10 | 67 | 30 min |
| 36 | 0.5 | 500 | 5 | 10 | 83 | 1.0 h |
| 37 | 0.5 | 700 | 5 | 10 | 24 | 5 min |
| 38 | 0.5 | 700 | 5 | 10 | 37 | 10 min |
| 39 | 0.5 | 700 | 5 | 10 | 54 | 15 min |
| 40 | 0.5 | 700 | 5 | 10 | 61 | 20 min |
| 41 | 0.5 | 700 | 5 | 10 | 70 | 30 min |
| 42 | 0.5 | 700 | 5 | 10 | 79 | 1.0 h |
| 43 | 0.5 | 700 | 5 | 20 | 78 | 1.0 h |
| 44 | 0.5 | 1000 | 5 | 10 | 41 | 10 min |
| 45 | 0.5 | 1000 | 5 | 10 | 74 | 30 min |
| 46 | 0.5 | 1000 | 5 | 10 | 90 | 1.0 h |

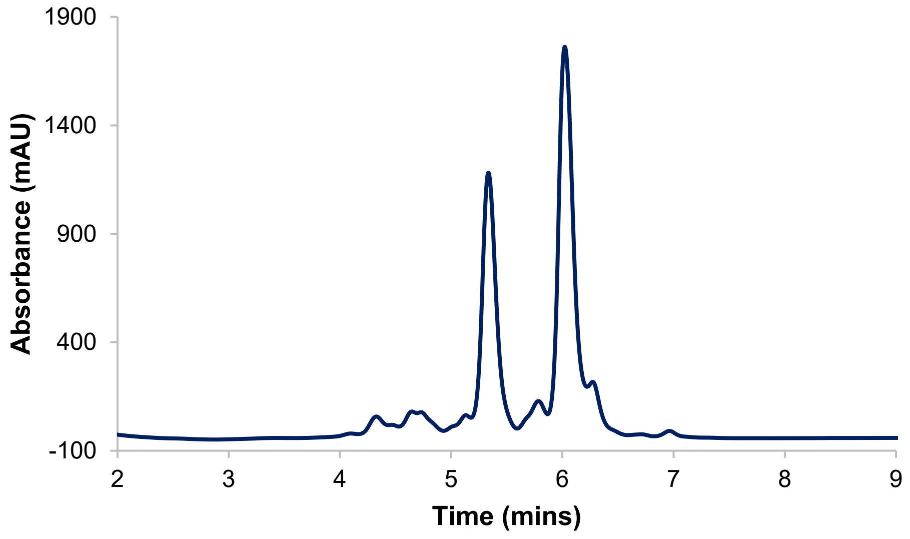


**Figure S68** - Analytical HPLC trace of reaction mixture for Ac-YEPLACHISKY-$NH_2$ (**8**) modified with 1000 eq. *N,N,N*,3-tetramethylbut-3-en-1-aminium (**11**) for 10 min in the PhotoRedOx Box Analytical gradient 15-45% B over 10 min, 210 nm.

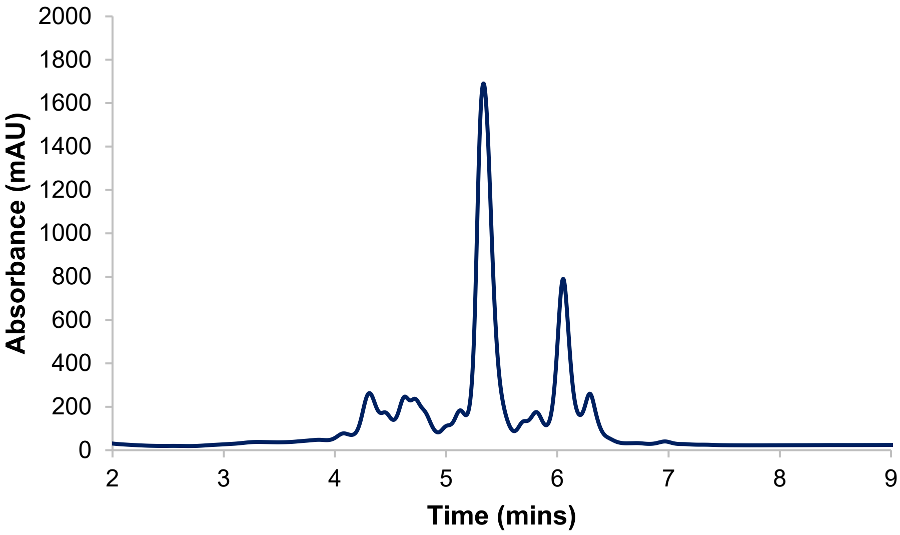


**Figure S69** - Analytical HPLC trace of reaction mixture for Ac-YEPLACHISKY-$NH_2$ (**8**) modified with 1000 eq. *N,N,N*,3-tetramethylbut-3-en-1-aminium (**11**) for 30 min in the PhotoRedOx Box Analytical gradient 15-45% B over 10 min, 210 nm.

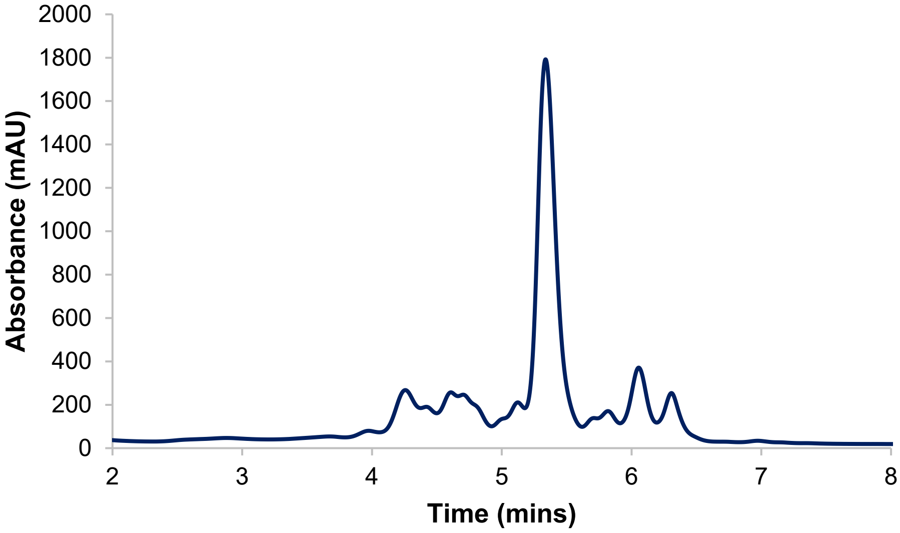


**Figure S70** - Analytical HPLC trace of reaction mixture for Ac-YEPLACHISKY-$NH_2$ (**8**) modified with 1000 eq. *N,N,N*,3-tetramethylbut-3-en-1-aminium (**11**) for 60 min in the PhotoRedOx Box Analytical gradient 15-45% B over 60 min, 210 nm.

## Modification of histone H4

### H4 K20C (35) production - expression in BL21

A H4 K20C-encoding pET plasmid was transformed into electrocompetent BL21 DE3. 6 x 500ml cultures were grown to $OD_{600}$ = 0.6 at 37 °C and expression was induced with the addition of 0.5 mM IPTG for 3 hours. H4 K20C is expressed as inclusion bodies.

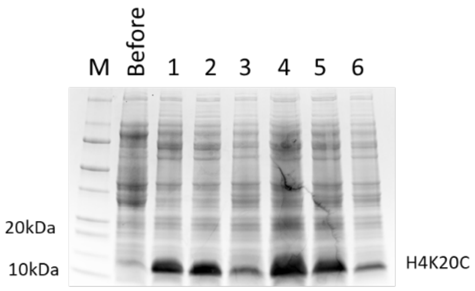


**Figure S71 –** SDS-PAGE gel of histone H4 K20C (**35**) expression following IPTG induction.

Cells were lysed in lysis buffer (50 mM Tris, 100 mM NaCl, 1 mM EDTA, 1 mM 2-mercaptoethanol, 1 mM 2-mercaptoethanol, 1 mg/mL lysozyme, pH 7.6) by sonication at 70% amplitude for 10 minutes (30 seconds on, 30 seconds off). Lysate was centrifuged at 4 ºC for 30 minutes at 15,000 RPM, supernatant was decanted and the pellet resuspended in lysis buffer + 1% Triton X and spun again at 15,000 RPM 4 ºC for 15 mins. The pellet was washed once more in lysis buffer without triton and spun 15,000 RPM at 4 ºC for 15 minutes.

The inclusion body pellet was then resuspended in buffer containing 6 M guanidine, 20 mM Tris, 1 mM EDTA, 100 mM NaCl, 1 mM DTT and left to mix in the cold room for 2 hours before being centrifuged for 30 min 15,000 RPM. The supernatant was collected, acidified with TFA, filtered, and injected onto analytical and preparative HPLC systems.

Analytical HPLC was performed on Agilent Infinity II 1260 series instrument using a C18 Zorbax column (5 µm, 4.6 x 150 mm) at a flow rate of 1 ml/min. All runs used 0.1 % TFA (trifluoroacetic acid) in water (solvent A) and 0.1 % TFA in acetonitrile (solvent B). 20-70% B gradient.

Preparative HPLC was performed on an Agilent 1260 Preparative system and purifications were carried out on a C18 Zorbax column (5 µm; 21.2 x 100 mm) at a flow rate of 20 mL/min. All runs used 0.1 % TFA (trifluoroacetic acid) in water (solvent A) and 0.1 % TFA in acetonitrile (solvent B). 20-70% B gradient.

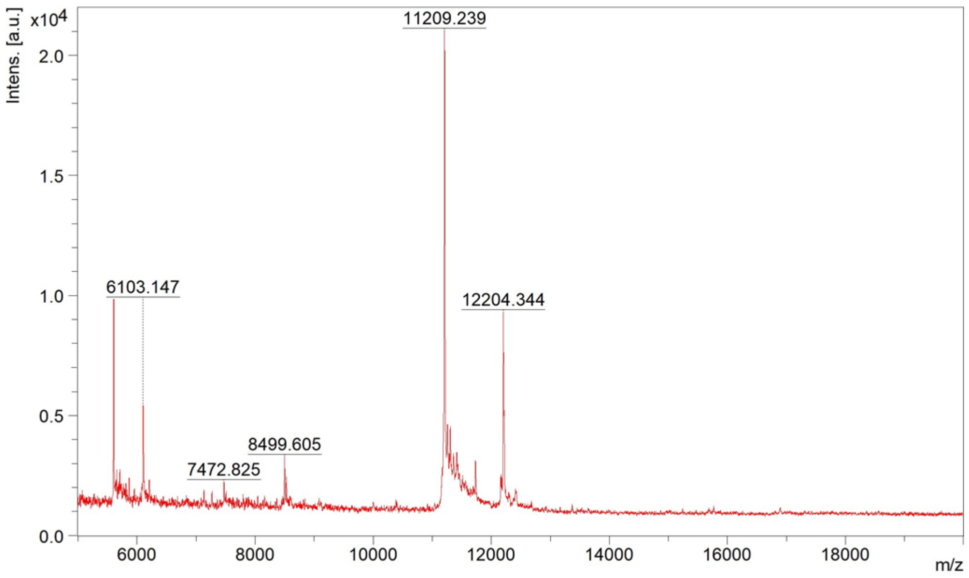


**Figure S72** – Maldi-autoflex mass spec for histone H4 K20C (**35**) protein prepared with 64% sinapinic acid; Calculated mass – 11,210.99 $[M+H]^+$; observed mass – 11,209.24 $[M+H]^+$.

### Modification of histone H4 K20C (35) with *N,N,N*-3-tetramethylbut-3-en-1-aminium iodide (11)

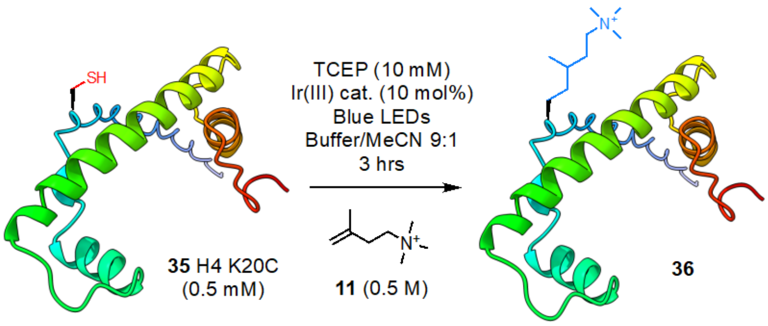


H-SGRGKGGKGLGKGGAKRHRCVLRDNIQGITKPAIRRLARRGGVKRISGL
IYEETRGVLKVFLENVIRDAVTYTEHAKRKTVTAMDVVYALKRQGRTLYGFG
G-OH

Product **36** was synthesised following general conjugation procedure D. To protein dissolved in 10% acetonitrile (MeCN) in CB to a concentration of 1.0 mM was added a solution of TCEP (0.5 M stock solution in CB, pH adjusted to 8, 20 eq.), stabilised alkene compound (1000 eq.) and (Ir[dF($CF_3$)ppy]$_2$(dtbpy))$PF_6$ (1 mM stock solution in MeCN, 0.1 eq.). The reaction mixture was diluted to a final peptide concentration of 0.5 mM and the pH of the reaction mixture was confirmed to be around 8 (7.8-8.2), this was adjusted as required by addition of either 10 M NaOH or 6 M HCl (care must be taken to not allow the pH to rise above 9). The reaction vessel was then placed into blue LEDs and left for 3 hours to ensure complete conversion. Reaction mixture was directly sent for Maldi-autoflex mass after reaction with simple desalting treatment (preparative HPLC/SEC/dialysis).

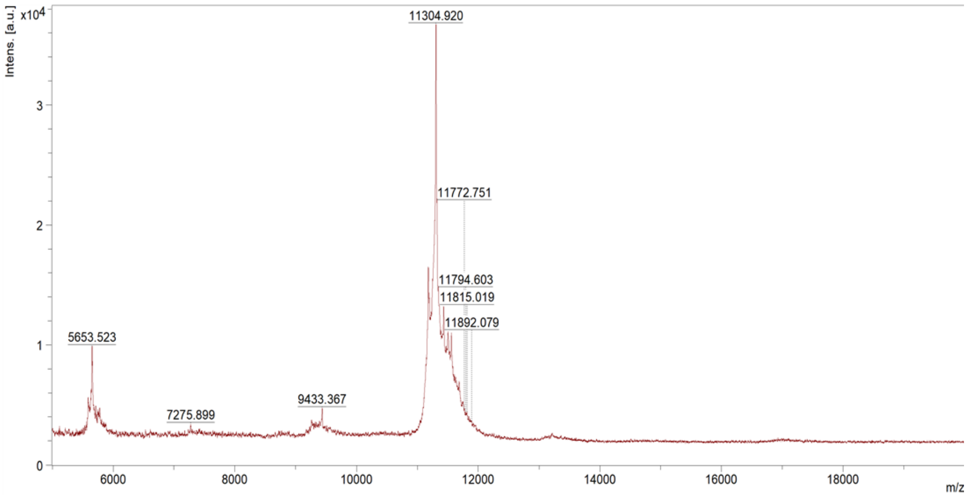


**Figure S73** – Maldi-autoflex mass spec for crude histone H4 K20C (**35**) reaction mixture modified with 1000 eq. *N,N*-3-trimethylbut-3-en-1-amine (**11**), 20 eq. TCEP and 10 mol% Ir cat. for 3 h under blue LEDs and prepared with 64% sinapinic acid. Calculated mass – 11,305.40 $[M+H]^+$; observed mass – 11,304.90 $[M+H]^+$.

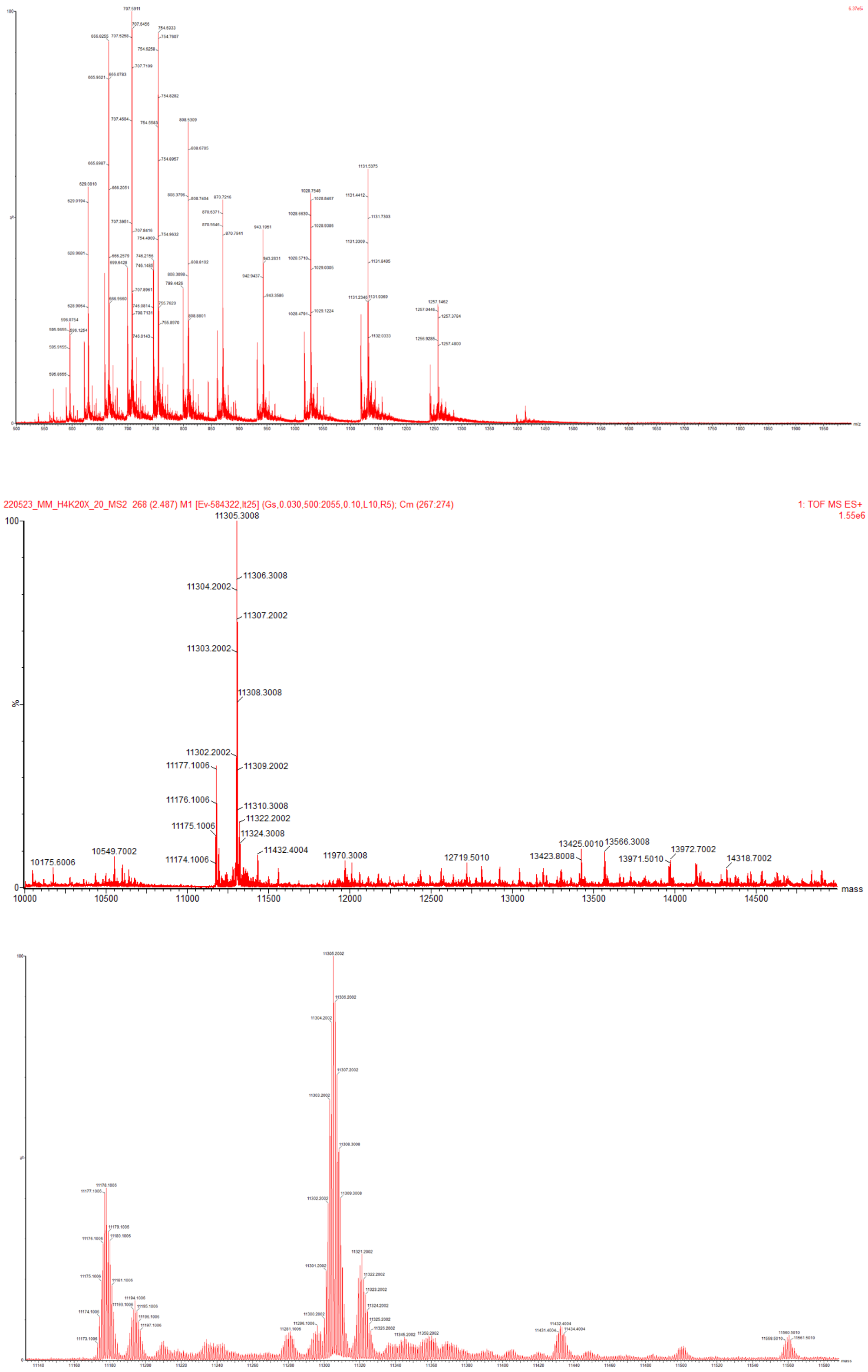


**Figure S74** – ESI MS for crude histone H4 K20C (**35**) reaction mixture modified with 1000 eq. *N,N*-3-trimethylbut-3-en-1-amine (**11**), 20 eq. TCEP and 10 mol% Ir cat. for 5 h under blue LEDs and prepared with 64% sinapinic acid. Calculated mass – 11,305.4 $[M+H]^+$; observed mass – 11,305.3 $[M+H]^+$.

## Desulfurization of H4 K20C (37)

Histone H4 K20C (**35,** 2 mg, 0.178 µmol) was desulfurized at a concentration of 0.5 mM using 200 eq. of TCEP, in the presence of 10 mol% Ir cat. for 5 hrs under irradiation from blue LEDs.

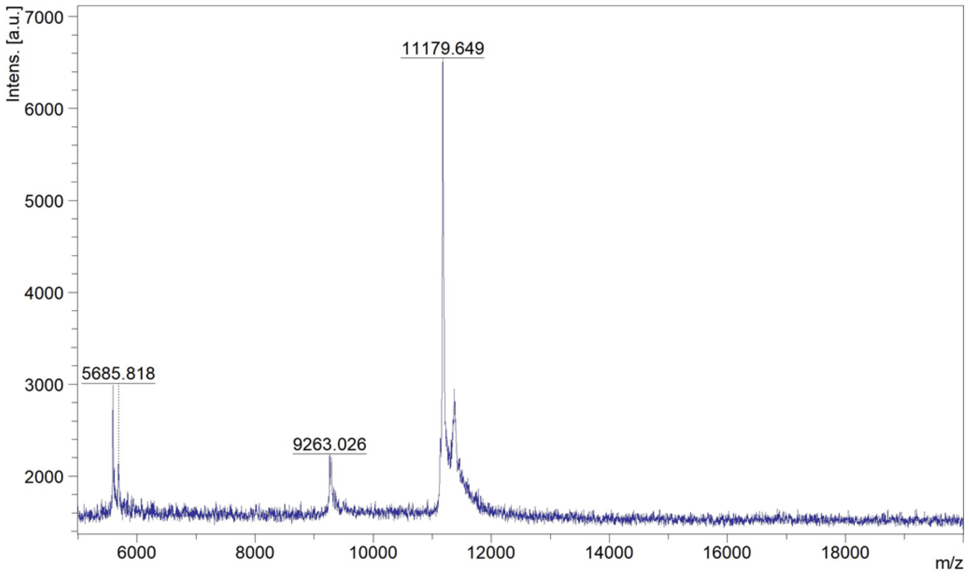


**Figure S75** – Maldi-autoflex mass spec for desulfurized histone H4 K20C (**35**), prepared with 64% sinapinic acid. Calculated mass – 11,179.92 [M+H]$^+$; observed mass – 11,179.65 [M+H]$^+$.

## Alkylation of H4 K20C (38)

Histone H4 K20C was modified as previously described to give product **38**.[11]

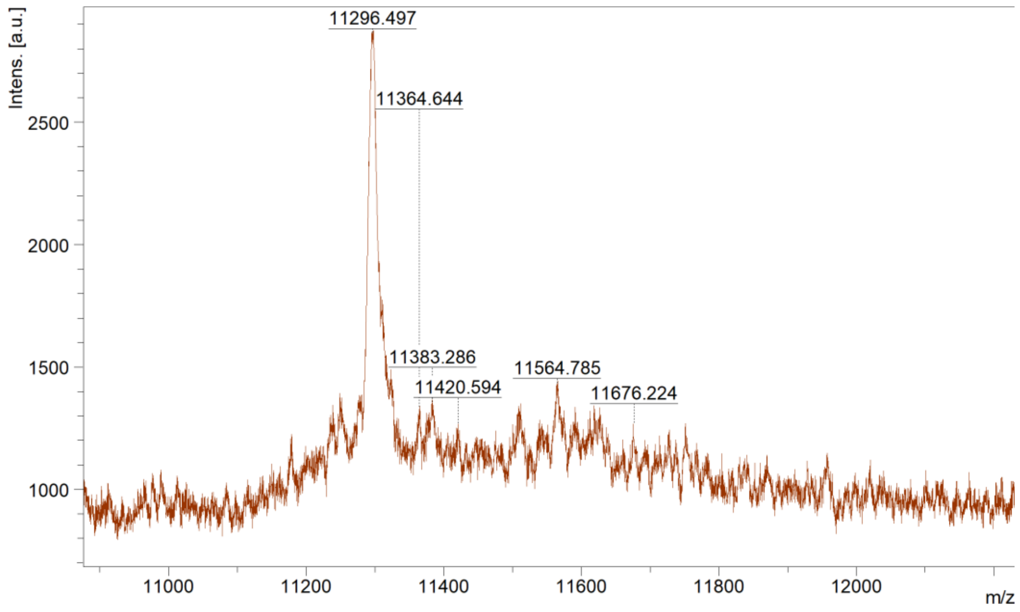


**Figure S76** – Maldi-autoflex mass spec for product **38**, prepared with 64% sinapinic acid. Calculated mass – 11,298.09 $[M+H]^+$; observed mass – 11,296.50 $[M+H]^+$.

## Western Blot

Proteins (0.9 ug or 1.8 ug total protein) were analysed by Western blot upon SDS-PAGE (4-15% stain-free Mini-PROTEAN TGX Gels, Bio-Rad). Gels for loading controls were stained with Coomassie for 15 mins, followed by washes in water. Proteins were then transferred onto a PVDF membrane (activated in methol for 60 secs), using a trans-blot turbo (Bio-Rad) with standard settings for mixed molecular weight proteins. The membrane was subsequently blocked in 3% milk TBS-T for one hour at room temperature before incubation with Anti-histone H4 (trimethyl K20) antibody (Abcam ab227884) according to manufacturer's instructions (1:1000, overnight in cold room with TBS-T containing 3% milk). The membrane was washed three times for 10 min in TBS-T before incubation with secondary antibody (Goat anti-rabbit-HRP, Bio-Rad #1706515, 1:2000 TBS-T containing 3% milk) for one hour room temperature. The membrane was washed again three times 10 min before ECL reagent was added and the blots were imaged on a ChemiDoc imaging system (Bio-Rad).

Typically, an exposure time of 1-2 secs was used for low exposure and 10 seconds for high exposure.

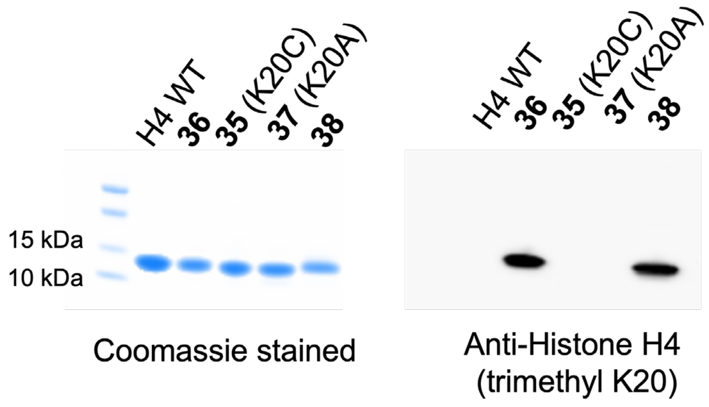


**Figure S77 –** Western Blot for proteins histone H4 WT, **36**, **35**, **37**, **38** using anti-histone H4 trimethyl K20 antibody.

## DFT on peptide radicals to compute reaction free energies

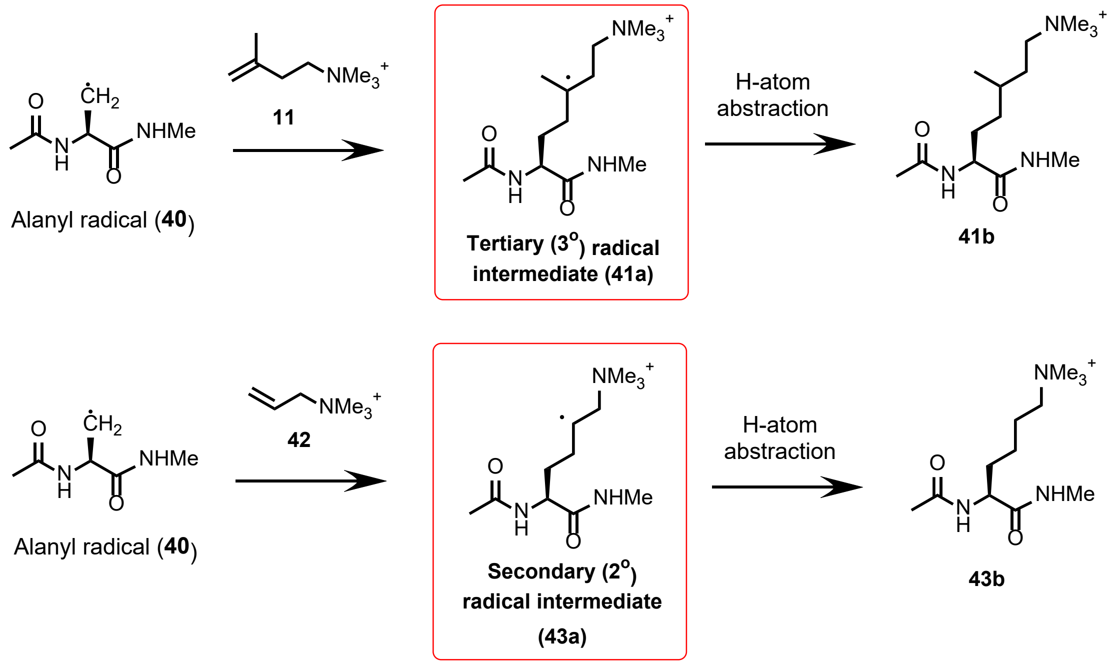


**Figure S78** – Scheme illustrating the tertiary radical produced when trapping alanyl radical (**40**) with isopenyl **11** and the secondary radical produced when trapping alanyl radical (**40**) with allylamine **42**.

### Results in gas phase

The optimised DFT structures in vacuum are shown in Figures S79 and S80, and the reaction energetics in the gas phase are shown in Table 7.

Two sets of conformations of the products were found: one set with parallel amide groups (with no H-bond between them) and one set with anti-parallel amide groups (with an H-bond between them). The 2° radical (**43a**) (parallel) and product are lower in energy than the anti-parallel; and the 3° radical (**41a**) (anti-parallel) and product are lower in energy than the parallel.

The lowest energy conformer found for the alanyl radical (**40**) is non-planar with the amide groups anti-parallel. This structure is used for the reaction energetics shown in Table 7.

**Parallel amide groups.** DeltaG for formation of the 3° radical (**41a**) is more favourable than for formation of the 2° radical (**43a**). DeltaG for formation of product **43b** (from 2° radical **43** + H) is more favourable than formation of product **41b**. DeltaG for formation of the product **43b** (from alanyl radical (**40**) + amine **42** + H) is negligibly more favourable than formation of the product **41b**.

**Anti-parallel amide groups.** DeltaG for formation of the 3° radical **41a** and the product **41b** (from alanyl radical (**40**) + **11** + H) is more favourable than formation of product **43a**. DeltaG for formation of product **43b** (from **43a** + H) is more favourable than formation of the 3° radical (**41a**).

**Results in bulk acetonitrile**

Reaction energetics in bulk acetonitrile are shown in Table 8. The results are outlined below.

**Parallel amides**. DeltaG is more favourable for formation of the 2° radical (**43a**) compared to the 3° radical (**41a**), and product **43b** (from 2° radical **43a** + H or from alanyl radical (**40**) + amine **42** + H) compared to product **41b**.

**Anti-parallel amides**. DeltaG is equivalent for formation of the 2° radical (**43a**) and 3° radical (**41a**). DeltaG is more favourable for formation of product **43b** (from 2° radical **43a** + H or from alanyl radical (**40)** + amine **42** + H) compared to the product **41b**.

The structures of the 2° radical **43a** and product **43b** with anti-parallel amides both have the sidechain extended in length away from the amide groups, when in acetonitrile (described by PCM). These structures are displayed in Figure S81. The DFT total energies for relative energies of the structures in acetonitrile: The 2° radical (**43a**) with anti-parallel amides is –0.5 kJ/mol lower in energy than the 2° radical (**43a**) with parallel amides. The 3° radical (**41a**) with parallel amides is 10.7 kJ mol higher in energy than the 3° radical (**41a**) with anti-parallel amides. The product **43b** with anti-parallel amides is 3.8 kJ/mol

higher in energy than product **43b** with parallel amides. The product **41b** with parallel amides is 11.0 kJ/mol higher in energy than the product **41b** with anti-parallel amides.

**Results in bulk water**

Reaction energetics in bulk water are shown in Table 9. The results are outlined below and are as per the results for bulk acetonitrile solvent (above).

**Parallel amides**. DeltaG is more favourable for formation of the 2° radical (**43a**) compared to the 3° radical (**41a**), and product **43b** (from 2° radical (**43a**) + H or from alanyl radical (**40**) + **42** + H) compared to product **41b**.

**Anti-parallel amides**. DeltaG is equivalent for formation of the 2° and 3° radicals (**43b** and **41b**). DeltaG is more favourable for formation of product **43b** (from 2° radical **43a** + H or from alanyl radical (**40**) + **42** + H) compared to product **41b**.

The structures of the 2° radical (**43a**) and product (**43b**) with anti-parallel amides both have the sidechain extended in length away from the amide groups, when in water (described by PCM). These structures are displayed in Figure S81.

The DFT total energies for relative energies of the structures in acetonitrile: The 2° radical (**43a**) with anti-parallel amides is –1.1 kJ/mol lower in energy than the 2° radical (**43a**) with parallel amides. The 3° radical (**41a**) with parallel amides is 10.8 kJ mol higher in energy than the 3° radical (**41a**) with anti-parallel amides. Product **43b** with anti-parallel amides is 3.4 kJ/mol higher in energy than product **43b** with parallel amides. Product **41b** with parallel amides is 11.0 kJ/mol higher in energy than product **41b** with anti-parallel amides.

**Discussion**

The DFT calculations predict that, in the gas phase, the formation of the 3° radical (**41a**) is more favourable than the 2° radical (**43a**), but where the H-atom

abstraction of the more reactive 2° radical (**43a**) is favourable compared to the more stable 3° radical (**41a**) (Table 7).

The formation of the parallel 2° radical (**43a**) is more favourable in MeCN and water (Tables 8 and 9). Formation of the 2° (**43a**) and 3° (**41a**) radicals is equivalent for the anti-parallel amide groups. This may be explained by the conformation in solvent – the sidechain for the 2° radical (**43a**) and product in solvent extends away from the amide. In both bulk solvents, the anti-parallel 2° radical (**43a**) sidechain is extended away from the amide (Figure S81). This may be due to the screening, by the solvent, of the interaction between the amide oxygen atom and the amine at the end of the sidechain. The stabilising of the amine charge by the solvent being more favoured. If the 3° radical (**41a**) sidechain is more flexible than the 2° radical (**43a**) sidechain, then the amine of the 3° radical (**41a**) group may get closer to the amide oxygen atom to form a strong interaction in the 3° radical (**41a**) and product **41b**, and be more energetically favoured than the solvent stabilising the charge on the amine.

| **Parallel amides** | **Anti-parallel amides** |
|---|---|
| 2° radical (**43a**) (0.0 kJ/mol)<br>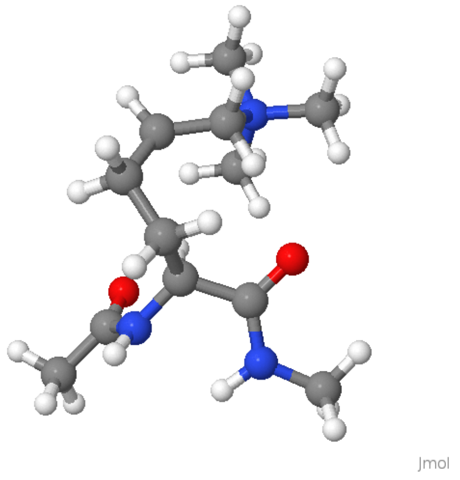<br> | 2° radical (**43a**) (7.8 kJ/mol)<br>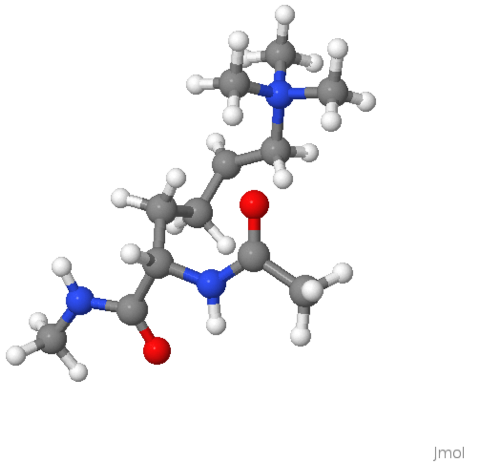<br> |

| | |
|---|---|
| Product **43b** (0.0 kJ/mol) 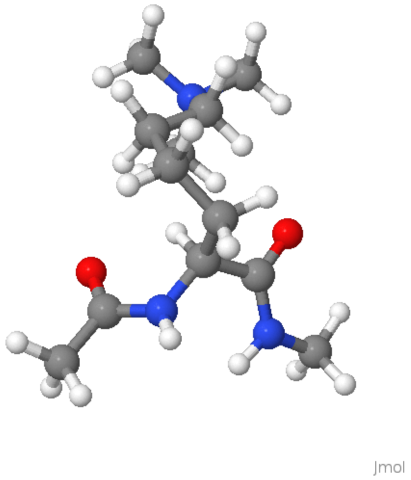 | Product **43b** (9.2 kJ/mol) 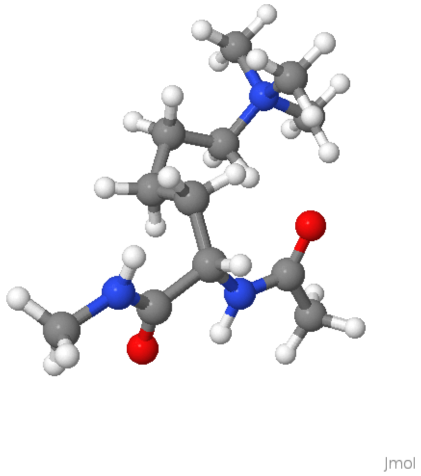 |
| 2° radical (**41a**) (3.2 kJ/mol) 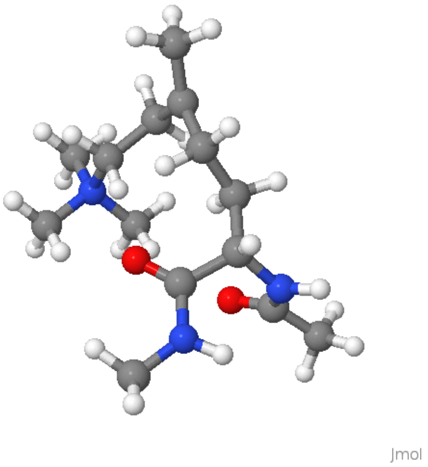 | 2° radical (**41a**) (0.0 kJ/mol) 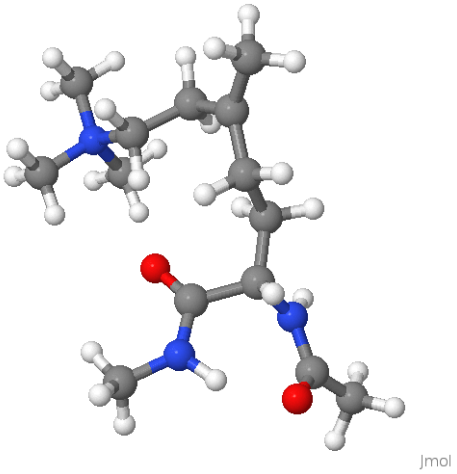 |
| Product **41b** (4.0 kJ/mol) 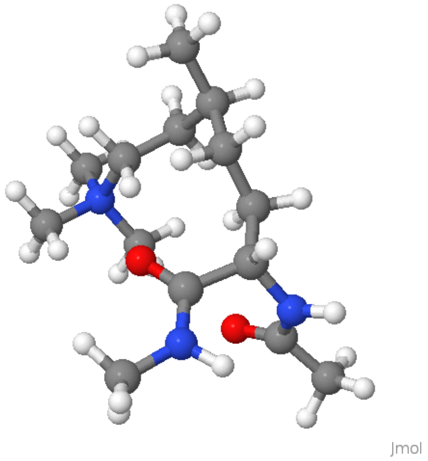 | Product **41b** (0.0 kJ/mol) 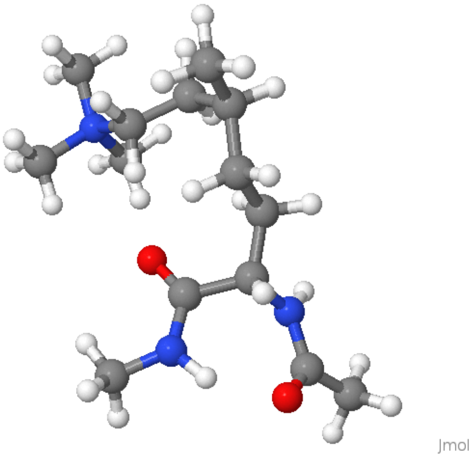 |

**Figure S79 -** PBE0/Def2-SVP optimised structures of the radical intermediates (**41a**, **43a**) and final products (**41b**, **43b**). Relative DFT total energies, E, between the parallel and anti-parallel

amide conformers of the radical intermediates and the products for each chain length given in brackets.

| Alanyl radical (**40**) | Amine **42** | Amine **11** |
|---|---|---|
| 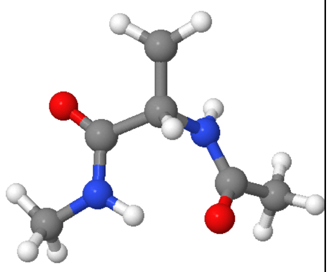 | 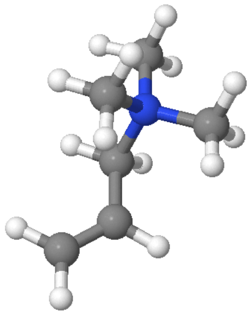 | 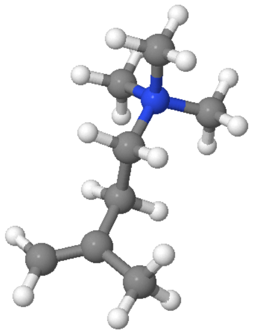 |

**Figure S80** - PBE0/Def2-SVP optimised structures of the alanyl radical **40** and the two amines (**11**, **42**).

| **Acetonitrile** | **Water** |
|---|---|
| 2° radical (**43a**) | 2° radical (**43a**) |
| 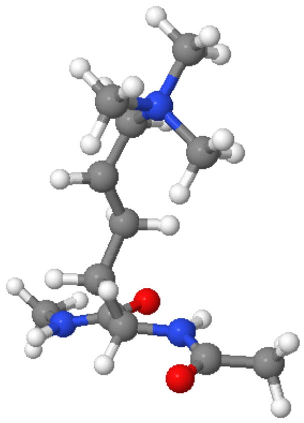 | 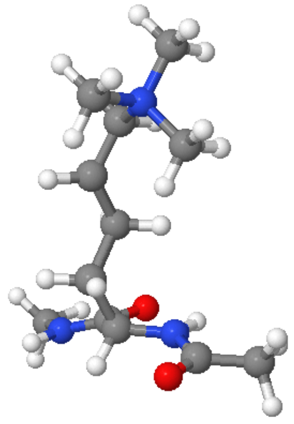 |
| Product **43a** | Product **43a** |

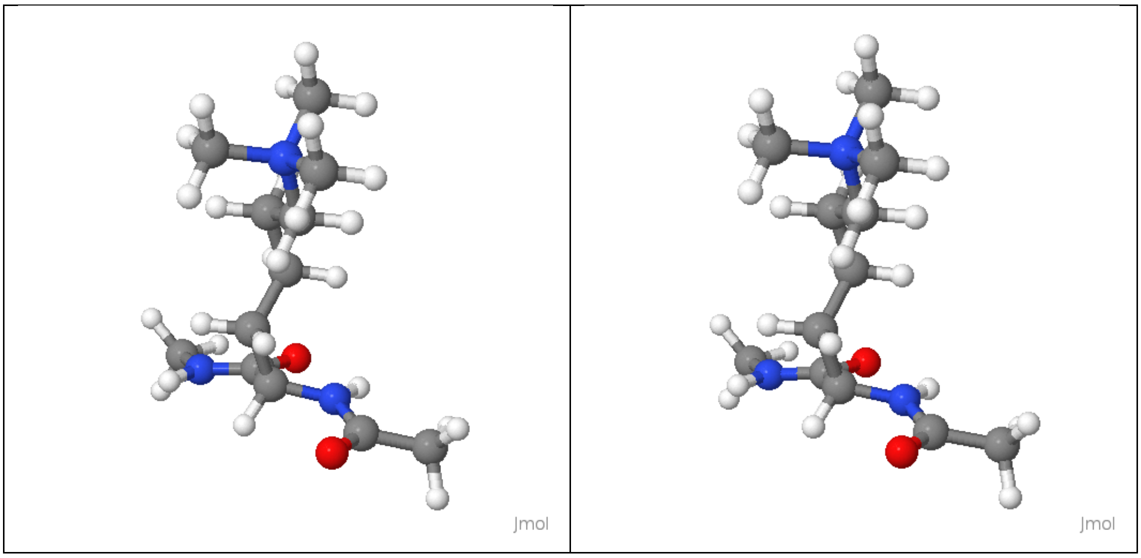


**Figure S81 -** PBE0/Def2-SVP optimised structures of the 2° radical (**43a**) and product **43b** with anti-parallel amide groups in bulk acetonitrile and water. Solvents described by the Polaraisable Continuum Model (PCM).

| **Step** | **DeltaE (kJ/mol)** | **DelE+ZPE (kJ/mol)** | **DelE+thermal (kJ/mol)** | **DelH (kJ/mol)** | **DelG (kJ/mol)** |
|---|---|---|---|---|---|
| **Parallel amides** | | | | | |
| Amine **42** + alanyl radical **40** to 2° radical **43a** | -179.8 | -162.5 | -162.7 | -165.2 | -109.1 |
| 2° radical **43a** + H to **43b** | -414.8 | -375.2 | -379.7 | -382.1 | -344.3 |
| Amine **42** + alanyl radical **40** + H to product **43b** | -594.6 | -537.7 | -542.3 | -547.3 | -453.5 |
| | | | | | |
| Amine **11** + alanyl | -191.6 | -173.8 | -173.7 | -176.2 | -115.4 |

| | | | | | |
|---|---|---|---|---|---|
| radical **40** to 3° radical **41a** | | | | | |
| 3° radical **41a** + H to product **41b** | -409.8 | -370.8 | -376.1 | -378.6 | -337.0 |
| Amine **11** + alanyl radical **40** + H to product **41b** | -601.4 | -544.6 | -549.8 | -554.8 | -452.4 |
| | | | | | |
| **Anti-parallel amides** | | | | | |
| Amine **42** + alanyl radical **40** to 2° radical **43a** | -171.9 | -154.7 | -154.7 | -157.2 | -100.4 |
| 2° radical **43a** + H to **43b** | -413.4 | -375.4 | -379.6 | -382.1 | -346.1 |
| Amine **42** + alanyl radical **40** + H to product **43b** | -585.3 | -530.1 | -534.3 | -539.2 | -446.6 |
| | | | | | |
| Amine **11** + alanyl radical **40** to 3° radical **41a** | -194.8 | -176.7 | -176.6 | -179.1 | -120.5 |

| | | | | | |
|---|---|---|---|---|---|
| 3° radical **41a** + H to product **41b** | -410.6 | -371.6 | -376.9 | -379.4 | -337.3 |
| Amine **11** + alanyl radical **40** + H to product **41b** | -605.4 | -548.4 | -553.5 | -558.5 | -457.8 |

**Table 7.** Energetics, at 298.15 K and 1 atm, for formation of radical intermediates (**41a**, **43a**) and final products (**41b**, **43b**) in gas phase at the PBE0/Def2-SVP level of theory.

| **Step** | **DeltaE (kJ/mol)** | **DelE+ZPE (kJ/mol)** | **DelE+thermal (kJ/mol)** | **DelH (kJ/mol)** | **DelG (kJ/mol)** |
|---|---|---|---|---|---|
| **Parallel amides** | | | | | |
| Amine **42** + alanyl radical **40** to 2° radical **43a** | -137.2 | -120.6 | -120.4 | -122.9 | -69.4 |
| 2° radical **43a** + H to **43b** | -421.3 | -381.8 | -386.3 | -388.8 | -349.8 |
| Amine **42** + alanyl radical **40** + H to product **43b** | -558.5 | -502.4 | -506.7 | -511.7 | -419.3 |
| | | | | | |
| Amine **11** + alanyl radical **40** to | -134.9 | -117.9 | -117.4 | -119.8 | -62.5 |

| | | | | | |
|---|---|---|---|---|---|
| 3° radical **41a** | | | | | |
| 3° radical **41a** + H to product **41b** | -409.6 | -371.3 | -376.2 | -378.7 | -338.5 |
| Amine **11** + alanyl radical **40** + H to product **41b** | -544.5 | -489.2 | -493.6 | -498.6 | -401.0 |
| | | | | | |
| **Anti-parallel amides** | | | | | |
| Amine **42** + alanyl radical **40** to 2° radical **43a** | -137.7 | -122.0 | -121.3 | -123.8 | -72.6 |
| 2° radical **43a** + H to **43b** | -417.0 | -378.7 | -382.5 | -385.0 | -350.6 |
| Amine **42** + alanyl radical **40** + H to product **43b** | -554.7 | -500.7 | -503.8 | -508.7 | -423.2 |
| | | | | | |
| Amine **11** + alanyl radical **40** to 3° radical **41a** | -145.6 | -127.8 | -127.5 | -130.0 | -72.7 |

| | | | | | |
|---|---|---|---|---|---|
| 3° radical **41a** + H to product **41b** | -409.6 | -371.3 | -376.4 | -378.9 | -337.3 |
| Amine **11** + alanyl radical **40** + H to product **41b** | -555.5 | -499.0 | -503.9 | -508.9 | -410.0 |

**Table 8.** Energetics, at 298.15 K and 1 atm, for formation of radical intermediates (**41a**, **43a**) and final products (**41b**, **43b**) in bulk solvent, acetonitrile, at the PBE0/Def2-SVP level of theory using the Polarisable Continuum Model (PCM). The dielectric constant of acetonitrile is 35.688.

| **Step** | **DeltaE (kJ/mol)** | **DelE+ZPE (kJ/mol)** | **DelE+thermal (kJ/mol)** | **DelH (kJ/mol)** | **DelG (kJ/mol)** |
|---|---|---|---|---|---|
| **Parallel amides** | | | | | |
| Amine **42** + alanyl radical **40** to 2° radical **43a** | -136.6 | -120.0 | -119.8 | -122.3 | -69.7 |
| 2° radical **43a** + H to **43b** | -558.2 | -502.1 | -506.4 | -511.4 | -418.8 |
| Amine **42** + alanyl radical **40** + H to product **43b** | -421.6 | -382.1 | -386.6 | -389.1 | -349.0 |
| | | | | | |
| Amine **11** + alanyl | -134.0 | -117.0 | -116.5 | -118.9 | -61.7 |

| | | | | | |
|---|---|---|---|---|---|
| radical **40** to 3° radical **41a** | | | | | |
| 3° radical **41a** + H to product **41b** | -543.7 | -488.4 | -492.8 | -497.7 | -400.7 |
| Amine **11** + alanyl radical **40** + H to product **41b** | -409.7 | -371.4 | -376.3 | -378.8 | -339.0 |
| | | | | | |
| **Anti-parallel amides** | | | | | |
| Amine **42** + alanyl radical **40** to 2° radical **43a** | -137.8 | -122.3 | -121.4 | -123.9 | -73.2 |
| 2° radical **43a** + H to **43b** | -544.8 | -500.7 | -503.9 | -508.8 | -422.7 |
| Amine **42** + alanyl radical **40** + H to product **43b** | -417.0 | -378.5 | -382.5 | -384.9 | -349.5 |
| | | | | | |
| Amine **11** + alanyl radical **40** | -144.8 | -127.0 | -126.7 | -129.2 | -71.5 |

| | | | | | |
|---|---|---|---|---|---|
| to 3° radical **41a** | | | | | |
| 3° radical **41a** + H to product **41b** | -554.7 | -498.2 | -503.1 | -508.0 | -409.1 |
| Amine **11** + alanyl radical **40** + H to product **41b** | -409.9 | -371.3 | -376.4 | -378.9 | -337.7 |

**Table 9.** Energetics, at 298.15 K and 1 atm, for formation of radical intermediates (**41a**, **43a**) and final products (**41b**, **43b**) in bulk solvent, water, at the PBE0/Def2-SVP level of theory using the Polarisable Continuum Model (PCM). The dielectric constant of water is 78.3553.

## References


[1] The Open Babel Package, version 3.1.1, http://openbabel.org

[2] N. M. O'Boyle, M. Banck, C. A. James, C. Morley, T. Vandermeersch, G. R. Hutchison, *J. Chem Inf.* **2011**, 3, 33.

[3] T. A. Halgren, *J. Comput. Chem.* **1996,** 17, 490-519.

[4] K. Throssell **2017**, Evaluating and improving approximate LCAO-MO theory with restored overlap and bond order bond energy corrections. MS dissertation. Middletown (Connecticut): Wesleyan University. doi:10.14418/wes01.3.76

[5] C. Adamo, V. Barone, *J. Chem. Phys.* **1999**, 110 (13), 6158-6170.

[6] F. Weigend, R. Ahlrichs, *Phys. Chem. Chem. Phys.* **2005**, 7 (18), 3297-3305.

[7] J. Tomasi, B. Mennucci, R. Cammi, *Chem. Rev.* **2005**, 105, 2999-3093.

[8] M. J. Frisch, G. W. Trucks, H. B. Schlegel, G. E. Scuseria, M. A. Robb, J. R. Cheeseman, G. Scalmani, V. Barone, G. A. Petersson, H. Nakatsuji, X. Li, M. Caricato, A. V. Marenich, J. Bloino, B. G. Janesko, R. Gomperts, B. Mennucci, H. P. Hratchian, J. V. Ortiz, A. F. Izmaylov, J. L. Sonnenberg, D. Williams-Young, F. Ding, F. Lipparini, F. Egidi, J. Goings, B. Peng, A. Petrone, T. Henderson, D. Ranasinghe, V. G. Zakrzewski, J. Gao, N. Rega, G. Zheng, W. Liang, M. Hada, M. Ehara, K. Toyota, R. Fukuda, J. Hasegawa, M. Ishida, T. Nakajima, Y. Honda, O. Kitao, H. Nakai, T. Vreven, K. Throssell, J. A. Montgomery, Jr., J. E. Peralta, F. Ogliaro, M. J. Bearpark, J. J. Heyd, E. N. Brothers, K. N. Kudin, V. N. Staroverov, T. A. Keith, R. Kobayashi, J. Normand, K. Raghavachari, A. P. Rendell, J. C. Burant, S. S. Iyengar, J. Tomasi, M. Cossi, J. M. Millam, M. Klene, C. Adamo, R. Cammi, J. W. Ochterski, R. L. Martin, K. Morokuma, O. Farkas, J. B. Foresman, D. J. Fox, Gaussian 16, Revision A.03, Gaussian, Inc., Wallingford CT, 2016.

[9] T. J. Barker, D. L. Boger, *J. Am. Chem. Soc.* **2012**, *134*, 13588-13591.

[10] T. P. Garner, J. Strachan, E. C. Shedden, J. E. Long, J. R. Cavey, B. Shaw, R. Layfield, M. S. Searle, *Biochemistry* **2011**, *50*, 9076-9087.

[11] M. D. Simon, F. Chu, L. R. Racki, C. Cecile, A. L. Burlingame, B. Panning, G. J. Narlikar, K. M. Shokat, *Cell* **2007**, *128*, 1003-1012.

Optimised geometries at the PBE0/Def2-SVP level of theory in vacuum, acetonitrile and water, where the bulk solvents are described by the Polarisable Continuum Model (PCM).

Tables 10 to 18 display atomic numbers and x, y and z Cartesian coordinates (Angstrom).

| **4C radical** (-785.3223968) | **4C product** (-785.980707) |
|---|---|
| 8 -0.091940 2.003036 0.422720 | 8 0.194510 2.186151 0.378558 |
| 6 1.009712 1.455994 0.358380 | 6 1.224321 1.520356 0.289768 |
| 6 1.091360 -0.052784 0.602175 | 6 1.137641 0.022853 0.586098 |
| 7 2.367440 -0.662373 0.330951 | 7 2.298244 -0.767520 0.266660 |
| 6 2.527408 -1.439609 -0.796869 | 6 2.225587 -1.694484 -0.747649 |
| 6 3.857424 -2.127906 -0.939022 | 6 3.438465 -2.561702 -0.940346 |
| 8 1.643706 -1.539222 -1.624962 | 8 1.235653 -1.789861 -1.452045 |
| 6 0.501342 -0.442658 1.966999 | 6 0.558682 -0.243130 1.982771 |
| 6 -0.405053 -1.669398 1.890455 | 6 -0.405081 -1.431687 2.043611 |
| 6 -1.627851 -1.526042 1.043536 | 6 -1.467465 -1.449933 0.935201 |
| 6 -2.321303 -0.228357 0.857827 | 6 -2.202679 -0.122474 0.824606 |
| 7 -2.912148 0.006431 -0.528763 | 7 -2.917550 0.121704 -0.489557 |
| 6 -3.981266 -0.981972 -0.826452 | 6 -3.840880 -0.995726 -0.821552 |
| 6 -1.847605 -0.070239 -1.572023 | 6 -1.923999 0.310163 -1.591002 |
| 6 -3.482766 1.384530 -0.536002 | 6 -3.696424 1.385710 -0.347181 |
| 7 2.147081 2.086144 0.049762 | 7 2.410840 2.005774 -0.094819 |
| 6 2.222322 3.493011 -0.260036 | 6 2.623505 3.383818 -0.465564 |
| 1 0.442408 -0.461508 -0.189270 | 1 0.409350 -0.305776 -0.168447 |
| 1 3.050605 -0.738685 1.075442 | 1 3.047391 -0.851848 0.944219 |
| 1 3.684347 -3.205471 -1.069978 | 1 3.134833 -3.615771 -0.867239 |
| 1 4.340682 -1.770412 -1.859950 | 1 3.817619 -2.410835 -1.961451 |
| 1 4.536908 -1.967823 -0.091115 | 1 4.246357 -2.364000 -0.223270 |
| 1 1.317430 -0.640908 2.680341 | 1 1.378812 -0.399717 2.702946 |
| 1 -0.049902 0.413830 2.383058 | 1 0.048280 0.672265 2.316942 |
| 1 0.167126 -2.541153 1.526271 | 1 0.150112 -2.381009 1.978840 |

| | |
|---|---|
| 1 -0.711509 -1.953765 2.918215<br>1 -2.162924 -2.450251 0.799872<br>1 -3.179394 -0.116540 1.548142<br>1 -1.638211 0.621654 1.014001<br>1 -4.436579 -0.735418 -1.793839<br>1 -3.546558 -1.986620 -0.875857<br>1 -4.742659 -0.940535 -0.037211<br>1 -2.308692 0.084852 -2.555475<br>1 -1.115574 0.719452 -1.365558<br>1 -1.361800 -1.053295 -1.534155<br>1 -3.904751 1.592405 -1.527110<br>1 -4.270226 1.451715 0.224958<br>1 -2.675552 2.093020 -0.308626<br>1 2.977152 1.509823 -0.029097<br>1 2.532180 3.655845 -1.303973<br>1 1.225817 3.926911 -0.111742<br>1 2.936587 4.003536 0.403127 | 1 -0.890011 -1.437735 3.033588<br>1 -0.966914 -1.701112 -0.014335<br>1 -2.173620 -2.270362 1.136390<br>1 -2.967830 -0.029156 1.609930<br>1 -1.511661 0.730490 0.915084<br>1 -4.400583 -0.738543 -1.729612<br>1 -3.261124 -1.909011 -0.995476<br>1 -4.537342 -1.147936 0.012897<br>1 -2.468064 0.524510 -2.519336<br>1 -1.273521 1.153240 -1.321574<br>1 -1.321731 -0.597834 -1.719002<br>1 -4.183976 1.617836 -1.302159<br>1 -4.453630 1.256504 0.435861<br>1 -3.006448 2.193404 -0.072084<br>1 3.166552 1.339097 -0.198251<br>1 2.895087 3.475105 -1.528788<br>1 1.688442 3.929471 -0.288401<br>1 3.421545 3.836113 0.142077 |
| **5C radical** (-863.8065974) | **5C product** (-863.8065974) |
| 8 0.449401 -0.585274 1.456162<br>6 1.545206 -0.082746 1.229354<br>6 1.705292 1.231883 0.431925<br>7 2.799057 1.094312 -0.514720<br>6 2.769997 0.113300 -1.466454<br>6 3.859314 0.157424 -2.504391<br>8 1.908209 -0.750030 -1.455140<br>6 0.406881 1.683864 -0.238933<br>6 -0.673442 2.214267 0.709043<br>6 -2.067052 2.194103 0.149450<br>6 -3.056377 3.201961 0.620624<br>6 -2.535813 0.968280 -0.589390<br>6 -2.384028 -0.266829 0.296483<br>7 -2.384205 -1.618231 -0.393706 | 8 0.413326 -0.575297 1.419947<br>6 1.522268 -0.085959 1.230183<br>6 1.729840 1.220568 0.429606<br>7 2.857146 1.059247 -0.473176<br>6 2.849485 0.068791 -1.416010<br>6 3.976262 0.088215 -2.414040<br>8 1.976556 -0.783043 -1.427037<br>6 0.465799 1.688318 -0.294809<br>6 -0.648157 2.212238 0.607735<br>6 -2.030375 2.303042 -0.059373<br>6 -3.071414 2.811236 0.934550<br>6 -2.469264 0.986656 -0.738347<br>6 -2.401286 -0.195290 0.213113<br>7 -2.393235 -1.573244 -0.420054 |

| | |
|---|---|
| 6 -1.257457 -1.723362 -1.369314 | 6 -1.225631 -1.741907 -1.337840 |
| 6 -2.170599 -2.644299 0.668297 | 6 -2.246856 -2.557408 0.692135 |
| 6 -3.675584 -1.865644 -1.083547 | 6 -3.656361 -1.830600 -1.157574 |
| 7 2.694663 -0.582938 1.708733 | 7 2.647064 -0.595057 1.756503 |
| 6 2.771849 -1.798860 2.478837 | 6 2.681611 -1.806783 2.536137 |
| 1 2.018937 1.994385 1.166093 | 1 2.026226 1.983722 1.170231 |
| 1 3.436107 1.871740 -0.634576 | 1 3.507797 1.827616 -0.577320 |
| 1 4.658809 0.875854 -2.279621 | 1 4.775320 0.800459 -2.168878 |
| 1 4.288452 -0.848596 -2.606315 | 1 4.397281 -0.923654 -2.488328 |
| 1 3.411023 0.415602 -3.475678 | 1 3.566507 0.339176 -3.404038 |
| 1 0.663937 2.468485 -0.968170 | 1 0.765527 2.477250 -1.004093 |
| 1 0.041178 0.833386 -0.827945 | 1 0.123707 0.844612 -0.908170 |
| 1 -0.644333 1.626444 1.649540 | 1 -0.720546 1.584683 1.510210 |
| 1 -0.426113 3.245559 1.011583 | 1 -0.382047 3.218518 0.972918 |
| 1 -2.606354 4.204809 0.695718 | 1 -1.963637 3.043069 -0.876643 |
| 1 -3.929172 3.270986 -0.046918 | 1 -2.783231 3.795115 1.333833 |
| 1 -3.446771 2.966351 1.632823 | 1 -4.060452 2.923403 0.464440 |
| 1 -3.579662 1.104819 -0.910975 | 1 -3.180077 2.135361 1.798737 |
| 1 -1.937428 0.846325 -1.510324 | 1 -3.487134 1.122101 -1.138619 |
| 1 -1.421914 -0.239607 0.827878 | 1 -1.815989 0.813522 -1.606241 |
| 1 -3.192879 -0.310950 1.040708 | 1 -1.469516 -0.167478 0.795160 |
| 1 -0.310494 -1.467711 -0.874508 | 1 -3.251238 -0.198052 0.910848 |
| 1 -1.220145 -2.753568 -1.745133 | 1 -0.299745 -1.469981 -0.813081 |
| 1 -1.434155 -1.042319 -2.208484 | 1 -1.185746 -2.791237 -1.655680 |
| 1 -1.212284 -2.431324 1.159816 | 1 -1.355880 -1.106052 -2.219681 |
| 1 -2.988896 -2.581540 1.396247 | 1 -1.300984 -2.348321 1.208656 |
| 1 -2.156383 -3.640339 0.208222 | 1 -3.089201 -2.441753 1.385353 |
| 1 -3.820833 -1.110979 -1.865301 | 1 -2.243703 -3.572369 0.275508 |
| 1 -3.658016 -2.864889 -1.536996 | 1 -3.755186 -1.105845 -1.973957 |
| 1 -4.492240 -1.804194 -0.353137 | 1 -3.633142 -2.847305 -1.569959 |
| 1 3.553527 -0.150116 1.391692 | 1 -4.503859 -1.729359 -0.467843 |
| 1 1.779238 -2.009838 2.894963 | 1 3.522090 -0.175471 1.467041 |
| 1 3.083984 -2.656534 1.860572 | 1 1.675128 -1.997129 2.928207 |

| | |
|---|---|
| 1 3.486273 -1.681861 3.306000 | 1 2.993627 -2.674521 1.931938<br>1 3.376691 -1.696681 3.380461 |

**Table 10.** PBE0/Def2-SVP optimised coordinates (Å) of the radical intermediates and final products with parallel amide groups in vacuum. Total energy (Hartree) in brackets.

| **4C radical** (-785.3194095) | **4C product** (-785.9771931) |
|---|---|
| 8 3.537975 0.542214 -0.908802 | 8 -3.484044 0.516307 1.003467 |
| 6 3.108994 -0.121618 0.019324 | 6 -3.101973 -0.089240 0.016617 |
| 6 1.757870 0.238233 0.670232 | 6 -1.760865 0.278995 -0.647659 |
| 7 1.362562 1.479764 0.051718 | 7 -1.320024 1.474014 0.031055 |
| 6 0.198560 2.140367 0.193587 | 6 -0.190427 2.170490 -0.192748 |
| 6 0.127171 3.480797 -0.492142 | 6 -0.081302 3.484553 0.537493 |
| 8 -0.762914 1.699797 0.828440 | 8 0.714939 1.776214 -0.932814 |
| 6 0.715318 -0.884483 0.561497 | 6 -0.746951 -0.873163 -0.626056 |
| 6 0.248336 -1.196849 -0.876035 | 6 -0.304375 -1.355970 0.759044 |
| 6 -1.199970 -1.548818 -0.967059 | 6 1.175881 -1.757439 0.768673 |
| 6 -2.213144 -0.464190 -0.969157 | 6 2.040539 -0.499190 0.769961 |
| 7 -3.347947 -0.571702 0.050523 | 7 3.330435 -0.534445 -0.021372 |
| 6 -2.799844 -0.717294 1.429627 | 6 3.035293 -0.673920 -1.478167 |
| 6 -4.229378 -1.723336 -0.270866 | 6 4.217493 -1.633976 0.438585 |
| 6 -4.123529 0.699949 -0.015307 | 6 4.004659 0.782602 0.180124 |
| 7 3.742853 -1.188564 0.541824 | 7 -3.779658 -1.100586 -0.558905 |
| 6 5.030165 -1.641999 0.067493 | 6 -5.062573 -1.556610 -0.075710 |
| 1 1.940604 0.401884 1.749753 | 1 -1.962901 0.513477 -1.710187 |
| 1 2.091705 1.845625 -0.563711 | 1 -2.011412 1.805650 0.706334 |
| 1 1.019584 3.725330 -1.082391 | 1 -0.918133 3.684190 1.219085 |
| 1 -0.759415 3.512515 -1.141179 | 1 0.862775 3.513411 1.099712 |
| 1 -0.012657 4.253160 0.278797 | 1 -0.032115 4.289013 -0.211496 |
| 1 1.113675 -1.795866 1.034235 | 1 -1.157164 -1.717046 -1.204200 |
| 1 -0.134437 -0.564916 1.173536 | 1 0.118069 -0.511050 -1.198859 |
| 1 0.439109 -0.301724 -1.497756 | 1 -0.472160 -0.564155 1.509361 |
| 1 0.860472 -2.001604 -1.308130 | 1 -0.919391 -2.207421 1.086643 |

| | |
|---|---|
| 1 -1.501456 -2.572936 -1.209276 | 1 1.405723 -2.381690 1.645452 |
| 1 -1.736055 0.498737 -0.742875 | 1 1.372404 -2.387856 -0.114548 |
| 1 -2.726257 -0.389130 -1.945035 | 1 1.493681 0.343926 0.325837 |
| 1 -3.636578 -0.724448 2.139513 | 1 2.325811 -0.224526 1.795866 |
| 1 -2.248365 -1.662946 1.492485 | 1 3.978355 -0.615219 -2.035753 |
| 1 -2.129267 0.131676 1.621410 | 1 2.560761 -1.642986 -1.666611 |
| 1 -5.043395 -1.769821 0.463553 | 1 2.362217 0.144263 -1.769379 |
| 1 -4.645732 -1.591332 -1.277578 | 1 5.155345 -1.603998 -0.130498 |
| 1 -3.644449 -2.649590 -0.228023 | 1 4.427958 -1.501763 1.507468 |
| 1 -4.967805 0.642948 0.683009 | 1 3.719284 -2.596957 0.276811 |
| 1 -3.453543 1.523485 0.264946 | 1 4.931946 0.805606 -0.405699 |
| 1 -4.495968 0.842602 -1.037493 | 1 3.319581 1.571348 -0.158789 |
| 1 3.344982 -1.637731 1.356481 | 1 4.233982 0.909436 1.245288 |
| 1 4.991542 -2.699884 -0.233042 | 1 -3.417687 -1.500568 -1.414880 |
| 1 5.301587 -1.032549 -0.803101 | 1 -5.041583 -2.633666 0.149048 |
| 1 5.806060 -1.520623 0.839151 | 1 -5.291412 -1.003874 0.843627 |
| | 1 -5.858754 -1.363394 -0.811236 |
| **5C radical** (-863.8078345) | **5C product** (-864.4645388) |
| 8 -0.170109 -1.246486 -0.195631 | 8 -0.176620 -1.223025 -0.174904 |
| 6 -1.285642 -0.806725 -0.496984 | 6 -1.292253 -0.790586 -0.485842 |
| 6 -1.736774 0.622441 -0.110613 | 6 -1.763239 0.629417 -0.089667 |
| 7 -2.768427 0.580707 0.909643 | 7 -2.815048 0.566032 0.908987 |
| 6 -4.076399 0.383169 0.592973 | 6 -4.114932 0.362214 0.564805 |
| 6 -5.070987 0.524768 1.711500 | 6 -5.131872 0.481850 1.665736 |
| 8 -4.416919 0.112882 -0.550938 | 8 -4.431556 0.103674 -0.588685 |
| 6 -0.560470 1.479156 0.334035 | 6 -0.604025 1.491255 0.389746 |
| 6 0.331670 1.921837 -0.832778 | 6 0.316982 1.932533 -0.745869 |
| 6 1.728194 2.329199 -0.467236 | 6 1.704732 2.434958 -0.324693 |
| 6 2.375930 3.464568 -1.180120 | 6 2.478503 2.952295 -1.534968 |
| 6 2.563559 1.392911 0.365146 | 6 2.514264 1.396617 0.482443 |
| 6 2.657467 0.032042 -0.317847 | 6 2.643496 0.079035 -0.258180 |
| 7 3.228008 -1.112009 0.499226 | 7 3.218482 -1.083673 0.526458 |
| 6 2.410521 -1.338440 1.727922 | 6 2.397870 -1.359127 1.743391 |

| | |
|---|---|
| 6 3.134414 -2.338077 -0.346369 | 6 3.141725 -2.283192 -0.358180 |
| 6 4.645053 -0.858840 0.865625 | 6 4.631813 -0.831800 0.909198 |
| 7 -2.210029 -1.497634 -1.166144 | 7 -2.200420 -1.482552 -1.176094 |
| 6 -2.054673 -2.871200 -1.571907 | 6 -2.025343 -2.849483 -1.595780 |
| 1 -2.208692 1.062336 -1.006630 | 1 -2.220445 1.076983 -0.989472 |
| 1 -2.509504 0.765558 1.870195 | 1 -2.576196 0.739862 1.876779 |
| 1 -4.615793 0.717745 2.692017 | 1 -4.697086 0.665944 2.657171 |
| 1 -5.754402 1.350116 1.463502 | 1 -5.817138 1.304829 1.415007 |
| 1 -5.679400 -0.388970 1.762135 | 1 -5.733650 -0.437256 1.693289 |
| 1 -0.944464 2.369979 0.855109 | 1 -1.012813 2.379092 0.898514 |
| 1 0.018510 0.898261 1.069337 | 1 -0.048360 0.912400 1.144967 |
| 1 0.367662 1.100651 -1.580352 | 1 0.433700 1.109350 -1.471814 |
| 1 -0.158462 2.754104 -1.362493 | 1 -0.179148 2.738231 -1.311502 |
| 1 1.671417 4.296557 -1.337183 | 1 1.558619 3.287331 0.362205 |
| 1 3.247229 3.856156 -0.632597 | 1 1.936775 3.777800 -2.019786 |
| 1 2.737330 3.176387 -2.189450 | 1 3.471010 3.333429 -1.249434 |
| 1 3.560002 1.832831 0.525009 | 1 2.620880 2.172623 -2.301300 |
| 1 2.102017 1.277751 1.362618 | 1 3.501726 1.830558 0.709674 |
| 1 1.651877 -0.316302 -0.592752 | 1 2.009912 1.238201 1.447396 |
| 1 3.278836 0.093794 -1.223513 | 1 1.650605 -0.276690 -0.566184 |
| 1 1.358845 -1.430900 1.421858 | 1 3.282830 0.179172 -1.147213 |
| 1 2.757719 -2.259651 2.212585 | 1 1.347218 -1.442448 1.431155 |
| 1 2.542876 -0.496226 2.415659 | 1 2.746590 -2.297507 2.192499 |
| 1 2.075407 -2.510508 -0.578539 | 1 2.526781 -0.544213 2.463750 |
| 1 3.704306 -2.180668 -1.270396 | 1 2.085211 -2.462592 -0.596098 |
| 1 3.549898 -3.189730 0.206872 | 1 3.709074 -2.087971 -1.276548 |
| 1 4.705599 0.039526 1.490674 | 1 3.569253 -3.146263 0.167275 |
| 1 5.032886 -1.720692 1.423433 | 1 4.682584 0.048804 1.559650 |
| 1 5.233803 -0.714453 -0.049114 | 1 5.021330 -1.706867 1.444722 |
| 1 -3.117411 -1.030481 -1.285530 | 1 5.225315 -0.657819 0.002730 |
| 1 -1.078248 -3.234368 -1.229596 | 1 -3.109601 -1.021744 -1.306226 |
| 1 -2.843432 -3.500440 -1.132016 | 1 -1.060484 -3.214603 -1.223869 |
| 1 -2.108214 -2.967839 -2.667455 | 1 -2.827231 -3.486769 -1.193059 |

| | |
|---|---|
| | 1 -2.039286 -2.931640 -2.693861 |

**Table 11.** PBE0/Def2-SVP optimised coordinates (Å) of the radical intermediates and final products with anti-parallel amide groups in vacuum. Total energy (Hartree) in brackets.

| **Alanyl radical** (-494.2710903) | **H atom** (-0.5002902) |
|---|---|
| 8 2.308987 0.805007 0.611730<br>6 1.361647 0.282315 0.055461<br>6 0.134412 1.094728 -0.422110<br>7 -1.081298 0.713307 0.291954<br>6 -1.842305 -0.345396 -0.071927<br>6 -3.113131 -0.555223 0.712580<br>8 -1.520269 -1.093098 -0.986546<br>6 0.374309 2.553276 -0.319641<br>7 1.262658 -1.036332 -0.222119<br>6 2.273283 -1.983488 0.162522<br>1 -0.048948 0.798579 -1.474957<br>1 -1.336823 1.264684 1.101096<br>1 -3.295486 0.210094 1.479025<br>1 -3.956336 -0.570390 0.007367<br>1 -3.073897 -1.545476 1.188839<br>1 -0.402657 3.252741 -0.635010<br>1 1.346566 2.909468 0.022615<br>1 0.409425 -1.351217 -0.686889<br>1 3.087431 -1.431219 0.649758<br>1 1.881336 -2.733683 0.869294<br>1 2.680835 -2.514949 -0.712778 | |
| **Amine 4C** (-290.9828281) | **Amine 5C** (-369.4625198) |
| 6 2.758830 -0.284970 0.053266<br>6 1.702794 0.494911 -0.189416<br>6 0.467032 0.003172 -0.866352<br>7 -0.777467 -0.024004 0.014807<br>6 -1.064881 1.335406 0.555863 | 6 2.985358 -1.190905 0.254130<br>6 2.266718 -0.109788 -0.066780<br>6 2.785628 1.289888 0.083079<br>6 0.864993 -0.260653 -0.624876<br>6 -0.153829 0.017010 0.470198 |

| | |
|---|---|
| 6 -0.585143 -0.977281 1.144579 | 7 -1.614334 0.010388 0.055623 |
| 6 -1.929204 -0.472202 -0.819192 | 6 -1.989543 -1.302112 -0.544474 |
| 1 3.671733 0.121633 0.496030 | 6 -2.437864 0.235320 1.279906 |
| 1 2.764752 -1.350819 -0.198571 | 6 -1.895297 1.102395 -0.920036 |
| 1 1.761936 1.564282 0.041162 | 1 2.600554 -2.204752 0.109149 |
| 1 0.202869 0.641094 -1.724219 | 1 3.996195 -1.100480 0.660027 |
| 1 0.598240 -1.024822 -1.233210 | 1 3.787777 1.299132 0.531275 |
| 1 -2.008528 1.302471 1.114395 | 1 2.849687 1.790221 -0.897994 |
| 1 -0.252921 1.640691 1.225345 | 1 2.135568 1.916909 0.717135 |
| 1 -1.151896 2.044375 -0.277260 | 1 0.738174 0.440321 -1.467042 |
| 1 -1.482159 -0.966412 1.775957 | 1 0.741361 -1.280755 -1.017142 |
| 1 -0.427453 -1.985054 0.740419 | 1 -0.062650 -0.736446 1.266013 |
| 1 0.292340 -0.671257 1.726114 | 1 0.017105 1.003175 0.925753 |
| 1 -2.827960 -0.526909 -0.192483 | 1 -1.744575 -2.103800 0.163527 |
| 1 -2.087147 0.246609 -1.632726 | 1 -3.067522 -1.304000 -0.748392 |
| 1 -1.708105 -1.462072 -1.237098 | 1 -1.439310 -1.449765 -1.480080 |
| | 1 -2.248141 -0.574697 1.994689 |
| | 1 -2.159846 1.196966 1.728415 |
| | 1 -3.499526 0.247051 1.003652 |
| | 1 -1.328758 0.929026 -1.841259 |
| | 1 -2.968761 1.109067 -1.146393 |
| | 1 -1.603977 2.063179 -0.477571 |

**Table 12.** PBE0/Def2-SVP optimised coordinates (Å) of the alanyl radical and the two amines in vacuum. Total energy (Hartree) in brackets.

| **4C radical** (-785.3932708) | **4C product** (-786.0540725) |
|---|---|
| 8 -0.042628 1.986966 0.390886 | 8 2.058608 2.480303 0.201802 |
| 6 1.048422 1.417328 0.341994 | 6 2.297700 1.291904 0.031165 |
| 6 1.102552 -0.101202 0.537146 | 6 1.259037 0.233500 0.431154 |

| | |
|---|---|
| 7 2.404727 -0.693303 0.363836<br>6 2.755833 -1.346880 -0.781975<br>6 4.113619 -1.998981 -0.771804<br>8 2.018489 -1.392831 -1.753881<br>6 0.447495 -0.516358 1.864655<br>6 -0.454021 -1.741457 1.733127<br>6 -1.656211 -1.568548 0.864386<br>6 -2.410149 -0.291487 0.826218<br>7 -3.100458 0.013589 -0.490189<br>6 -4.156107 -0.991731 -0.787686<br>6 -2.101331 0.045294 -1.595634<br>6 -3.722499 1.362830 -0.366800<br>7 2.208711 2.035572 0.097995<br>6 2.323687 3.449365 -0.150264<br>1 0.498844 -0.486631 -0.302030<br>1 3.013311 -0.767213 1.171011<br>1 3.986641 -3.076515 -0.950822<br>1 4.702535 -1.597316 -1.608754<br>1 4.668559 -1.851238 0.163588<br>1 1.234662 -0.722793 2.607052<br>1 -0.123078 0.332486 2.268108<br>1 0.129002 -2.601090 1.360583<br>1 -0.783490 -2.044590 2.748482<br>1 -2.147263 -2.481376 0.512939<br>1 -3.213588 -0.266504 1.585823<br>1 -1.746843 0.568192 1.003151<br>1 -4.671637 -0.698762 -1.709695<br>1 -3.695632 -1.975683 -0.923853<br>1 -4.867477 -1.019829 0.046262<br>1 -2.617042 0.297444 -2.529550<br>1 -1.349229 0.806273 -1.353368<br>1 -1.632042 -0.941682 -1.681511<br>1 -4.232163 1.608254 -1.305539 | 7 1.761878 -1.119095 0.409859<br>6 1.264998 -2.076574 -0.421374<br>6 1.842191 -3.456007 -0.250387<br>8 0.399428 -1.836930 -1.254336<br>6 0.599438 0.615560 1.758571<br>6 -0.527556 -0.309036 2.212796<br>6 -1.622393 -0.602865 1.184821<br>6 -2.373287 0.641565 0.748174<br>7 -3.226955 0.465716 -0.488766<br>6 -4.120908 -0.719174 -0.357576<br>6 -2.365744 0.310194 -1.698451<br>6 -4.065245 1.687956 -0.652709<br>7 3.426217 0.823586 -0.530018<br>6 4.480812 1.675973 -1.012529<br>1 0.508741 0.267461 -0.376040<br>1 2.401110 -1.406707 1.143184<br>1 1.049430 -4.123392 0.119102<br>1 2.152230 -3.831998 -1.235176<br>1 2.693759 -3.493882 0.440942<br>1 1.369155 0.643883 2.548290<br>1 0.254815 1.654529 1.651127<br>1 -0.102857 -1.279451 2.515633<br>1 -0.977108 0.117301 3.125038<br>1 -1.169589 -1.104116 0.315596<br>1 -2.325518 -1.321952 1.631006<br>1 -3.049255 0.990259 1.542208<br>1 -1.690388 1.469517 0.512125<br>1 -4.806614 -0.735225 -1.212336<br>1 -3.518115 -1.632751 -0.353599<br>1 -4.689986 -0.636578 0.575824 |

| | |
|---|---|
| 1 -4.443781 1.349877 0.458680<br>1 -2.930109 2.093065 -0.164413<br>1 3.041044 1.457386 0.067726<br>1 2.725909 3.644751 -1.156249<br>1 1.323246 3.892503 -0.074176<br>1 2.984134 3.927851 0.588856 | 1 -3.011631 0.130869 -2.565764<br>1 -1.799684 1.237497 -1.846672<br>1 -1.674729 -0.531323 -1.565516<br>1 -4.633691 1.606511 -1.586430<br>1 -4.751287 1.767631 0.198183<br>1 -3.409954 2.565791 -0.689327<br>1 3.513606 -0.179598 -0.638421<br>1 4.611854 1.575861 -2.101605<br>1 4.210781 2.714217 -0.782788<br>1 5.438410 1.439240 -0.523782 |
| **5C radical** (-863.8719156) | **5C product** (-864.5282766) |
| 8 -0.443531 -0.827354 -1.291766<br>6 -1.562723 -0.337718 -1.166727<br>6 -1.785757 1.056260 -0.543863<br>7 -2.947506 1.042650 0.323637<br>6 -3.030063 0.203085 1.388873<br>6 -4.223997 0.391458 2.288476<br>8 -2.184589 -0.656632 1.595738<br>6 -0.527626 1.594429 0.139505<br>6 0.528679 2.123010 -0.834516<br>6 1.911337 2.262880 -0.270349<br>6 2.832262 3.274559 -0.859463<br>6 2.479057 1.136684 0.549685<br>6 2.492305 -0.152244 -0.269478<br>7 2.702548 -1.439676 0.497655<br>6 1.596609 -1.655467 1.475134<br>6 2.667801 -2.552075 -0.495033<br>6 4.012239 -1.439164 1.201495<br>7 -2.679693 -0.929240 -1.619230<br>6 -2.698907 -2.227771 -2.240224<br>1 -2.044372 1.720287 -1.386071<br>1 -3.616572 1.797597 0.240721 | 8 0.408448 -0.968707 1.106133<br>6 1.546471 -0.506538 1.092112<br>6 1.840850 0.935641 0.630144<br>7 3.069668 0.987177 -0.137840<br>6 3.227656 0.271689 -1.282625<br>6 4.490704 0.539549 -2.059725<br>8 2.392926 -0.542563 -1.651393<br>6 0.644313 1.567533 -0.084487<br>6 -0.450424 2.039560 0.867462<br>6 -1.790279 2.382388 0.203734<br>6 -2.778973 2.917988 1.236727<br>6 -2.382625 1.220018 -0.618190<br>6 -2.543544 -0.044353 0.206259<br>7 -2.850587 -1.306049 -0.569978<br>6 -1.719258 -1.649081 -1.479890<br>6 -3.001636 -2.407383 0.423838<br>6 -4.107884 -1.172168 -1.352628<br>7 2.619406 -1.177599 1.540088 |

| | |
|---|---|
| 1 -4.985243 1.064402 1.873002 | 6 2.568614 -2.532345 2.023810 |
| 1 -4.672263 -0.591048 2.489608 | 1 2.040171 1.505880 1.553473 |
| 1 -3.877744 0.797355 3.251030 | 1 3.739313 1.713789 0.081390 |
| 1 -0.834107 2.406117 0.817477 | 1 5.224567 1.143271 -1.510372 |
| 1 -0.126721 0.794345 0.775369 | 1 4.942353 -0.421151 -2.342018 |
| 1 0.560853 1.451281 -1.717830 | 1 4.225183 1.063614 -2.990370 |
| 1 0.201207 3.098427 -1.232794 | 1 1.016451 2.424851 -0.667936 |
| 1 2.307639 4.216854 -1.085391 | 1 0.263381 0.836785 -0.810842 |
| 1 3.677567 3.500346 -0.190276 | 1 -0.613514 1.278417 1.647880 |
| 1 3.272982 2.926904 -1.817706 | 1 -0.095223 2.936346 1.403727 |
| 1 3.492268 1.397746 0.889755 | 1 -1.604527 3.190529 -0.526107 |
| 1 1.865681 0.998516 1.456937 | 1 -2.378212 3.816856 1.729810 |
| 1 1.526794 -0.290760 -0.776038 | 1 -3.739097 3.190058 0.771545 |
| 1 3.287090 -0.118124 -1.028139 | 1 -2.985113 2.179518 2.028606 |
| 1 0.638403 -1.568531 0.946912 | 1 -3.350238 1.545923 -1.031069 |
| 1 1.704170 -2.656860 1.907894 | 1 -1.724014 1.032186 -1.479038 |
| 1 1.659786 -0.908863 2.273234 | 1 -1.612238 -0.274946 0.741761 |
| 1 1.691743 -2.536430 -0.994633 | 1 -3.356631 0.058557 0.938114 |
| 1 3.468940 -2.401603 -1.227504 | 1 -0.795114 -1.675083 -0.888530 |
| 1 2.813091 -3.503729 0.029354 | 1 -1.918176 -2.627911 -1.931556 |
| 1 4.024093 -0.636113 1.946467 | 1 -1.643230 -0.895215 -2.270060 |
| 1 4.149686 -2.406267 1.699635 | 1 -2.065212 -2.495929 0.987199 |
| 1 4.810892 -1.280724 0.467017 | 1 -3.828159 -2.164719 1.101694 |
| 1 -3.561409 -0.473733 -1.414066 | 1 -3.214977 -3.342472 -0.106680 |
| 1 -1.667645 -2.509932 -2.484863 | 1 -3.984635 -0.389909 -2.108963 |
| 1 -3.123711 -2.993402 -1.570718 | 1 -4.321570 -2.128451 -1.844574 |
| 1 -3.292883 -2.205874 -3.165694 | |

| | |
|---|---|
| | 1 -4.926150 -0.910608 -0.671252<br>1 3.522967 -0.731964 1.429993<br>1 1.517501 -2.806597 2.174854<br>1 3.016682 -3.238041 1.305421<br>1 3.103139 -2.623129 2.980846 |

**Table 13.** PBE0/Def2-SVP optimised coordinates (Å) of the radical intermediates and final products with parallel amide groups in acetonitrile. Total energy (Hartree) in brackets.

| **4C radical** (-785.3934569) | **4C product** (-786.0526193) |
|---|---|
| 8 2.871169 -0.821874 -1.491544<br>6 2.809952 -0.886711 -0.268711<br>6 2.056502 0.177119 0.533470<br>7 1.923180 1.330320 -0.316565<br>6 1.708453 2.574015 0.166222<br>6 1.649992 3.674945 -0.863844<br>8 1.565900 2.795304 1.362985<br>6 0.702061 -0.351501 1.036379<br>6 -0.252108 -0.767382 -0.076567<br>6 -1.573222 -1.231655 0.420969<br>6 -2.767372 -1.292374 -0.453674<br>7 -3.858206 -0.272905 -0.117357<br>6 -3.315988 1.109047 -0.221858<br>6 -4.366189 -0.505958 1.262422<br>6 -4.973609 -0.442219 -1.088332<br>7 3.343092 -1.882950 0.458888<br>6 4.036115 -3.005531 -0.121634<br>1 2.650624 0.458217 1.419287<br>1 2.100537 1.161772 -1.303078<br>1 1.816071 3.325091 -1.890994<br>1 0.666291 4.163046 -0.803485<br>1 2.404932 4.433868 -0.612588<br>1 0.876265 -1.198887 1.719672 | 8 3.257806 -0.841949 -1.279618<br>6 2.869591 -0.958955 -0.122822<br>6 2.014232 0.129347 0.531165<br>7 2.154359 1.314621 -0.272107<br>6 1.890753 2.551912 0.201053<br>6 2.149981 3.689421 -0.755625<br>8 1.465444 2.741550 1.335028<br>6 0.548497 -0.315204 0.684787<br>6 -0.125247 -0.684903 -0.630679<br>6 -1.594401 -1.091613 -0.498933<br>6 -2.468468 0.092228 -0.130092<br>7 -3.946633 -0.190867 0.012917<br>6 -4.197119 -1.180559 1.098821<br>6 -4.521940 -0.696413 -1.265664<br>6 -4.620823 1.091703 0.367918<br>7 3.114599 -2.033009 0.647221<br>6 3.854203 -3.183535 0.192740<br>1 2.404758 0.339130 1.541698<br>1 2.565495 1.167050 -1.190015<br>1 2.525576 3.363664 -1.734216<br>1 1.215985 4.252622 -0.896036<br>1 2.877559 4.375905 -0.298743<br>1 0.502520 -1.167985 1.382796 |

| | |
|---|---|
| 1 0.249135 0.450261 1.640306 | 1 0.030069 0.520439 1.180524 |
| 1 -0.382793 0.066187 -0.789575 | 1 -0.036478 0.155365 -1.340045 |
| 1 0.208522 -1.579020 -0.679720 | 1 0.411116 -1.528659 -1.093890 |
| 1 -1.629024 -1.698325 1.409775 | 1 -1.919869 -1.517033 -1.460002 |
| 1 -2.507725 -1.106767 -1.505560 | 1 -1.678661 -1.890944 0.254463 |
| 1 -3.269165 -2.270395 -0.391748 | 1 -2.162715 0.529015 0.830263 |
| 1 -4.114640 1.820512 0.017602 | 1 -2.388258 0.879682 -0.893478 |
| 1 -2.489346 1.222517 0.489094 | 1 -5.279712 -1.289334 1.230248 |
| 1 -2.960329 1.277522 -1.244998 | 1 -3.762418 -2.146477 0.823019 |
| 1 -5.178176 0.202305 1.463180 | 1 -3.743481 -0.813294 2.026852 |
| 1 -4.739031 -1.534296 1.335594 | 1 -5.605426 -0.804977 -1.142514 |
| 1 -3.551002 -0.349416 1.977243 | 1 -4.306775 0.025027 -2.062577 |
| 1 -5.757280 0.289383 -0.859625 | 1 -4.082083 -1.669002 -1.508002 |
| 1 -4.592600 -0.276781 -2.102755 | 1 -5.696205 0.909872 0.476229 |
| 1 -5.373418 -1.458987 -0.999579 | 1 -4.205441 1.462437 1.312015 |
| 1 3.282446 -1.827722 1.468451 | 1 -4.442317 1.820000 -0.431325 |
| 1 3.558750 -3.956011 0.161746 | 1 2.789574 -2.020675 1.606356 |
| 1 4.001428 -2.900077 -1.212585 | 1 3.251379 -4.100774 0.275534 |
| 1 5.089007 -3.033468 0.198989 | 1 4.119039 -3.024631 -0.859443 |
| | 1 4.778933 -3.319022 0.774791 |
| **5C radical** (-863.8759886) | **5C product** (-864.5324541) |
| 8 -0.263623 -1.281231 -0.335315 | 8 -0.271252 -1.277297 -0.298250 |
| 6 -1.364801 -0.791727 -0.584341 | 6 -1.370656 -0.792686 -0.563654 |
| 6 -1.765094 0.625018 -0.114083 | 6 -1.783540 0.623937 -0.104292 |
| 7 -2.751940 0.540508 0.947505 | 7 -2.789498 0.539254 0.939724 |
| 6 -4.072560 0.403200 0.700123 | 6 -4.105003 0.396752 0.669881 |
| 6 -4.985268 0.432580 1.895720 | 6 -5.038952 0.427543 1.848994 |
| 8 -4.506847 0.266346 -0.443001 | 8 -4.519098 0.254283 -0.480141 |
| 6 -0.555084 1.429966 0.331707 | 6 -0.585655 1.436805 0.360096 |
| 6 0.318412 1.889241 -0.838531 | 6 0.318935 1.869736 -0.789140 |
| 6 1.718211 2.292316 -0.483878 | 6 1.699998 2.393144 -0.374942 |
| 6 2.405985 3.322780 -1.310843 | 6 2.470274 2.894512 -1.594280 |
| 6 2.542514 1.379230 0.381921 | 6 2.517902 1.382886 0.454841 |

| | |
|---|---|
| 6 2.693897 0.019043 -0.294285 | 6 2.679253 0.051228 -0.254602 |
| 7 3.307320 -1.086900 0.535799 | 7 3.293607 -1.065186 0.559729 |
| 6 2.452588 -1.386123 1.721102 | 6 2.437788 -1.389562 1.737981 |
| 6 3.356699 -2.304789 -0.323528 | 6 3.350901 -2.268734 -0.319111 |
| 6 4.683031 -0.733362 0.975744 | 6 4.667364 -0.716373 1.010158 |
| 7 -2.332338 -1.422636 -1.264325 | 7 -2.326075 -1.429257 -1.255836 |
| 6 -2.224598 -2.780079 -1.732450 | 6 -2.206308 -2.787068 -1.719683 |
| 1 -2.263815 1.122640 -0.962875 | 1 -2.268491 1.116887 -0.963704 |
| 1 -2.429592 0.596890 1.906313 | 1 -2.484133 0.601128 1.903774 |
| 1 -4.456707 0.546095 2.850825 | 1 -4.527761 0.542565 2.813309 |
| 1 -5.692533 1.265806 1.773626 | 1 -5.744201 1.260369 1.713154 |
| 1 -5.574906 -0.495090 1.912787 | 1 -5.628476 -0.500306 1.856732 |
| 1 -0.902406 2.307705 0.898277 | 1 -0.952569 2.324892 0.898961 |
| 1 0.022635 0.804070 1.028701 | 1 -0.030088 0.825216 1.087953 |
| 1 0.354209 1.076360 -1.595753 | 1 0.445343 1.034362 -1.498891 |
| 1 -0.180430 2.726531 -1.353704 | 1 -0.190735 2.661295 -1.363654 |
| 1 1.717699 4.129904 -1.607777 | 1 1.541005 3.258027 0.293329 |
| 1 3.259625 3.772515 -0.779208 | 1 1.915827 3.699527 -2.100267 |
| 1 2.811810 2.896357 -2.252419 | 1 3.456267 3.293282 -1.310014 |
| 1 3.525175 1.837653 0.568071 | 1 2.630786 2.096211 -2.337098 |
| 1 2.054585 1.257964 1.364364 | 1 3.498410 1.833649 0.674608 |
| 1 1.707744 -0.370293 -0.583416 | 1 2.016405 1.239020 1.423393 |
| 1 3.319542 0.104977 -1.193788 | 1 1.699824 -0.342357 -0.559138 |
| 1 1.427349 -1.560263 1.369978 | 1 3.313292 0.149493 -1.146573 |
| 1 2.848715 -2.278161 2.220448 | 1 1.413350 -1.558523 1.381949 |
| 1 2.480676 -0.540332 2.415735 | 1 2.835579 -2.290439 2.219694 |
| 1 2.333553 -2.553392 -0.629511 | 1 2.463714 -0.557733 2.449203 |
| 1 3.973618 -2.095399 -1.204897 | 1 2.329641 -2.517228 -0.631273 |
| 1 3.791555 -3.130712 0.251011 | 1 3.968597 -2.041950 -1.195647 |
| 1 4.641096 0.140052 1.635216 | 1 3.788720 -3.101721 0.242739 |
| 1 5.110177 -1.583604 1.520325 | 1 4.621390 0.142478 1.688029 |
| 1 5.293386 -0.509165 0.092803 | 1 5.095803 -1.577014 1.537015 |
| 1 -3.227752 -0.932445 -1.332745 | 1 5.278779 -0.471614 0.133420 |

| | |
|---|---|
| 1 -1.204473 -3.135752 -1.543471<br>1 -2.932739 -3.442299 -1.209214<br>1 -2.428929 -2.840099 -2.812297 | 1 -3.221727 -0.941902 -1.340047<br>1 -1.190654 -3.141729 -1.506255<br>1 -2.926123 -3.449024 -1.212344<br>1 -2.385011 -2.849133 -2.804032 |

**Table 14.** PBE0/Def2-SVP optimised coordinates (Å) of the radical intermediates and final products with anti-parallel amide groups in acetonitrile. Total energy (Hartree) in brackets.

| **Alanyl radical** (-494.2823959) | **H atom** (-0.5003064) |
|---|---|
| 8 -2.338831 0.811698 -0.575475<br>6 -1.367772 0.284540 -0.052031<br>6 -0.133011 1.094781 0.406827<br>7 1.075067 0.699752 -0.308669<br>6 1.842732 -0.342823 0.071875<br>6 3.115615 -0.556678 -0.701169<br>8 1.516373 -1.083099 0.999541<br>6 -0.357176 2.555631 0.307165<br>7 -1.257816 -1.032541 0.202140<br>6 -2.262878 -1.993899 -0.167044<br>1 0.048067 0.802208 1.461662<br>1 1.345773 1.254562 -1.112077<br>1 3.297972 0.205924 -1.468814<br>1 3.955774 -0.566001 0.007912<br>1 3.078416 -1.548285 -1.175144<br>1 0.454877 3.242028 0.554442<br>1 -1.345935 2.933305 0.047328<br>1 -0.391721 -1.344999 0.646293<br>1 -3.113641 -1.455190 -0.602731<br>1 -1.881880 -2.713126 -0.910029<br>1 -2.613864 -2.559012 0.710598 | |
| **Amine 4C** (-291.0585962) | **Amine 5C** (-369.5381362) |
| 6 2.759154 -0.280638 0.053560<br>6 1.698620 0.489996 -0.195487 | 6 -3.013654 1.173582 0.251091<br>6 -2.265466 0.109605 -0.064478 |

| | |
|---|---|
| 6 0.463671 -0.018849 -0.862381 | 6 -2.766370 -1.298458 0.068770 |
| 7 -0.775149 -0.023458 0.015812 | 6 -0.859073 0.286198 -0.594558 |
| 6 -1.078946 1.351709 0.502543 | 6 0.161942 -0.053132 0.481826 |
| 6 -0.576822 -0.930970 1.180066 | 7 1.611568 -0.009262 0.053803 |
| 6 -1.918343 -0.515348 -0.804055 | 6 1.972976 1.337247 -0.473745 |
| 1 3.667633 0.135472 0.496458 | 6 2.447824 -0.298085 1.255518 |
| 1 2.763390 -1.349093 -0.185411 | 6 1.891856 -1.041226 -0.984276 |
| 1 1.742700 1.559375 0.036456 | 1 -2.635820 2.193521 0.132879 |
| 1 0.202769 0.600676 -1.733273 | 1 -4.032019 1.057835 0.633113 |
| 1 0.596761 -1.054152 -1.204036 | 1 -3.792598 -1.324843 0.459677 |
| 1 -2.023630 1.326591 1.057900 | 1 -2.754182 -1.808982 -0.908883 |
| 1 -0.273925 1.689103 1.163637 | 1 -2.133435 -1.899740 0.742361 |
| 1 -1.168354 2.023980 -0.359011 | 1 -0.727684 -0.367248 -1.472493 |
| 1 -1.480287 -0.909926 1.799933 | 1 -0.729744 1.325290 -0.928348 |
| 1 -0.397858 -1.947979 0.812007 | 1 0.072695 0.650472 1.321160 |
| 1 0.287436 -0.586327 1.758484 | 1 0.002748 -1.066126 0.876632 |
| 1 -2.815861 -0.552985 -0.176381 | 1 1.721296 2.095204 0.277308 |
| 1 -2.076609 0.171567 -1.643299 | 1 3.049608 1.356034 -0.677273 |
| 1 -1.682127 -1.517495 -1.179621 | 1 1.422977 1.526674 -1.401037 |
| | 1 2.263820 0.474008 2.011230 |
| | 1 2.173571 -1.282656 1.651227 |
| | 1 3.504254 -0.293665 0.962956 |
| | 1 1.321644 -0.815236 -1.890848 |
| | 1 2.963480 -1.024493 -1.212660 |
| | 1 1.608201 -2.025594 -0.594510 |

**Table 15.** PBE0/Def2-SVP optimised coordinates (Å) of the alanyl radical and the two amines in acetonitrile. Total energy (Hartree) in brackets. Total energy (Hartree) in brackets.

| **4C radical** (-785.3945563) | **4C product** (-786.0554701) |
|---|---|
| 8 -0.032840 1.989440 0.381255 | 8 2.068022 2.480650 0.192372 |
| 6 1.055420 1.413881 0.339782 | 6 2.303535 1.290561 0.027064 |
| 6 1.100163 -0.104528 0.537810 | 6 1.260563 0.237011 0.428374 |

| | |
|---|---|
| 7 2.398769 -0.704889 0.366496 | 7 1.759649 -1.116991 0.413070 |
| 6 2.751716 -1.351183 -0.782335 | 6 1.265105 -2.075147 -0.418543 |
| 6 4.110163 -2.001858 -0.774568 | 6 1.836815 -3.455829 -0.239517 |
| 8 2.017716 -1.387747 -1.757504 | 8 0.405165 -1.835371 -1.257387 |
| 6 0.442835 -0.512720 1.866179 | 6 0.599659 0.625370 1.753342 |
| 6 -0.461680 -1.735953 1.738259 | 6 -0.526918 -0.298037 2.210979 |
| 6 -1.662028 -1.563220 0.866963 | 6 -1.621044 -0.597087 1.183841 |
| 6 -2.415286 -0.285807 0.826598 | 6 -2.374287 0.644895 0.744517 |
| 7 -3.103859 0.018117 -0.490830 | 7 -3.232719 0.463488 -0.488190 |
| 6 -4.158856 -0.987766 -0.789065 | 6 -4.127418 -0.719663 -0.346720 |
| 6 -2.103254 0.049395 -1.594887 | 6 -2.376568 0.300809 -1.700482 |
| 6 -3.726846 1.367053 -0.369340 | 6 -4.070536 1.685698 -0.655173 |
| 7 2.220644 2.025463 0.102455 | 7 3.431973 0.816477 -0.529090 |
| 6 2.345235 3.438344 -0.146129 | 6 4.491262 1.663196 -1.011133 |
| 1 0.493722 -0.487707 -0.300350 | 1 0.511841 0.270492 -0.380362 |
| 1 3.005204 -0.784420 1.174778 | 1 2.394421 -1.404536 1.150298 |
| 1 3.992445 -3.064127 -1.031822 | 1 1.042264 -4.116954 0.137466 |
| 1 4.723971 -1.541816 -1.562645 | 1 2.142496 -3.840162 -1.222393 |
| 1 4.635946 -1.916439 0.185069 | 1 2.690017 -3.492109 0.449852 |
| 1 1.228646 -0.718110 2.610260 | 1 1.368799 0.657775 2.543470 |
| 1 -0.126005 0.339218 2.265495 | 1 0.254036 1.663442 1.640559 |
| 1 0.119757 -2.598447 1.369979 | 1 -0.101403 -1.266643 2.518294 |
| 1 -0.793413 -2.033994 2.754377 | 1 -0.977495 0.132066 3.120938 |
| 1 -2.153262 -2.475969 0.515656 | 1 -1.167542 -1.099791 0.315846 |
| 1 -3.219414 -0.259770 1.585480 | 1 -2.322563 -1.316356 1.632152 |
| 1 -1.751877 0.573708 1.003590 | 1 -3.047313 0.996433 1.539784 |
| 1 -4.672219 -0.696211 -1.712717 | 1 -1.692688 1.472107 0.502427 |
| 1 -3.698129 -1.971964 -0.922448 | 1 -4.818346 -0.738367 -1.197147 |
| 1 -4.872022 -1.014298 0.043339 | 1 -3.525884 -1.634073 -0.342380 |
| 1 -2.617390 0.302704 -2.529331 | 1 -4.690625 -0.632112 0.589767 |
| 1 -1.350407 0.809228 -1.351130 | 1 -3.025936 0.116396 -2.564128 |
| 1 -1.635224 -0.938171 -1.680658 | 1 -1.810812 1.227044 -1.856405 |
| 1 -4.236244 1.610811 -1.308606 | 1 -1.685261 -0.540024 -1.565321 |

| | |
|---|---|
| 1 -4.448429 1.354488 0.455810<br>1 -2.935181 2.098177 -0.167505<br>1 3.050458 1.443261 0.080542<br>1 2.754048 3.630753 -1.150049<br>1 1.347072 3.887535 -0.075759<br>1 3.004581 3.913354 0.596187 | 1 -4.641725 1.600459 -1.586872<br>1 -4.754037 1.769456 0.197341<br>1 -3.414683 2.562873 -0.697566<br>1 3.517297 -0.187589 -0.631320<br>1 4.627512 1.556244 -2.098902<br>1 4.223020 2.703523 -0.788837<br>1 5.445786 1.426695 -0.516274 |
| **5C radical** (-863.8731177) | **5C product** (-864.5294994) |
| 8 -0.445111 -0.836113 -1.283495<br>6 -1.564613 -0.345790 -1.163291<br>6 -1.788893 1.050261 -0.545731<br>7 -2.952476 1.040382 0.319280<br>6 -3.039270 0.204784 1.386870<br>6 -4.235930 0.397539 2.281928<br>8 -2.195027 -0.654850 1.600156<br>6 -0.531619 1.590688 0.137464<br>6 0.523543 2.118871 -0.837991<br>6 1.905849 2.264368 -0.274551<br>6 2.823539 3.277054 -0.867071<br>6 2.477764 1.141693 0.547377<br>6 2.494984 -0.148742 -0.269305<br>7 2.714792 -1.433291 0.499797<br>6 1.611701 -1.654044 1.479243<br>6 2.686093 -2.547850 -0.490645<br>6 4.025604 -1.422956 1.201608<br>7 -2.680497 -0.938839 -1.616506<br>6 -2.698339 -2.239024 -2.234011<br>1 -2.045644 1.711347 -1.390813<br>1 -3.622648 1.793631 0.229888<br>1 -4.997447 1.065504 1.859048<br>1 -4.682588 -0.584293 2.489727<br>1 -3.893065 0.812239 3.241971<br>1 -0.839073 2.403238 0.813946 | 8 0.410231 -0.976080 1.095773<br>6 1.548040 -0.513110 1.087255<br>6 1.842966 0.930711 0.631039<br>7 3.073495 0.985770 -0.133841<br>6 3.235916 0.274034 -1.279868<br>6 4.502160 0.543800 -2.051070<br>8 2.402482 -0.539065 -1.654857<br>6 0.647396 1.564847 -0.083210<br>6 -0.446839 2.035946 0.869779<br>6 -1.786005 2.382801 0.206886<br>6 -2.773095 2.919641 1.240808<br>6 -2.381444 1.222929 -0.616265<br>6 -2.545911 -0.041796 0.206876<br>7 -2.860935 -1.300840 -0.570232<br>6 -1.733665 -1.648180 -1.483355<br>6 -3.014987 -2.402928 0.422278<br>6 -4.119566 -1.159752 -1.349592<br>7 2.619940 -1.185185 1.536259<br>6 2.568268 -2.541237 2.016184<br>1 2.040191 1.497594 1.556815<br>1 3.743453 1.710211 0.091596<br>1 5.232487 1.148585 -1.498252<br>1 4.956688 -0.416265 -2.331019<br>1 4.240180 1.067372 -2.982983<br>1 1.020422 2.423031 -0.664773 |

| | |
|---|---|
| 1 -0.129359 0.792112 0.774424<br>1 0.557560 1.444307 -1.719102<br>1 0.193229 3.091980 -1.239554<br>1 2.295900 4.216913 -1.096154<br>1 3.668216 3.507598 -0.198693<br>1 3.265371 2.927459 -1.824100<br>1 3.490419 1.406810 0.885913<br>1 1.865687 1.003098 1.455458<br>1 1.528547 -0.293297 -0.772427<br>1 3.287051 -0.111902 -1.030589<br>1 0.652535 -1.573752 0.951779<br>1 1.725688 -2.654025 1.913587<br>1 1.671347 -0.905555 2.275861<br>1 1.709729 -2.539249 -0.989793<br>1 3.486011 -2.394058 -1.223765<br>1 2.837302 -3.497512 0.035615<br>1 4.033150 -0.618809 1.945384<br>1 4.170350 -2.388355 1.700944<br>1 4.821857 -1.260266 0.465486<br>1 -3.562767 -0.481737 -1.417186<br>1 -1.665800 -2.525201 -2.468374<br>1 -3.131875 -3.001182 -1.566170<br>1 -3.283792 -2.218198 -3.164959 | 1 0.265512 0.835947 -0.810940<br>1 -0.611295 1.272919 1.648116<br>1 -0.090070 2.930602 1.408567<br>1 -1.598392 3.191376 -0.522010<br>1 -2.370204 3.817379 1.734257<br>1 -3.732937 3.193801 0.776264<br>1 -2.980308 2.181002 2.032250<br>1 -3.348198 1.551876 -1.028668<br>1 -1.723467 1.034145 -1.477393<br>1 -1.614248 -0.277268 0.739589<br>1 -3.356586 0.063880 0.940938<br>1 -0.808501 -1.680794 -0.893926<br>1 -1.939279 -2.624831 -1.936689<br>1 -1.654982 -0.892926 -2.271921<br>1 -2.077981 -2.496475 0.983827<br>1 -3.839165 -2.157729 1.102021<br>1 -3.233216 -3.336218 -0.109365<br>1 -3.993897 -0.378067 -2.106109<br>1 -4.339681 -2.114763 -1.841127<br>1 -4.934461 -0.893789 -0.665905<br>1 3.523615 -0.738128 1.432698<br>1 1.516605 -2.818114 2.158353<br>1 3.023558 -3.244091 1.299561<br>1 3.095545 -2.633541 2.977118 |

**Table 16.** PBE0/Def2-SVP optimised coordinates (Å) of the radical intermediates and final products with parallel amide groups in water. Total energy (Hartree) in brackets.

| **4C radical** (-785.3949905) | **4C product** (-786.0541601) |
|---|---|
| 8 2.862324 -0.836015 -1.493249<br>6 2.802847 -0.899121 -0.270077<br>6 2.060058 0.171831 0.532455<br>7 1.933670 1.325239 -0.318412<br>6 1.737358 2.571966 0.163820 | 8 3.258021 -0.849030 -1.277516<br>6 2.865890 -0.965048 -0.121723<br>6 2.013956 0.126820 0.530740<br>7 2.161036 1.311981 -0.271554<br>6 1.904545 2.550395 0.201998 |

| | |
|---|---|
| 6 1.678063 3.671221 -0.867924 | 6 2.162126 3.686310 -0.756979 |
| 8 1.609873 2.797409 1.361698 | 8 1.483866 2.742498 1.337462 |
| 6 0.702666 -0.345194 1.039422 | 6 0.546021 -0.311384 0.681295 |
| 6 -0.256900 -0.757295 -0.070130 | 6 -0.127310 -0.676299 -0.635772 |
| 6 -1.579399 -1.212306 0.432262 | 6 -1.596409 -1.083752 -0.505966 |
| 6 -2.772241 -1.283271 -0.443459 | 6 -2.470503 0.098024 -0.130735 |
| 7 -3.865829 -0.263859 -0.117613 | 7 -3.948078 -0.186792 0.014327 |
| 6 -3.328242 1.118460 -0.239800 | 6 -4.195551 -1.178542 1.098979 |
| 6 -4.371142 -0.481822 1.265532 | 6 -4.525419 -0.690637 -1.264023 |
| 6 -4.982181 -0.448801 -1.084611 | 6 -4.622739 1.094466 0.373044 |
| 7 3.328715 -1.899176 0.457346 | 7 3.103716 -2.040627 0.648120 |
| 6 4.009643 -3.028954 -0.123466 | 6 3.837652 -3.195170 0.194753 |
| 1 2.658924 0.449605 1.416037 | 1 2.403508 0.334483 1.542033 |
| 1 2.100060 1.153347 -1.306217 | 1 2.568315 1.162626 -1.190844 |
| 1 1.842328 3.319579 -1.894738 | 1 2.563532 3.361370 -1.725535 |
| 1 0.694068 4.158902 -0.807476 | 1 1.220084 4.229610 -0.921450 |
| 1 2.433005 4.430742 -0.618751 | 1 2.865891 4.390225 -0.289828 |
| 1 0.871835 -1.191926 1.724733 | 1 0.495229 -1.164838 1.378085 |
| 1 0.256388 0.461315 1.642040 | 1 0.030149 0.525651 1.177365 |
| 1 -0.384702 0.075574 -0.784581 | 1 -0.038444 0.166555 -1.342067 |
| 1 0.196816 -1.572992 -0.673063 | 1 0.409178 -1.518500 -1.101727 |
| 1 -1.636965 -1.666844 1.426588 | 1 -1.922004 -1.504040 -1.469227 |
| 1 -2.511685 -1.106368 -1.496585 | 1 -1.680302 -1.887058 0.243191 |
| 1 -3.271399 -2.262079 -0.373420 | 1 -2.163078 0.530952 0.830815 |
| 1 -4.128411 1.830266 -0.006437 | 1 -2.392312 0.888533 -0.891155 |
| 1 -2.499919 1.242601 0.467395 | 1 -5.277798 -1.289905 1.230736 |
| 1 -2.976097 1.275713 -1.265938 | 1 -3.758906 -2.143099 0.821580 |
| 1 -5.185020 0.226352 1.458603 | 1 -3.742206 -0.811384 2.027166 |
| 1 -4.740827 -1.510284 1.351491 | 1 -5.608590 -0.800430 -1.138960 |
| 1 -3.555458 -0.314009 1.977189 | 1 -4.312503 0.032462 -2.060013 |
| 1 -5.768074 0.282602 -0.863115 | 1 -4.085122 -1.662410 -1.508845 |
| 1 -4.603401 -0.294113 -2.101528 | 1 -5.697418 0.911002 0.485079 |
| 1 -5.377956 -1.465933 -0.982838 | 1 -4.204598 1.464612 1.316115 |

| | |
|---|---|
| 1 3.269801 -1.843107 1.467005<br>1 3.526140 -3.974534 0.165668<br>1 3.969858 -2.926749 -1.214553<br>1 5.064041 -3.064520 0.191504 | 1 -4.447870 1.823747 -0.426062<br>1 2.774585 -2.028301 1.605884<br>1 3.228371 -4.108499 0.273333<br>1 4.108208 -3.036352 -0.855972<br>1 4.758744 -3.337785 0.780832 |
| **5C radical** (-863.8772187) | **5C product** (-864.5336735) |
| 8 -0.266446 -1.281820 -0.349081<br>6 -1.367482 -0.789169 -0.591861<br>6 -1.763692 0.626041 -0.113754<br>7 -2.746935 0.537943 0.950901<br>6 -4.068524 0.404364 0.708127<br>6 -4.976079 0.426953 1.907776<br>8 -4.508091 0.275607 -0.434152<br>6 -0.551049 1.427024 0.331929<br>6 0.320631 1.888634 -0.838571<br>6 1.720914 2.291111 -0.485126<br>6 2.408480 3.319256 -1.315200<br>6 2.545106 1.378509 0.381252<br>6 2.696153 0.017584 -0.293785<br>7 3.305459 -1.088829 0.538547<br>6 2.445403 -1.388006 1.719932<br>6 3.358096 -2.306508 -0.320853<br>6 4.679501 -0.736354 0.984710<br>7 -2.338660 -1.415354 -1.271349<br>6 -2.234909 -2.770991 -1.745574<br>1 -2.264565 1.128466 -0.958418<br>1 -2.420634 0.586757 1.908843<br>1 -4.443372 0.537430 2.860904<br>1 -5.685581 1.259209 1.792354<br>1 -5.563562 -0.502147 1.923652<br>1 -0.895471 2.303307 0.902519<br>1 0.026965 0.797654 1.025505<br>1 0.355715 1.077118 -1.597276 | 8 -0.273142 -1.278376 -0.308557<br>6 -1.372707 -0.791646 -0.568998<br>6 -1.782197 0.624434 -0.105131<br>7 -2.785684 0.538475 0.941178<br>6 -4.101968 0.398975 0.675096<br>6 -5.031925 0.426474 1.857424<br>8 -4.520303 0.261031 -0.474196<br>6 -0.582219 1.434389 0.358914<br>6 0.321286 1.868631 -0.790596<br>6 1.702342 2.392287 -0.376591<br>6 2.472674 2.892662 -1.596292<br>6 2.520114 1.382684 0.454081<br>6 2.681313 0.050411 -0.254351<br>7 3.291942 -1.066626 0.561819<br>6 2.431821 -1.390432 1.737035<br>6 3.351105 -2.270061 -0.317021<br>6 4.664568 -0.719427 1.016981<br>7 -2.331315 -1.425577 -1.259391<br>6 -2.214797 -2.782781 -1.725859<br>1 -2.268411 1.120368 -0.962118<br>1 -2.477416 0.596851 1.904567<br>1 -4.517445 0.541588 2.819954<br>1 -5.739602 1.257763 1.724955<br>1 -5.619072 -0.502914 1.866406<br>1 -0.946980 2.321756 0.900426<br>1 -0.026544 0.820450 1.084672<br>1 0.447662 1.033864 -1.501062 |

| | |
|---|---|
| 1 -0.179022 2.726823 -1.351595<br>1 1.721612 4.128975 -1.608570<br>1 3.266441 3.765601 -0.787756<br>1 2.807782 2.891523 -2.258979<br>1 3.527991 1.836642 0.566720<br>1 2.057439 1.258537 1.363966<br>1 1.710330 -0.370469 -0.585772<br>1 3.324407 0.102144 -1.191549<br>1 1.422000 -1.563391 1.364195<br>1 2.839936 -2.279345 2.221657<br>1 2.469295 -0.541813 2.414174<br>1 2.336296 -2.554308 -0.631861<br>1 3.979412 -2.097328 -1.199160<br>1 3.789538 -3.132841 0.255665<br>1 4.635197 0.136729 1.644435<br>1 5.103450 -1.587259 1.530736<br>1 5.293837 -0.512210 0.104556<br>1 -3.233528 -0.923585 -1.334193<br>1 -1.212875 -3.126936 -1.567741<br>1 -2.937602 -3.435814 -1.218223<br>1 -2.449473 -2.826782 -2.823630 | 1 -0.189052 2.660547 -1.364080<br>1 1.543219 3.257719 0.290949<br>1 1.917939 3.696838 -2.103369<br>1 3.458427 3.292096 -1.312119<br>1 2.633842 2.093505 -2.338039<br>1 3.500730 1.833354 0.673405<br>1 2.018708 1.240056 1.422844<br>1 1.702183 -0.341939 -0.561412<br>1 3.317656 0.147515 -1.144778<br>1 1.408696 -1.559510 1.377396<br>1 2.827787 -2.291124 2.220467<br>1 2.455072 -0.558402 2.448053<br>1 2.330687 -2.517600 -0.632612<br>1 3.971920 -2.043586 -1.191414<br>1 3.786395 -3.103429 0.246213<br>1 4.617286 0.139710 1.694379<br>1 5.089812 -1.580539 1.545653<br>1 5.279365 -0.475867 0.142306<br>1 -3.226740 -0.937196 -1.339380<br>1 -1.196912 -3.136933 -1.522398<br>1 -2.929185 -3.446182 -1.212685<br>1 -2.403544 -2.843505 -2.808544 |

**Table 17.** PBE0/Def2-SVP optimised coordinates (Å) of the radical intermediates and final products with anti-parallel amide groups in water. Total energy (Hartree) in brackets.

| **Alanyl radical** (-494.2826922) | **H atom** (-0.5003066) |
|---|---|
| 8 -2.340004 0.811738 -0.573870<br>6 -1.368016 0.284525 -0.051815<br>6 -0.133033 1.094853 0.406261<br>7 1.074836 0.699386 -0.309190<br>6 1.842747 -0.342608 0.071965<br>6 3.115692 -0.556671 -0.700749 | |

| | |
|---|---|
| 8 1.516329 -1.082608 1.000069<br>6 -0.356797 2.555736 0.306357<br>7 -1.257496 -1.032539 0.201450<br>6 -2.262302 -1.994350 -0.167392<br>1 0.048005 0.802593 1.461221<br>1 1.346024 1.254288 -1.112407<br>1 3.298542 0.206267 -1.467909<br>1 3.955647 -0.566713 0.008563<br>1 3.078087 -1.547977 -1.175345<br>1 0.456453 3.241795 0.550579<br>1 -1.346274 2.933982 0.050198<br>1 -0.390896 -1.344925 0.644732<br>1 -3.114111 -1.456075 -0.601535<br>1 -1.881575 -2.712567 -0.911463<br>1 -2.611631 -2.560547 0.710189 | |
| **Amine 4C** (-291.0598153) | **Amine 5C** (-369.5393793) |
| 6 2.758827 -0.280823 0.053482<br>6 1.698592 0.490191 -0.195669<br>6 0.463467 -0.018343 -0.862625<br>7 -0.775000 -0.023399 0.015723<br>6 -1.081752 1.352398 0.498804<br>6 -0.574522 -0.927279 1.182443<br>6 -1.917443 -0.520184 -0.802387<br>1 3.667194 0.134907 0.497000<br>1 2.762442 -1.349289 -0.185421<br>1 1.742733 1.559471 0.036753<br>1 0.202674 0.601712 -1.733151<br>1 0.596648 -1.053431 -1.204805<br>1 -2.024233 1.325854 1.057779<br>1 -0.275412 1.694790 1.155714<br>1 -1.176627 2.021246 -0.364747<br>1 -1.480776 -0.912148 1.798351<br>1 -0.385790 -1.943431 0.816889 | 6 -3.013320 1.173868 0.250861<br>6 -2.265439 0.109592 -0.064639<br>6 -2.766939 -1.298233 0.068815<br>6 -0.858919 0.285686 -0.594343<br>6 0.161973 -0.056408 0.481284<br>7 1.611490 -0.009269 0.053786<br>6 1.971901 1.339902 -0.467518<br>6 2.447636 -0.302777 1.254409<br>6 1.893129 -1.036261 -0.988849<br>1 -2.634766 2.193597 0.133036<br>1 -4.031811 1.058464 0.632746<br>1 -3.793719 -1.324061 0.458390<br>1 -2.753282 -1.809466 -0.908427<br>1 -2.134904 -1.899084 0.743600<br>1 -0.727993 -0.366369 -1.473360 |

| | |
|---|---|
| 1 0.284442 -0.574773 1.763920 | 1 -0.728639 1.325252 -0.926254 |
| 1 -2.816125 -0.550855 -0.176035 | 1 0.071641 0.644075 1.323094 |
| 1 -2.072932 0.159884 -1.647707 | 1 0.003888 -1.070990 0.872315 |
| 1 -1.682253 -1.525909 -1.168889 | 1 1.718636 2.094324 0.286540 |
| | 1 3.048700 1.360515 -0.669781 |
| | 1 1.422750 1.532683 -1.394605 |
| | 1 2.262909 0.465888 2.013391 |
| | 1 2.173956 -1.289312 1.645553 |
| | 1 3.504033 -0.296244 0.961920 |
| | 1 1.323295 -0.806456 -1.894691 |
| | 1 2.964915 -1.017623 -1.216443 |
| | 1 1.609834 -2.022526 -0.603646 |

**Table 18.** PBE0/Def2-SVP optimised coordinates (Å) of the alanyl radical and the two amines in water. Total energy (Hartree) in brackets.
